# *Asmodochelys parhami*, a new fossil marine turtle from the Campanian Demopolis Chalk and the stratigraphic congruence of competing marine turtle phylogenies

**DOI:** 10.1098/rsos.191950

**Published:** 2019-12-18

**Authors:** Andrew D. Gentry, Jun A. Ebersole, Caitlin R. Kiernan

**Affiliations:** 1 Department of Biology, University of Alabama at Birmingham, Birmingham, AL 35233, USA; 2 Department of Collections, McWane Science Center, Birmingham, AL 35203, USA

**Keywords:** sea turtle, evolution, biostratigraphy, gap excess ratio, Chelonioidea, phylogeny

## Abstract

Resolving the phylogeny of sea turtles is uniquely challenging given the high potential for the unification of convergent lineages due to systematic homoplasy. Equivocal reconstructions of marine turtle evolution subsequently inhibit efforts to establish fossil calibrations for molecular divergence estimates and prevent the accurate reconciliation of biogeographic or palaeoclimatic data with phylogenetic hypotheses. Here we describe a new genus and species of marine turtle, *Asmodochelys parhami*, from the Upper Campanian Demopolis Chalk of Alabama and Mississippi, USA represented by three partial shells. Phylogenetic analysis shows that *A. parhami* belongs to the ctenochelyids, an extinct group that shares characteristics with both pan-chelonioids and pan-cheloniids. In addition to supporting Ctenochelyidae as a sister taxon of Chelonioidea, our analysis places Protostegidae outside of the Chelonioidea crown group and recovers *Allopleuron hofmanni* as a stem dermochelyid. Gap excess ratio (GER) results indicate a strong stratigraphic congruence of our phylogenetic hypothesis; however, the highest GER value is associated with the phylogenetic hypothesis of marine turtles which excludes Protostegidae from the Cryptodira crown group. Ancestral range estimations derived from our phylogeny imply a European or North American origin of Chelonioidea in the middle-to-late Campanian, approximately 20 Myr earlier than current molecular divergence studies suggest.

## 1. Introduction

Recent studies have demonstrated that the incorporation of palaeontological data improves forecasts of biodiversity responses to climate change [1–3]. Refining these predictions for any particular taxon therefore relies upon a firm understanding of its evolutionary history. With an extensive fossil record spanning more than 90 Myr, sea turtles (Chelonioidea) are the oldest living marine tetrapod lineage [4], and with modern species being focal taxa for global conservation efforts, chelonioids provide a prime model for this type of integrative approach to biodiversity risk assessment. Unfortunately, the phylogenetic positions of many fossil chelonioids remain poorly justified, resulting in a lack of definitive fossil calibrations for molecular divergence estimates [5]. The subsequent uncertainty surrounding the evolution of chelonioids hinders efforts to produce well-resolved phylogenetic hypotheses that can be coupled with marine geochemical proxies for palaeoclimatic shifts (e.g. C, Sr and O isotope records).

The most problematic group with regard to the composition of total group Chelonioidea is Protostegidae [6]. Often recovered as highly derived chelonioids [7–9], the fossil occurrence of the oldest protostegid, *Desmatochelys padillai*, pre-dates that of the earliest unambiguous non-protostegid total group chelonioid, *Toxochelys latiremis*, by approximately 30 Myr [10,11]. The inclusion of the protostegids into Chelonioidea also necessitates the existence of a nearly 50 Myr ghost lineage for the earliest fossil chelydroid, as molecular evidence strongly supports a sister relationship between chelonioids and Chelydroidea [12,13]. It has been argued that the recovery of protostegids as crown group chelonioids in many phylogenetic analyses is the result of homoplasy due to the inclusion of characters tied to convergent marine specializations in turtle character-taxon matrices and that protostegids represent an earlier, distinct radiation of marine-adapted turtles [11,14]. More recent studies have indicated that protostegids may be stem chelonioids [15,16], a scenario that would significantly reduce the implied ghost lineages for the clades comprising the chelonioid crown group. The true relationship between protostegids and crown chelonioids can only be resolved through the further refinement of turtle character-taxon matrices and the inclusion of additional fossil chelonioids into global phylogenetic studies.

Here we describe a new genus and species of fossil chelonioid, *Asmodochelys parhami*, from the Upper Campanian Demopolis Chalk (79–74.5 Ma [17]) of the Gulf Coastal Plain, USA. This new taxon is included in an expanded phylogenetic analysis of turtles which indicates that *Asmodochelys* belongs to the extinct Ctenochelyidae, a pan-chelonioid group characterized by a laterally serrated shell, extensive costal and plastral fontanelles, and the presence of epineurals positioned at various intervals along the neural series. Our analysis also recovers a novel phylogeny for marine turtles that, when combined with stratigraphic and biogeographic evidence, supports a North American or European origin of crown group Chelonioidea in the middle-to-late Campanian.

## 2. Material and methods

Three specimens of the new stem chelonioid are known, all from the Upper Campanian Demopolis Chalk of Alabama and Mississippi, USA. Bayesian and parsimony phylogenetic analysis were used to establish the phylogenetic position of the new taxon. Our matrix was constructed using a modified version of the Evers & Benson [9] character-taxon matrix, which greatly expanded on previous matrices [11,18–20] and samples marine turtles the most densely by far (see electronic supplementary material for complete character list). The scorings and character definitions follow those of Evers & Benson [9] with the following exceptions:

(1) The addition of six fossil species: *Toxochelys latiremis*, *Ctenochelys stenoporus*, *Ctenochelys acris*, *Prionochelys matutina*, *Peritresius ornatus*, *Euclastes wielandi* and *Asmodochelys parhami* (see electronic supplementary material for sources of character scoring; electronic supplementary material, table S1).
(2) The creation of two new characters: ch. 203: the presence of epineurals; ch. 309: maximum width of coracoid posterior process.
(3) Nine revised character definitions: ch. 55, ch. 65, ch. 93, ch. 103, ch. 119, ch. 212, ch. 213, ch. 314, ch. 325.
(4) We rescore three characters for *Allopleuron hofmanni* (ch. 211, ch. 218, ch. 314), one character for *Protostega gigas* (ch. 211), three characters for *Lepidochelys olivacea* (ch. 182, ch. 201, ch. 202), one character for *Caretta caretta* (ch. 314), and two characters for *Chelonia mydas*, *Lepidochelys kempii* and *Natator depressus* (ch. 201, ch. 202). See electronic supplementary material for sources of character scoring.

These modifications resulted in a matrix of 87 species and 347 characters (electronic supplementary material, data S1). The matrix was edited using Mesquite v. 3.6 build 917 [21]. Of the 347 total characters, 24 multistate characters were treated as ordered. Parsimony analyses were conducted in PAUP* v. 4.0a build 165 [22] using the heuristic search algorithm with 10 000 random addition sequence replicates of starting trees obtained by simple stepwise addition and the tree bisection and reconnection method of branch swapping. Characters scored as multiple states for any species were treated as polymorphisms, and branches with a maximum length = 0 were set to collapse. Bremer decay index (BDI) values were calculated by retaining trees with sequentially higher step values than the most parsimonious trees (MPTs) until all but the most well-supported bipartitions (BDI ≥ 6) had collapsed. Bayesian analysis was conducted in MrBayes v. 3.2 [23] using a general-time reversible substitution type and an Mk model of rate variation with ascertainment bias correction. Model parameters, posterior distribution and branch lengths were estimated with Markov chain Monte Carlo, using four chains of 10 000 000 generations with sampling every 1000 generations. Analyses were run until the average standard deviation of the split frequencies was below 0.01. The first 25% of samples were discarded as burn-in. In both the parsimony and Bayesian analyses, *Proganochelys quenstedti* was set as the outgroup and the positions of extant species were constrained using a molecular ‘backbone’ (electronic supplementary material, figure S16) derived from a global phylogenomic analysis of turtles [13]. To reduce the potential for chimaeric operational taxonomic units (OTUs), supraspecific OTUs were excluded. Phylogenetic nomenclature follows Joyce *et al*. [24]. Ancestral biogeographic areas of nodes within Pan-Chelonioidea were inferred using probability calculations following the rules of multiplication and addition (see electronic supplementary material). Ancestral area probabilities were successively calculated from the tip to the base of the strict consensus tree derived from weighted parsimony analysis. Ancestral areas for each species-level taxonomic unit were restricted to the continent where the oldest material confidently assigned to that species was recovered.

## 3. Systematic palaeontology

Testudines [25]

Cryptodira [26]

Pan-Chelonioidea [24]

Ctenochelyidae [27]

*Asmodochelys parhami* n. gen. et sp.

urn:lsid:zoobank.org:act: 147C2B3C-F3A2-4818-879E-452ADE2C4DE3

urn:lsid:zoobank.org:act: EFADE61D-7F5E-4074-8B5F-D0AC7D470E4F

### 3.1. Etymology

*Asmodo* from the Greek ‘Asmodaios’, the horned deity and Master of the Sea who, according to Islamic legend, was entombed in stone on the ocean floor [28] and *chelys* from the Greek word for turtle. The species name honours James F. Parham, former Curator of Palaeontology at the Alabama Museum of Natural History for his contributions to Alabama palaeontology and the study of marine turtle evolution.

### 3.2. Holotype

**Figure 1.**
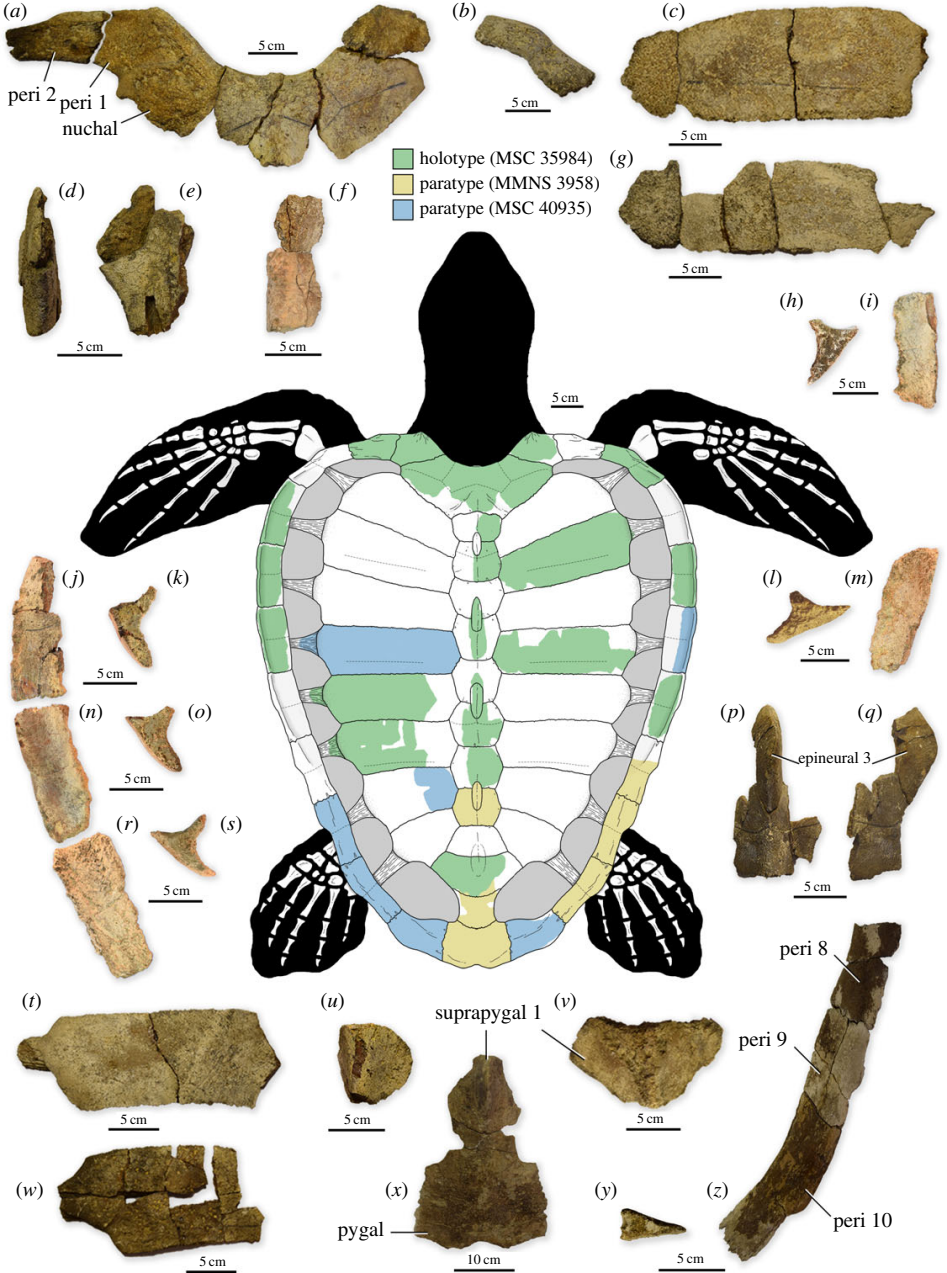
Representative elements and composite reconstruction of *Asmodochelys parhami*. (*a*) Nuchal, first and second left peripherals, and first right peripheral (MSC 35984) in dorsal view. (*b*) Third right peripheral (MSC 35984) in dorsal view. (*c*) Second right costal (MSC 35984) in dorsal view. (*d,e*) Fourth neural and second epineural (MSC 35984) in dorsal (*d*) and left lateral (*e*) views. (*f*) First and second neural (MSC 35984) in dorsal view. (*g*) Fourth right costal (MSC 35984) in dorsal view. (*h,i*) Fifth right peripheral in posterior (*h*) and dorsal (*i*) views. (*j,k*) Fourth left peripheral (MSC 35984) in dorsal (*j*) and posterior (*k*) views. (*l,m*) Seventh right peripheral (MSC 35984) in posterior (*l*) and dorsal (*m*) views. (*n,o*) Fifth left peripheral (MSC 35984) in dorsal (*n*) and posterior (*o*) views. (*p,q*) Sixth neural and third epineural (MSC 35984) in dorsal (*p*) and right lateral (*q*) views. (*r,s*) Sixth left peripheral (MSC 35984) in dorsal (*r*) and posterior (*s*) views. (*t*) Fifth left costal (MSC 35984) in dorsal view. (*u*) Seventh neural (MSC 35984) in dorsal view. (*v*) First suprapygal (MSC 35984) in dorsal view. (*w*) Sixth right costal (MSC 35984) in dorsal view. (*x*) Suprapygals and pygal (MMNS 3958) in dorsal view. (*y*) Tenth left peripheral (MMNS 3958) in posterior view. (*z*) Eighth–tenth right peripherals (MMNS 3958) in dorsal view. Dashed lines represent scute sulci.

MSC (McWane Science Center, Birmingham, AL) 35984. A single individual preserving the nuchal, four neurals, two epineurals, eight left peripherals, five right peripherals, a partial first suprapygal, two costals of the left side, two costals of the right side, approximately half of the left hyoplastron and one cervical vertebra (figure 1).

### 3.3. Type locality and horizon

Town of Alberta, Wilcox County, AL, USA. ‘Muldrow’ Member of the Demopolis Chalk, Upper Campanian (see electronic supplementary material for detailed locality information).

### 3.4. Paratypes

MMNS (Mississippi Museum of Natural Sciences, Jackson, MS) 3958, site MS.53.017, Oktibbeha County, Mississippi, USA, ‘Muldrow’ Member of the Demopolis Chalk, Upper Campanian. This specimen preserves two left peripherals, one neural, one epineural, both suprapygals and a complete pygal (figure 1). MSC 40935, site ASu-14, Sumter County, Alabama, USA, Bluffport Marl Member of the Demopolis Chalk, Upper Campanian. This specimen consists of one complete costal, three medial peripherals, four posterior peripherals, three neurals, two epineurals, the first suprapygal and the left xiphiplastron (electronic supplementary material, figure S7; see electronic supplementary material for additional locality and specimen information).

### 3.5. Diagnosis

Thick shell with a deep nuchal embayment; nuchal fontanelles absent; horn-like protuberance on the anterodorsal edge of the first peripheral; concave dorsal plates of peripherals 4–8 resulting in the formation of a pronounced peripheral gutter; extreme reduction in height and width of posterior peripherals; anterior and posterior neurals wider than long; four neural keel elevations with epineurals dorsal to the junctions of neurals 1–2, 2–3, 4–5, 6–7; single keel elevation dorsal to the first suprapygal terminating immediately anterior to the second suprapygal; dorsal facet of the pygal considerably longer than the ventral facet; distinct notch at the posterior margin of the pygal.

### 3.6. Comparative diagnosis

*Asmodochelys parhami* can be distinguished from all previously described ctenochelyid turtles by the following carapacial characteristics: (i) The nuchal embayment of *Ctenochelys* and *Peritresius* receives only minimal contributions from the medial margins of the left and right first peripheral, whereas more than half of the nuchal embayment of *Asmodochelys* is formed by the first peripherals. (ii) Nuchal fontanelles are present in *Ctenochelys*, *Prionochelys* and *Peritresius*, whereas these features are absent in *Asmodochelys*. (iii) The lateral peripherals of *Asmodochelys* are widest at the level of the suture between the second and third costal plate differing from the condition observed in *Ctenochelys* and *Prionochelys* where the peripherals are widest along the posterior edge of the carapace. (iv) The epineural dorsal to the contact between the first and second neural of *Asmodochelys* is absent in *Ctenochelys*, *Prionochelys* and *Peritresius*. (v) Additional characters from the diagnosis are unknown in *Ctenochelys*, *Prionochelys* and *Peritresius* such as the extreme reduction in the size of the posterior peripherals, the horn-like protuberance on the anterodorsal edge of the first peripheral, and the varying length of the dorsal and ventral facets of the pygal.

## 4. Description

The carapace of *Asmodochelys* is strongly cordiform and is much longer than wide (maximum carapace length = ∼1.0–1.5 m) with the widest point being at the level of the fifth peripheral (figure 1). Due to the posterior convexity of the nuchal, the suture between the nuchal and first peripheral lies at a 100°–110° angle with the sagittal midline of the carapace, differing from other known ctenochelyids, including *Ctenochelys* [29], *Prionochelys* [10,27] and *Peritresius* [30]. A raised pedestal preserved on the visceral surface of the nuchal probably served as an articulation site for the dorsal process of the eighth cervical vertebra, a trait proposed as an apomorphy of pan-chelonioids [14,31]. The nuchal bears a slight ridge running along the dorsal midline beginning immediately posterior to the posteromedial edge of the cervical scute which increases in camber as it progresses posteriorly towards the sutural articulation with the first neural. The costo-nuchal sutures span the majority of the posterolateral margins of the nuchal and extend anterolaterally, terminating immediately posterior to the contact between the nuchal and first peripheral. Based on the extent of the costo-nuchal sutures and morphology of the articulation between the nuchal and first neural, there is no indication that nuchal fontanelles were present, differing from the condition seen in other ctenochelyid marine turtles (e.g. *Ctenochelys*, *Prionochelys* and *Peritresius*) where these fontanelles are found in all ontogenetic stages [27]. The cervical scute of *Asmodochelys* roughly resembles that of *Ctenochelys stenoporus* [29], forming an irregular heptagonal polygon. However, the cervical scute of *Asmodochelys* is proportionally longer and covers a much larger percentage of the dorsal surface of the nuchal. The nuchal bears a pronounced medial embayment which extends laterally approximately half of the total width of the nuchal and receives significant contributions from the anteromedial edge of both the left and right first peripherals (figure 1*a*) similar to the condition observed in *Allopleuron hofmanni* from the Maastrichtian of Europe [32]. The anterior margin of the first peripheral bears a dorsally oriented, horn-like protuberance (a distinctive character of *Asmodochelys*) that forms the lateral-most extent of the nuchal embayment (figure 1*a*). The presence of this feature is unknown among the Late Cretaceous chelonioids of North America but has been noted to a lesser extent in *Al. hofmanni*. The dorsolateral and ventral surfaces of the medial peripherals are widely separated (figure 1*h–o,r,s*), forming a high, proximally facing sulcus which runs from the posterior half of the third peripheral to the anterior half of the eighth, resembling the condition observed in *Peritresius* [30]. Dorsally, the anteromedial peripherals bear a ventrally convex trough which terminates on the dorsal surface of the eighth peripheral. The dorsoventral height and mediolateral width of the posterior peripherals are greatly reduced (figure 1*y,z*), similar to those of *Al. hofmanni* [32]*.* The reduction in width of the posterior peripherals distinguishes *Asmodochelys* from other ctenochelyids such as *Ctenochelys* and *Prionochelys*, whose peripherals widen posteriorly along the series and continue to increase in width during ontogeny. Based on the estimated size of the carapace and the presence of laterally expanded costal plates (figure 1*c*,*g*,*t*,*w*) in both of the most complete specimens of *Asmodochelys* (MSC 35984 and MSC 40935), it is likely that these specimens represent mature individuals and that the relative proportions of the peripheral series would not differ significantly in a more ontogenetically advanced individual.

The neural series of *Asmodochelys* comprises nine neurals and four epineurals (figure 1*d–f*,*p*,*q*,*u*). The morphology of the generally hexagonal, dorsally keeled neurals of *Asmodochelys* resembles that of the pan-chelonioids *Ctenochelys* and *Prionochelys* but differs in that the width of each neural often equals or exceeds its length, similar to those of *Peritresius ornatus* [30]. Vertebral scale sulci are visible on the dorsal surface of neurals two and six (figure 1*f*,*p*). The neurals of *Asmodochelys* lack the distinctive dermal sculpturing of *Pe. ornatus* although the external surface is marked by numerous vascular innervations, though somewhat less prominent than those observed in both modern cheloniids [33] and on the costal plates of an unnamed Oligocene pan-cheloniid [34]. In ventral aspect, the neurals of *Asmodochelys* possess an extensive layer of notably osteoporotic trabecular bone. The epineurals dorsal to the neural series form four distinct elevations along the midsagittal keel of the carapace (figure 1), somewhat similar to the epineurals of *Ctenochelys* and *Prionochelys*. However, the presence of an epineural between the first and second neural distinguishes *Asmodochelys* from other ctenochelyids.

The first suprapygal is roughly triangular, tapering in width posteriorly (figure 1*v*,*x*), resembling the first suprapygal of *Peritresius*. The first suprapygal of MMNS 3958 bears a dorsally rounded keel elevation possibly comprising one or more episuprapygals (a feature observed in other ctenochelyids), but due to poor preservation, this arrangement cannot be determined with any confidence. The second suprapygal is much narrower than the first and contacts the pygal posteriorly along a broadly concave transverse suture (figure 1*x*). The dorsal plate of the pygal is remarkably long (approx. 1.5 times the length of the ventral plate; electronic supplementary material, figure S5), differing from the equally long ventral and dorsal pygal surfaces of the Santonian-Campanian pan-chelonioids (e.g. *Toxochelys*, *Ctenochelys*) and the equally short pygal surfaces observed in the predominantly Maastrichtian pan-chelonioids such as *Allopleuron* and *Peritresius*. Overall, the carapacial elements of *Asmodochelys* are remarkably robust in their general construction owing primarily to a 3–5 mm thick layer of dense external cortical bone (electronic supplementary material, figure S8). The reduction of the compact external cortex and the homogenization of cortical and interior trabecular bone found in *Ctenochelys* and *Toxochelys* [33] is absent in *Asmodochelys*. The compact, well-vascularized external cortex of *Asmodochelys* more closely resembles the condition seen in *Al. hofmanni* and may be indicative of a near-shore marine ecology [33,35]. One procoelous cervical vertebra is preserved with MSC 35984 (electronic supplementary material, figure S6) bearing a pronounced longitudinal keel along the ventral surface of the centrum, a previously proposed synapomorphy of pan-chelonioids [11].

## 5. Phylogenetic analysis

The phylogenetic position of *Asmodochelys* was tested with both parsimony and Bayesian phylogenetic inference. The unweighted parsimony analysis retrieved 281 MPTs with a length of 1595 steps, consistency index of 0.29, retention index of 0.67 and homoplasy index of 0.73. The strict consensus of these MPTs places *Asmodochelys* as a basal member of Ctenochelyidae with *Ctenochelys*, *Prionochelys* and *Peritresius*, together forming a sister clade to Chelonioidea (electronic supplementary material, figure S17). Ctenochelyidae is supported by five unambiguous synapomorphies: (i) a moderate contribution to the upper triturating surface by the palatine (ch. 55), (ii) shallow ridge on the ventral surface of the vomer (ch. 65), (iii) a domed shape contribution of the vomer to palate roof (ch. 66), (iv) the presence of epineurals (ch. 203), and (v) the lateral process of the humerus being slightly separated from the caput humeri (ch. 325). Certain ctenochelyids have historically been recovered as sister taxa to *Toxochelys* on either the stem of Chelonioidea [8,36] or within Pan-Cheloniidae [7,32,37–40]. *Toxochelys* was recovered here as a stem chelonioid and sister taxon to the clade formed by Ctenochelyidae and crown group Chelonioidea. In contrast with many previous analyses [8,9,11,37,41,42], Protostegidae is recovered as a clade of stem chelonioids supported by five unambiguous synapomorphies: (i) the presence of a medial contact of the palatines (ch. 62), (ii) pterygoids contact the medial edge of the mandibular condyle facet (ch. 103), (iii) strongly serrated lateral and medial margins of the plastron (ch. 237), (iv) expansion of the lateral process onto the ventral surface of the humerus (ch. 330), and (v) lateral process of the humerus with prominent anterior projection (ch. 331). Our analysis also recovers *Allopleuron* as a basal member of Pan-Dermochelys, the sister clade to Pan-Cheloniidae, that together form Chelonioidea. Pan-Dermochelys is supported by three unambiguous synapomorphies: (i) contact between the jugal and squamosal (ch. 25), (ii) no contact between the postorbital and quadratojugal (ch. 42), and (iii) an absence of plastral scutes (ch. 257). Chelonioidea is supported by five unambiguous synapomorphies: (i) contact between the parietal and squamosal (ch. 15), (ii) the absence of a parasagittal ridge on the palatal surface of the pterygoid (ch. 104), (iii) rod-like rostrum basisphenoidale (ch. 138), (iv) vertical median ridge on the anterior surface of the dorsum sellae (ch. 140), and (v) humerus with a V-shaped lateral process (ch. 329).

**Figure 2.**
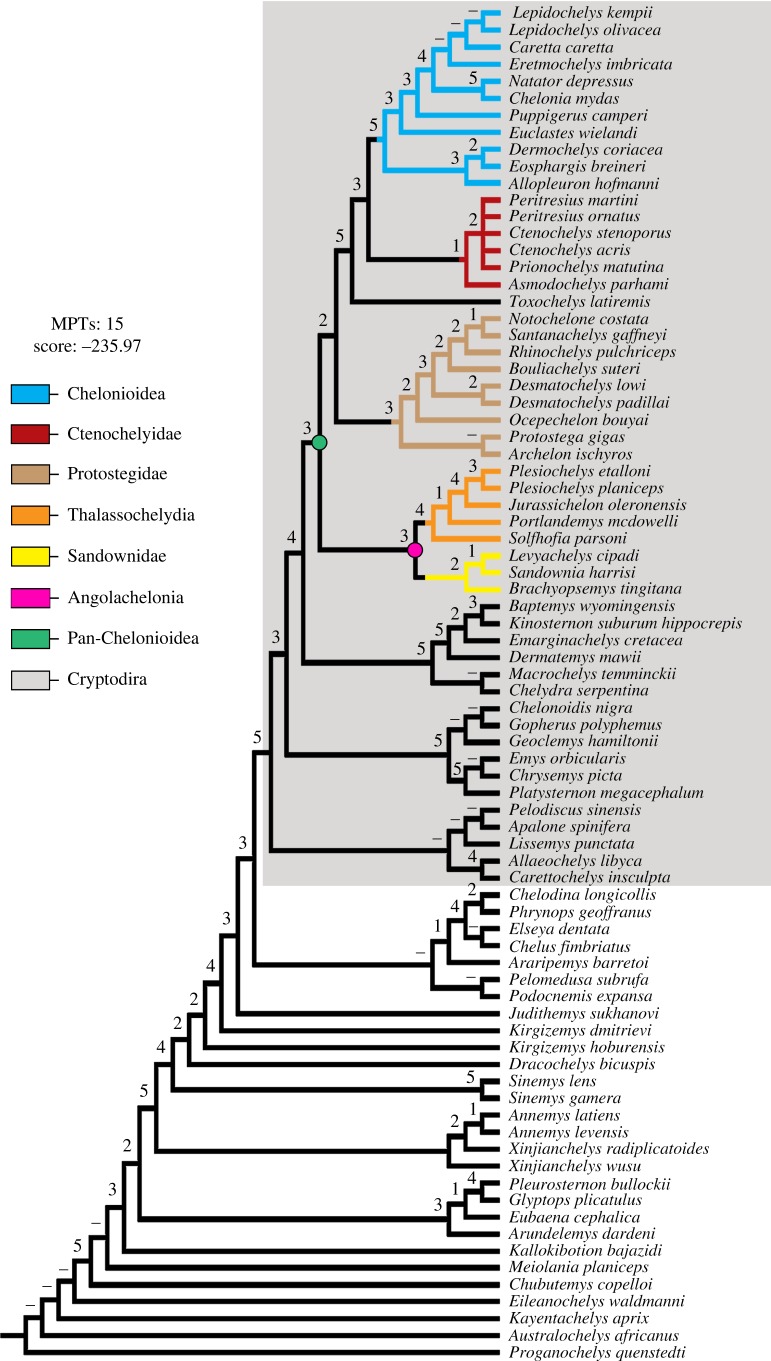
Simplified strict consensus tree from weighted parsimony analysis. Bremer support values are shown for each resolved node. Dashes indicate Bremer support values greater than or equal to 6. MPTs, most parsimonious trees.

A growing body of evidence is available which suggests that implementing mild implied weighting improves the results of cladistic analyses using parsimony [43–45]. To test the influence of implied weighting, a second tree search was conducted using mild weighting with a *k* factor of 12. This analysis retrieved tree topologies very similar to those obtained in the unweighted analysis, with the only exceptions being the removal of the macrobaenid *Judithemys* from the crown of Cryptodira and *Solnhofia* being recovered as a member of Thalassochelydia (figure 2). As we use only a mild weighting and as the placement of these taxa in the overall topology recovered by the weighted analysis is more consistent with previous cladistic studies of Thalassochelydia [19,46] and Macrobaenidae [47], we consider the weighted topology to be preferable. Additionally, branch support of clades recovered in the weighted parsimony analysis, evaluated using Bremer support, were generally higher than those recovered in the unweighted analysis. Bremer values higher than one were recovered for Angolachelonia (3), Protostegidae (3), the clade formed by *Toxochelys* and more derived pan-chelonioids (5), Pan-Dermochleys (3) and Chelonioidea (5).

Overall, the tree topologies recovered by the Bayesian analyses were similar to those of the parsimony analyses, with Protostegidae outside of crown group Chelonioidea and Angolachelonia sister to a clade consisting of *Toxochelys latiremis* and the more crownward members of Pan-Chelonioidea in the 50% majority-rule consensus tree (electronic supplementary material, figure S18). However, the relationships within Pan-Chelonioidea are somewhat less resolved, with the Cenozoic stem cheloniids *Puppigerus*, *Eochelone* and *Argillochelys* forming a large polytomy. *Peritresius martini* is recovered as a sister taxon to *Allopleuron hofmanni* on the stem of Dermochelyidae and the clade formed by *Archelon* and *Protostega* is separated from the other members of Protostegidae. Support values are relatively high, with more than 60% support for almost all resolved nodes.

**Figure 3.**
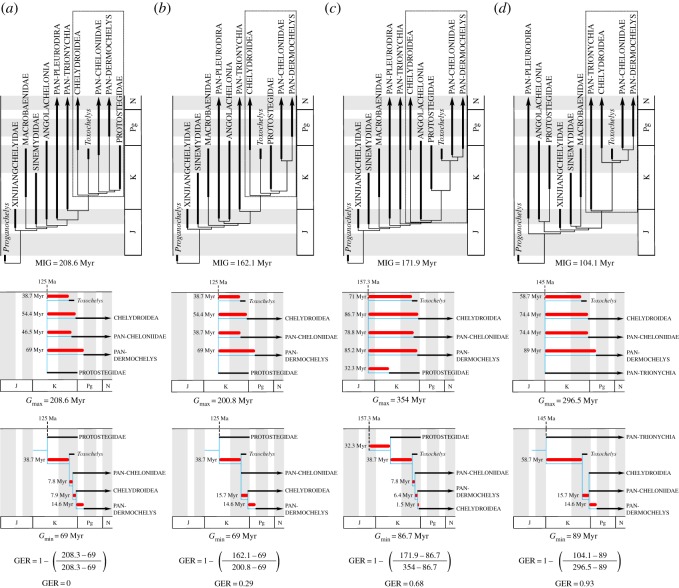
Stratigraphic fit of competing hypotheses for the arrangement of americhelydian lineages. (*a*) GER calculations for the topology recovered by Evers & Benson [9]. The fossil occurrence of *Eosphargis breineri* is used as the maximum age constraint for Pan-Dermochelys. (*b*) GER calculations for the topology recovered by Evers *et al*. [16]. The fossil occurrence of *Eosphargis breineri* is used as the maximum age constraint for Pan-Dermochelys and the occurrence of *Ctenochelys stenoporus* is used as the maximum age constraint for Pan-Cheloniidae. (*c*) GER calculations for the topology recovered in the weighted parsimony analysis. The fossil occurrence of *Allopleuron hofmanni* is used as the maximum age constraint for Pan-Dermochelys and *Euclastes weilandi* is used as the maximum age constraint for Pan-Cheloniidae. (*d*) GER calculations for the topology recovered by Joyce [14]. The fossil occurrence of *Eosphargis breineri* is used as the maximum age constraint for Pan-Dermochelys. Dotted lines, Americhelydia; MIG, minimum implied gap; *G*_max_, maximum gap; *G*_min_, minimum gap; blue lines represent hypothetical topologies derived solely from fossil occurrence data. See the electronic supplementary material for sources of fossil occurrence data.

Gap excess ratio (GER) values [48] were calculated to assess the stratigraphic congruence of americhelyidan lineages within our preferred topology (figure 3*c*) relative to that of the marine turtle phylogenetic hypothesis of Evers & Benson [9] (figure 3*a*), Evers *et al*. [16] (figure 3*b*), and Joyce [14] (figure 3*d*). To calculate GER, we first subtracted the theoretical minimum sum of the ghost lineages in a phylogeny purely based on stratigraphic occurrence (minimum gap = *G*_min_) from the implied ghost lineages in an actual topology (minimum implied gap = MIG). We then subtracted *G*_min_ from the summed differences between the origination time of the oldest included taxon and every other included taxon (maximum gap = *G*_max_). The first value was divided by the second and a ratio was created by subtracting the resulting value from 1. A GER of 1 indicates the best possible fit of topology and stratigraphic occurrence while a GER of 0 is indicative of the least congruent scenario (=oldest taxon highly nested within the phylogeny). Our analysis recovered a high GER score despite the odd recovery of the Late Jurassic thalassochelydians within Pan-Chelonioidea and the resulting increase in the implied ghost lineages for Chelydroidea and Protostegidae. This result is primarily due to the exclusion of Protostegidae from crown group Chelonioidea and the consequent reduction in the implied ghost lineages for both Pan-Cheloniidae and Pan-Dermochelys. The position of *Allopleuron* in our analysis supports the findings of Rabi & Kear [49] and further reduces the implied ghost lineage for Pan-Dermochelys by replacing *Eosphargis* as the earliest stem dermochelyid. Of all the hypotheses examined, the one that resulted in the highest stratigraphic congruence was that of Joyce [14], in which protostegids are removed from the Cryptodira crown group. It should be noted, however, that our examination of stratigraphic congruence was limited to only americhelydian lineages and that a more global approach might yield different results.

## 6. Discussion

**Figure 4.**
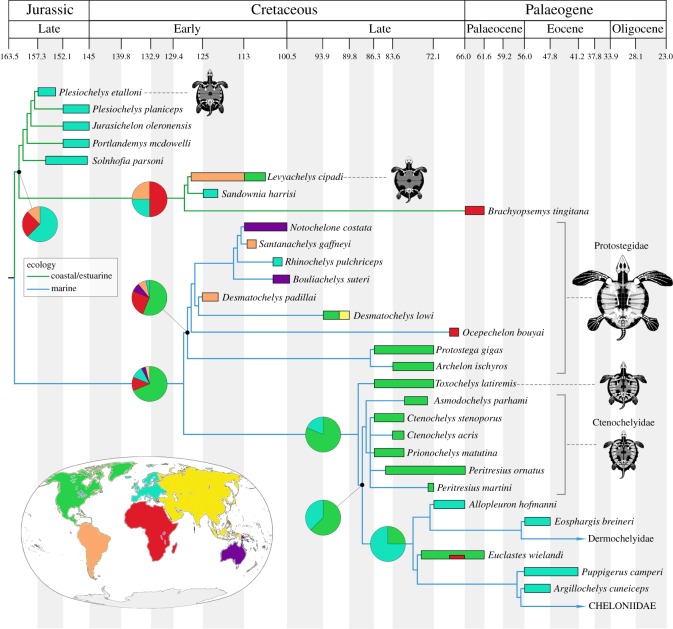
Age calibrated phylogeny of Pan-Chelonioidea with biogeographic occurrence of each species. Pie-charts represent ancestral range probabilities. See electronic supplementary material for sources of fossil occurrence data and a full list of ancestral range probability calculations.

Our phylogenetic analyses provide strong support for the placement of angolachelonians, protostegids and ctenochelyids as stem chelonioids. The placement of protostegids as stem chelonioids supports the conclusions of the most recent phylogenetic analyses of chelonioids [15,16] and is more congruent with the fossil record than other recent hypotheses of marine turtle evolution while still supporting the proposed singular origin of a pelagic ecology among non-pleurodiran turtles [9]. As almost all known Late Cretaceous non-protostegid chelonioids are North American, the exclusion of protostegids from crown group Chelonioidea resolves the biogeographic issues associated with the placement of protostegids as derived dermochelyoids (figure 4). Furthermore, the diversification of non-protostegid sea turtles during the Campanian took place following the extinction of most species of protostegid [37]. This scenario supports the previously hypothesized pattern of ecological replacement following the extinction of similarly adapted forms within the Pan-Chelonioidea lineage [50]. Our analyses indicate that the youngest stem chelonioids are probably North American taxa (62.5% probability), which partially supports the hypothetical biogeographic origin of crown chelonioids proposed by both fossil [5,11] and molecular [12] studies of turtles. However, we calculate a 75% probability of a European ancestral area for crown Chelonioidea. Despite this, the sister taxa relationship between *Allopleuron* and *Peritresius martini* suggested by our Bayesian analysis means that we cannot rule out the possibility that certain Late Cretaceous ctenochelyids from North America may be early stem dermochelyids. Since these inferences are based entirely on the topology of the strict consensus tree and sampled taxa, it is likely that the inclusion of additional Late Cretaceous and Palaeogene marine turtles in future studies will dramatically alter interpretations of the ancestral areas of crown Chelonioidea lineages.

## Supplementary Material

Supplementary Material

Reviewer comments

## Supplementary Material

Character-taxon matrix in NEXUS file format

## Data accessibility

A detailed geological description of the Demopolis Chalk, locality information for each *Asmodochelys* specimen, a complete list of the characters used in the phylogenetic analysis, the character matrix in NEXUS file format, the molecular constraint tree, the consensus trees of both the unweighted parsimony and Bayesian analyses as well as a full list of ancestral range probability calculations are provided as electronic supplementary material.
