# Supplementary material for: Asmodochelys parhami, a new fossil marine turtle from the Campanian Demopolis Chalk and the stratigraphic congruence of competing marine turtle phylogenies
Source: R Soc Open Sci. 2019 Dec 18;6(12):191950. doi: 10.1098/rsos.191950 (PMC6936288; doi:10.1098/rsos.191950)
Supplement: Supplementary Material [file rsos191950supp1.docx]

Electronic Supplementary Materials

for

***Asmodochelys parhami,* a new fossil marine turtle from the Campanian Demopolis Chalk and the stratigraphic congruence of competing marine turtle phylogenies**

Andrew D. Gentry^*1,2^, Jun A. Ebersole^2^, Cailtin R. Kiernan^2^

*^1^Department of Biology, University of Alabama at Birmingham, Birmingham, AL 35233, USA. gentryd@uab.edu*

*^2^Deparment of Collections, McWane Science Center, Birmingham, AL 35203, USA.*

Royal Society Open Science

DOI: http://dx.doi.org/10.1098/rsos.191950

**This file includes:** pp.

1. Materials and methods

1.1 Institutional abbreviations 3

1.2 Geology of the Demopolis Chalk 3

1.3 Locality information 5

1.4 Sources for phylogenetic scores 7

1.5 Sources for fossil occurrence data in figure 3 8

2. Description

2.1 Additional figures of *Asmodochelys parhami* material 10

2.2 Holotype measurements 15

3. Phylogenetic analysis

3.1 Character list 16

3.2 Molecular constraint 53

3.3 Ordered characters list 53

3.4 Unweighted parsimony strict consensus 54

3.5 Bayesian consensus 55

4. Probability calculations of ancestral areas for nodes within Pan-Chelonioidea

4.1 Probability list 56

4.2 Resolved node numbering 62

5. References

**1. Materials and methods**

**1.1 Institutional abbreviations**

**AMNH** – American Museum of Natural History, New York, NY, USA; **FMNH** – Field Museum of Natural History, Chicago, IL, USA; **IRScNB** – Institut Royal des Sciences Naturelles de Belgique, Brussels, BE; **KUVP** – University of Kansas, Lawrence, KS, USA; **MSC** – McWane Science Center, Birmingham, AL, USA; **MMNS** – Mississippi Museum of Natural Sciences, Jackson, MS, USA; **NHMUK** – Natural History Museum, London, UK; **NJSM** – New Jersey State Museum, Trenton, NJ, USA; **ROM** – Royal Ontario Museum, Toronto, ON, CA; **SMNS** – Staatliches Museum für Naturkunde, Stuttgart, Germany; **USNM** – United States National Museum, Washington, D.C., USA; **YPM** – Yale Peabody Museum, New Haven, CT, USA.

**1.2 Geology of the Demopolis Chalk**

In Alabama and Mississippi, the Demopolis Chalk is part of the Campanian and Maastrichtian Selma Group, a series of stratigraphic units that includes, in ascending order, the Blufftown Formation, Mooreville Chalk, Demopolis Chalk, Ripley Formation, Prairie Bluff Chalk, and Providence Sand. Surface exposures of the Upper Campanian Demopolis Chalk form a narrow belt that extends across central Alabama and into northeast Mississippi. Surface outcrops of this unit have been mapped in 12 counties in central Alabama and 13 counties in Mississippi (Fig. S1). Puckett (2005) has recognized three distinct subdivisions within the Demopolis Chalk in northeastern Mississippi: the lower Tibbee Creek Member, middle "Muldrow" Member (considered here to be an informal member), and upper Bluffport Marl Member (Fig. S2). In western and central Alabama, the Tibbee Creek Member is entirely absent from the section and the "Muldrow" Member rests conformably on top of the Arcola Limestone Member of the Mooreville Chalk. In eastern Alabama and western Georgia, the Demopolis Chalk is replaced by the coeval Cusseta Sand Member of the Ripley Formation. In both Mississippi and Alabama, the uppermost Demopolis Chalk is overlain by the Ripley Formation.

The Demopolis Chalk represents a portion of the second of three chalk and marl transgressive-regressive cycles present in the north Gulf Coastal Plain (Mancini and Puck­ett, 2005), proceeding upwards from the gray fossiliferous sandy marls of the Tibbee Creek Member (in Mississippi), to the light gray to white chalks of the "Muldrow Member," to the grey fossiliferous silty, sandy marls of the Bluffport Marl Member. The "Muldrow” Member chalks are the purest found within the Selma Group (72%-90% CaCO_3_; Dockery and Thompson, 2016). The "Muldrow" was deposited in an outer neritic environment, with the Bluffport originating under shallower middle neritic conditions and therefore receiving a greater influx of clastic material (Puckett, 1992).

The holotype and paratypes of *Asmodochelys parhami* gen. et sp. nov were collected from exposures of the "Muldrow" and Bluffport Marl members. Both units have been dated as Upper Campanian, based on calcareous nanoplankton, with the "Muldrow" Member extending from the upper half of NP Zone CC20 to the lower part of NP Zone CC23, while the Bluffport Marl Member ­occurs entirely within NP Zone CC23 (Russell et al. 1983). This conclusion is supported by Puckett (2005), who employs high-resolution ostracode and foraminfieral biostratigraphy to date both these members of the Demopolis Chalk as Upper Campanian.

**1.3 Locality information**

**
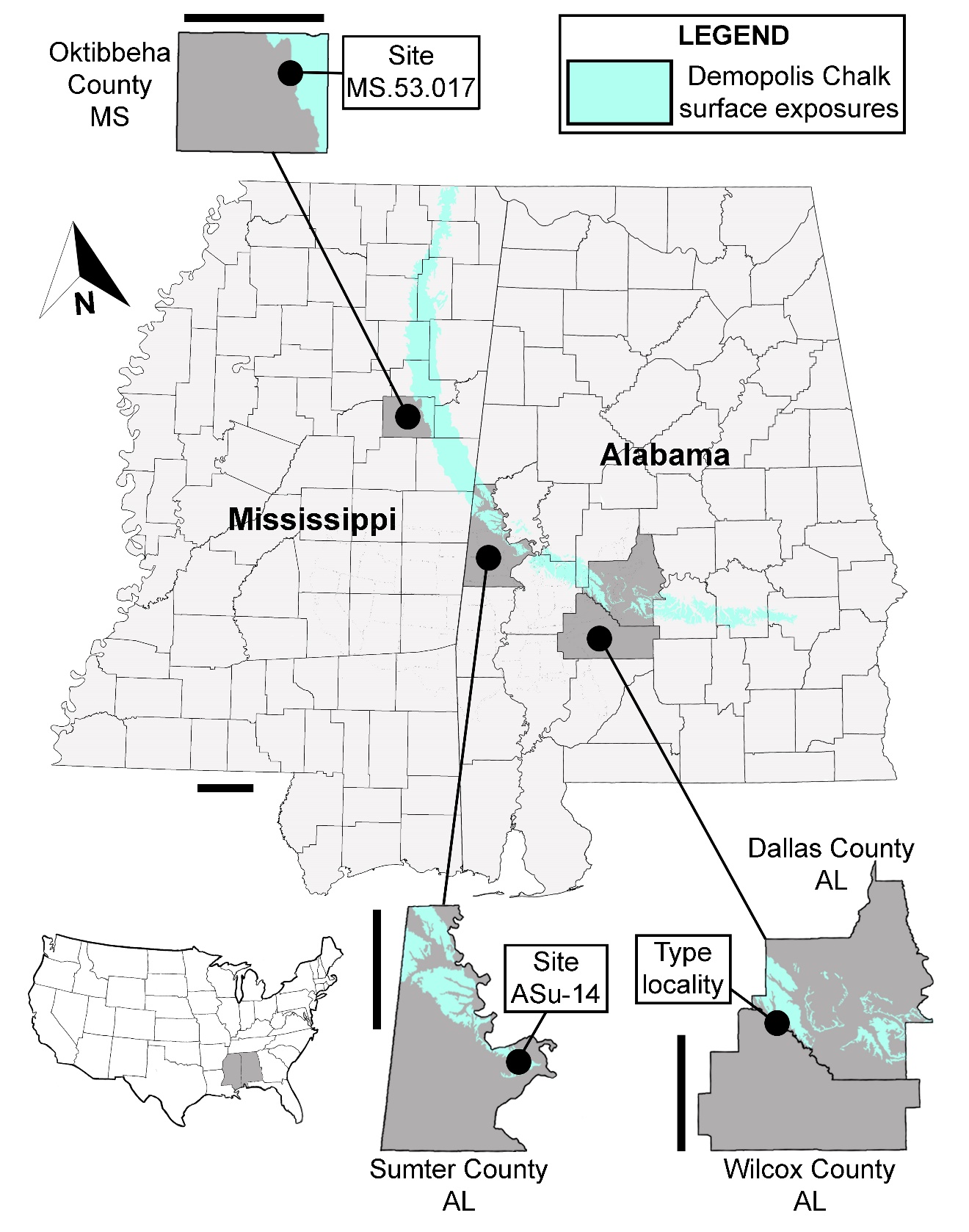
**

**Fig. S2.** Collecting localities for specimens examined in this study. Blue areas represent surface exposures of the upper Campanian Demopolis Chalk. Scale bars = 30 km.


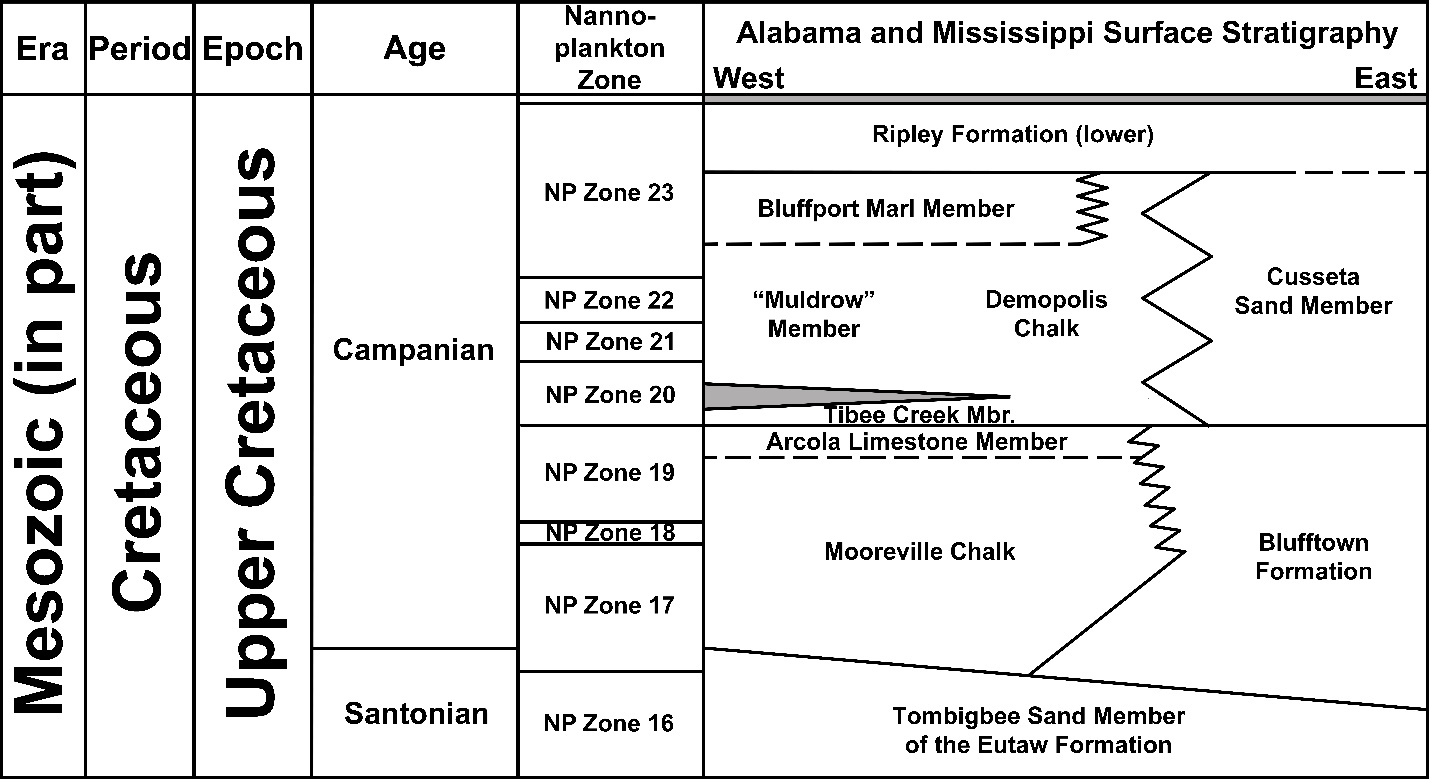


**Fig. S1.** Santonian through Campanian surface stratigraphy for Alabama and Mississippi, USA. Gray areas represent unconformities.

**Specimen Number:** MSC 35984, holotype.

**Type Locality:** Town of Alberta, Wilcox County, Alabama, USA, GPS coordinates: 32.233029, -87.409997.

**Type stratum:** “Muldrow” Member of the Demopolis Chalk.

**Stratigraphic Age:** Upper Campanian, NP Zone CC22c.

**Remarks:** The holotype specimen, MSC 35984, was historically collected from Upper Cretaceous surface exposures in the 1985. Notes associated with the specimen indicate it was collected within the town limits of Alberta in Wilcox County, Alabama, USA. Because no additional locality information accompanied the specimen, two of the present authors (JAE and ADG) visited the town of Alberta in the winter of 2019 to examine any Upper Cretaceous surface exposures in the vicinity. The town center of Alberta is small with only five named roads, the largest of which, Alabama State Highway 5, runs north to south through the center of the town. All road side exposures within the city limits were examined, however all consisted of recent alluvium. Less than a half kilometer to the north of the center of Alberta, Highway 5 is intersected by the east to west flowing Chilatchee Creek, which marks the boundary of Wilcox and Dallas counties. Although geological exposures were observed on the banks of the creek, water levels were high at the time of visitation and only a recent sandy alluvium was exposed. It is our opinion, however, that the type specimen was likely collected from Chilatchee Creek as it is easily accessible, and the width and depth of the creek strongly suggests it cuts through the underlying Upper Cretaceous units that have been mapped in the area. No additional vertebrate specimens are known to have been collected from this locality.

Because no Upper Cretaceous surface exposures could be directly observed in the Alberta area, the type stratum was determined by removing matrix from the holotype specimen and examined for the presence of calcareous nannoplanktons by personnel at the United States Geological Survey in Reston, VA, USA. The subsequent analysis showed the co-occurrence of the nannoplanktons *Reinhardtites levis* and *Reinhardtites anthophorous*, and the presence of background species such as *Arkhangelskiella cymbiformis, Aspidolithus parcus constrictus*, and *Tranolithus phacelosus*. The combination of these calcareous nannoplanktons places the examined matrix sample within NP Zone CC22c, in turn showing that MSC 35984 was derived from the upper Campanian “Muldrow” Member of the Demopolis Chalk.

**Specimen Number:** MSC 40935, paratype.

**Locality:** Site ASu-14, Sumter County, Alabama, USA. More precise locality data is available to qualified researchers and is archived at MSC.

**Stratum:** Bluffport Marl Member of the Demopolis Chalk.

**Stratigraphic Age:** Upper Campanian, lower half of NP Zone CC23.

**Remarks:** Specimen MSC 40935 was collected by one of the present authors (CRK) during two separate collecting trips to site ASu-14 in November 2001 and April 2002. Site ASu-14 is located on private land and consists of a small series of erosional chalk gullies. This locality exposes the contact of the “Muldrow” and Bluffport Marl members of the Demopolis Chalk, with the specimen being derived three to four meters above this contact within the more marly Bluffport Marl Member. Biostomes of *Exogyra costata, Pynodonte mutabilis, Agerostrea falcata* occur at this locality which are known to mark the base of the Bluffport Marl Member (Russell et al., 1983). The stratigraphic position of specimen MSC 40935 suggests it falls within the lower half of NP Zone CC23 and is Upper Campanian in age. Vertebrate taxa recovered in conjunction with specimen MSC 40935 include *Cretalamna* sp., *Enchodus* *petrosus*, Hadrosauridae indet.,

*Ischyrhiza mira*, *Mosasaurus* spp., Ornithurae indet., *Pachyrhizodus* caninus, *Plioplatecarpus* sp., *?Prognathodon* sp., *Squalicorax kaupi*, *S. pristodontus*, and ?*Toxochelys moorevillensis*.

**Specimen Number:** MMNS 3985, paratype.

**Locality:** Site MS.53.017, Rockhill Road, Starkville, Oktibbeha County, MS, USA. More precise locality data is available to qualified researchers and is archived at the MMNS.

**Stratum:** “Muldrow” Member of the Demopolis Chalk.

**Stratigraphic Age:** Upper Campanian, lower part of NP Zone CC23.

**Remarks:** MMNS 3985 was collected on April 17, 2005 from the Rock Hill Road locality in northeast Starkville, MS. This series of erosional gullies exposes the contact of the “Muldrow” and Bluffport Marl members of the Demopolis Chalk (see Puckett, 2005), with the specimens being derived from the “Muldrow” Member (George Phillips, Pers. Com., 2019). Puckett (2005) examined the ostracode and foraminiferal biostratigraphy within this area by showed the exposed portion of the “Muldrow” Member at the Rock Hill locality fell within the *Escharachthridea pinochii* Ostrocode Zone and the *Globotruncanella havanensis* Planktonic Foraminiferal Zone, placing the specimen within the upper 11 meters of member (see Puckett (2005) samples RH 214, 220, 230, 235, 245, 250). This indicates that the age of specimen MMNS 3985 resides within the Upper Campanian, and stratigraphically likely falls within the lower part of NP Zone CC23. Vertebrates collected in association with MMNS 3985 include *Squalicorax pristodontus*, *Encodus* spp., Teleostei indet., and mosasauridae indet.

**1.4 Sources for phylogenetic scores**

| Species | Specimens & Sources |
| --- | --- |
| Asmodochelys parhami gen. et sp. nov.* | **MSC 35984 (holotype)** **MMNS 3958 (paratype) MSC 40935 (paratype)** |
| Toxochelys latiremis* | **AMNH 2362 (holotype)** (Zangerl, 1953) **YPM 3602** (Zangerl, 1953b) **AMNH 5118** (Matzke, 2009) **AMNH 1042** (Maztke, 2009) **USNM 11560** (Matzke, 2009) **ROM 28563** (Nicholls, 1988) **FMNH PR123** (Zangerl, 1953b; pers. obs.) |
| Ctenochelys stenoporus* | **KUVP 1205 (holotype)** (Hay, 1908; Zangerl, 1953b) **AMNH 6137** (Hay, 1908 = *Toxochelys elkader*; Zangerl, 1953 = *Ctenochelys stenopora*) **USNM 357166**, **USNM 391920** (Matzke, 2007) |
| Ctenochelys acris* | **FMNH P27354 (holotype)** (Zangerl, 1953b; pers. obs.) **MSC 6157**, **MSC 35085** (Gentry, 2016; pers. obs.) |
| Prionochelys matutina* | **FMNH P27561 (holotype)** (Zangerl, 1953; Gentry, 2018; pers. obs.) **MSC 39030**, **MSC 3036** (Gentry, 2018; pers. obs.) |
| Peritresius ornatus* | **NJSM 11051 (holotype)** (Baird, 1964) |
| Allopleuron hofmanni | **IRScNB EFR 8**, **IRScNB 3668** (Mulder, 2003) |
| Euclastes wielandi | **YPM VP 000625 (paratype)** Hay, 1908 **AMNH 30022** (Hirayama & Tong, 2003) **NJSM 11872** (Fastovsky, 1985) |
| Protostega gigas (= Protostega dixie) | **FMNH P27353** (Zangerl, 1953a; pers. obs.) **FMNH P27315** (Zangerl, 1953a; pers. obs.) |

**Table S1.** Fossil species added to the present matrix not included in Evers & Benson, 2018 and the associated specimens and sources used for the phylogenetic scores.

**1.5 Sources of fossil occurrence data**

| **Species** | **Temporal range (Mya)** | **Continental occurrence** | **Sources** |
| --- | --- | --- | --- |
|  |  |  |  |
| *Levyachelys cipadi* | 127.0 - 105.0 | North America & South America | Cadena, 2015 |
| *Sandownia harrisi* | 125.0 - 122.0 | Europe | Meylan et al., 2000 |
| *Brachyopsemys tingitana* | 66.0 - 61.6 | Africa | Tong & Meylan, 2013 |
| *Plesiochelys etalloni* | 157.3 - 150.0 | Europe | Anquetin et al., 2017 |
| *Plesiochelys planiceps* | 152.1 - 145.0 | Europe | Anquetin et al., 2017 |
| *Jurassichelon oleronensis* | 152.1 - 145.0 | Europe | Anquetin et al., 2017 |
| *Portlandemys mcdowelli* | 152.1 - 145.0 | Europe | Anquetin et al., 2017 |
| *Solnhofia parsoni* | 155.7 - 145.5 | Europe | Evers & Benson, 2018; Anquetin et al., 2017 |
| *Archelon ischyros* | 83.6 - 72.1 | North America | Hirayama, 1997 |
| *Protostega gigas* | 86.3 - 72.1 | North America | Zangerl, 1953a; Hirayama, 1997 |
| *Ocepechelon bouyai* | 69.0 - 67.6 | Africa | Bardet et al., 2013 |
| *Desmatochelys padillai* | 125 – 120 | South America | Cadena & Parham, 2015 |
| *Desmatcohelys lowi* | 93.9 - 89.8 | North America & Asia | Hirayama, 1997; Sato et al., 2012 |
| *Bouliachelys suteri* | 105.3 - 99.7 | Australia | Evers & Benson, 2018; Kear & Lee, 2006 |
| *Santanachelys gaffneyi* | 112.6 - 109.0 | South America | Evers & Benson, 2018; Hirayama, 1998 |
| *Rhinochelys pulchriceps* | 105.3 - 102.0 | Europe | Evers & Benson, 2018; Collins, 1970 |
| *Notochelone costata* | 113.0 - 100.5 | Australia | Gaffney, 1981 |
| *Toxochelys latiremis* | 86.3 - 72.1 | North America | Nicholls, 1988; Kauffman et al., 1993; Gentry et al., 2018 |
| *Asmodochelys parhami* | 80.5 - 73.8 | North America | this paper |
| *Prionochelys matutina* | 86.3 - 80.5 | North America | Gentry, 2018 |
| *Ctenochelys stenoporus* | 86.3 - 80.5 | North America | Zangerl, 1953b; Matzke, 2007 |
| *Ctenochelys acris* | 83.6 - 80.5 | North America | Zangerl, 1953b; Gentry, 2016 |
| *Peritresius ornatus* | 83.6 - 66.0 | North America | Baird, 1964; Gentry et al., 2018 |
| *Peritresius martini* | 74.0 - 72.1 | North America | Gentry et al., 2018 |
| *Allopleuron hofmanni* | 72.1 - 66.0 | Europe | Mulder, 2003; Jansen et al., 2011 |
| *Eosphargis breineri* | 56.0 - 47.8 | Europe | Nielsen, 1959 |
| *Puppigerus camperi* | 56.0 - 41.2 | Europe | Weems & Brown, 2017 |
| *Argillochelys cuneiceps* | 56.0 - 47.8 | Europe | Weems & Brown, 2017 |
| *Euclastes wielandi* | 78.5 - 61.6 | North America & Africa | Schwimmer et al., 2015 |

**Table S2.** Sources of temporal and biogeographic occurrence data used in figure 3.

**2. Description**

**2.1 Additional figures of *Asmodochelys parhami* material**

**
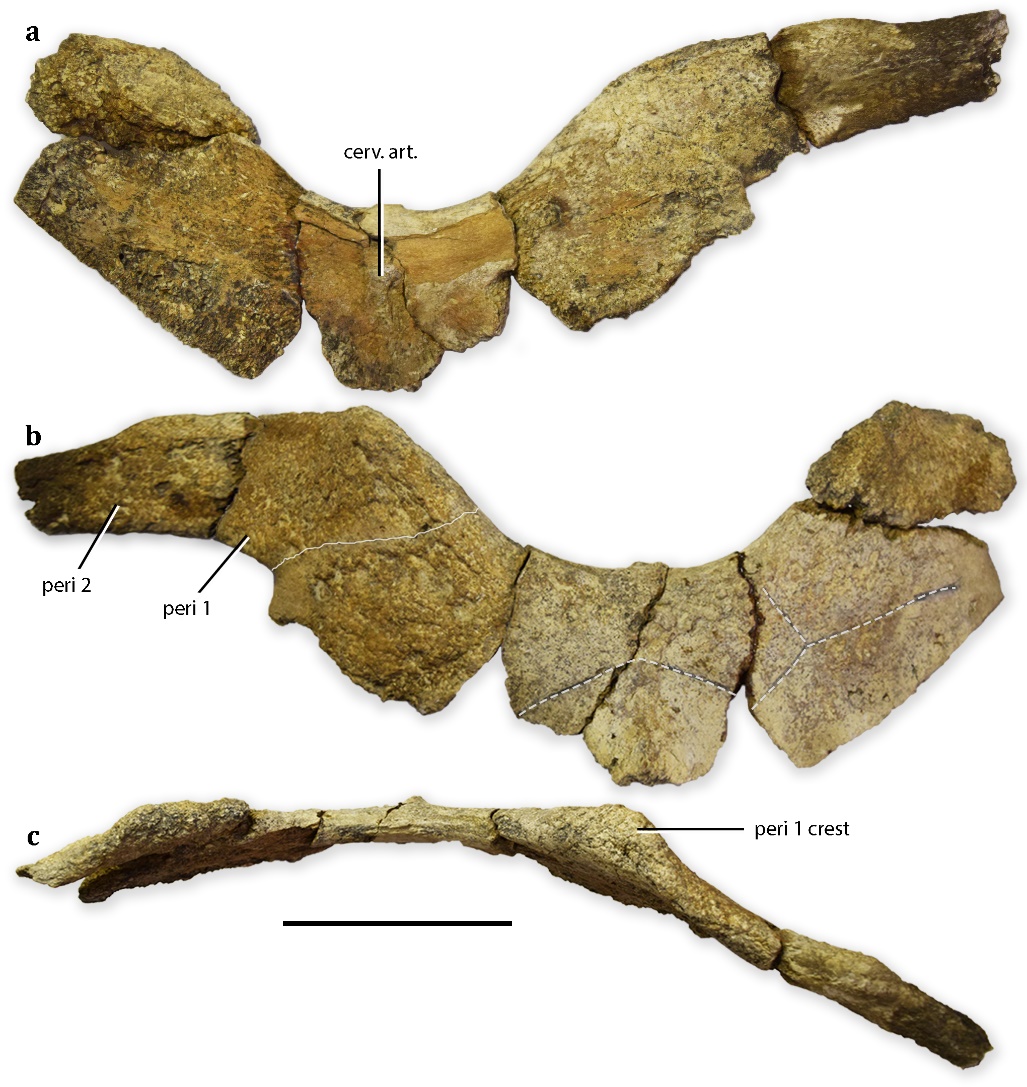
**

**Fig. S3.** *Asmodochelys parhami* (MSC35984) - Nuchal, first peripherals, and second left peripheral in (**a**) ventral, (**b**) dorsal, and (**c**) anterior views. Scale bar = 10 cm.


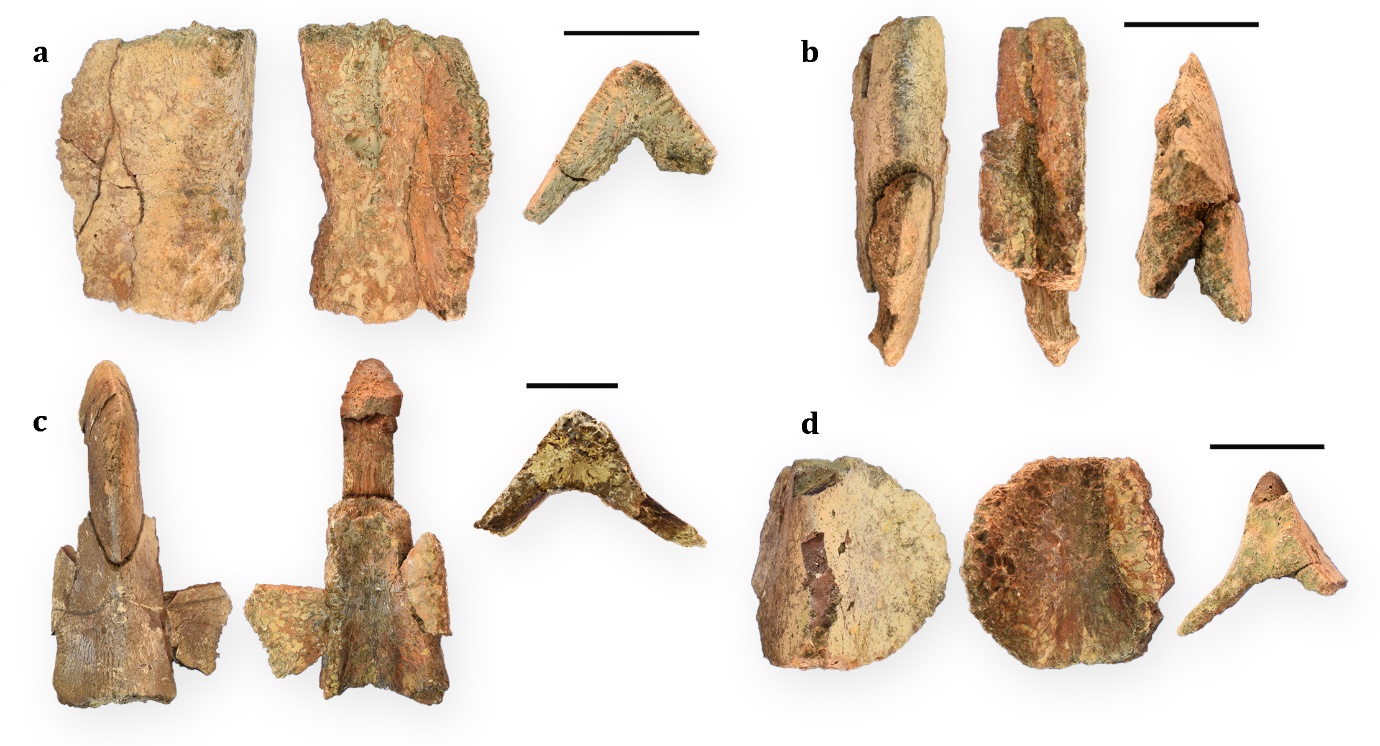


**Fig. S4.** *Asmodochelys parhami* (MSC35984) - Neurals and epineurals (**a-d**) in (left) dorsal, (middle) ventral, and (right) anterior views. Scale bars = 5 cm.

**
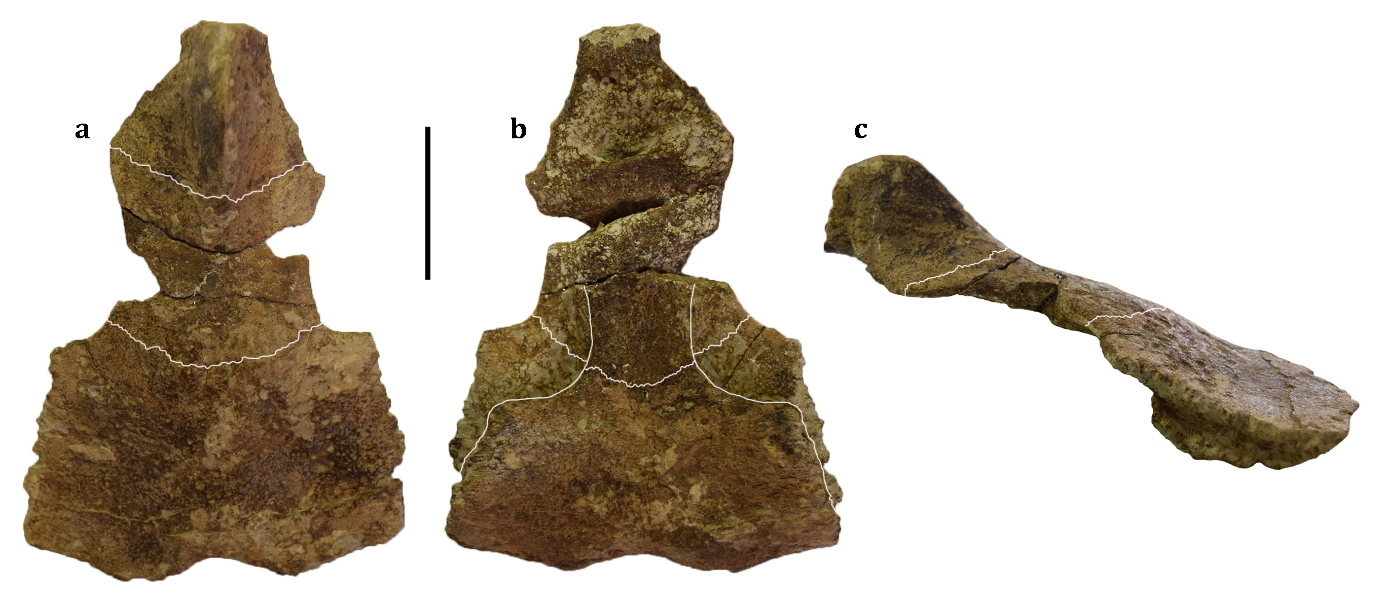
**

**Fig. S5.** *Asmodochelys parhami* (MMNS 3958) - Pygal and suprapygals (**a-c**) in (**a**) dorsal, (**b**) ventral, and (**c**) left lateral views. Scale bar = 10 cm.


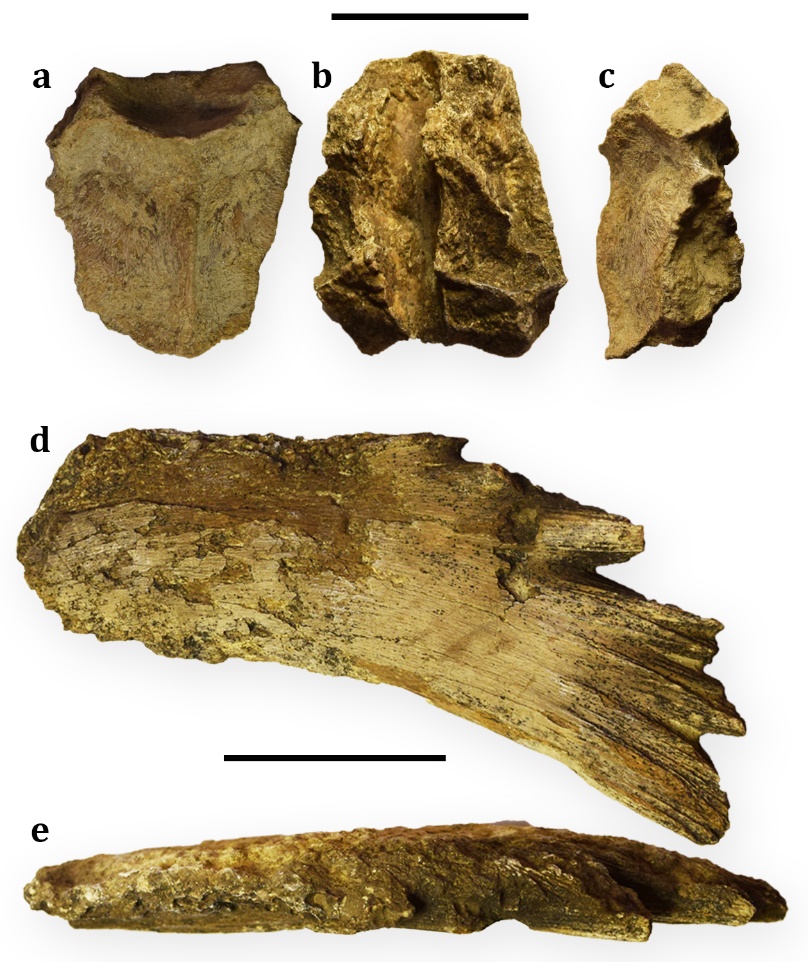


**Fig. S6.** *Asmodochelys parhami* (MSC35984) - Cervical vertebra in (**a**) ventral, (**b**) dorsal, and (**c**) left lateral views. Scale bar = 3 cm. Plastral fragment in (**d**) ventral and (**e**) posterior views. Scale bar = 10 cm.

**
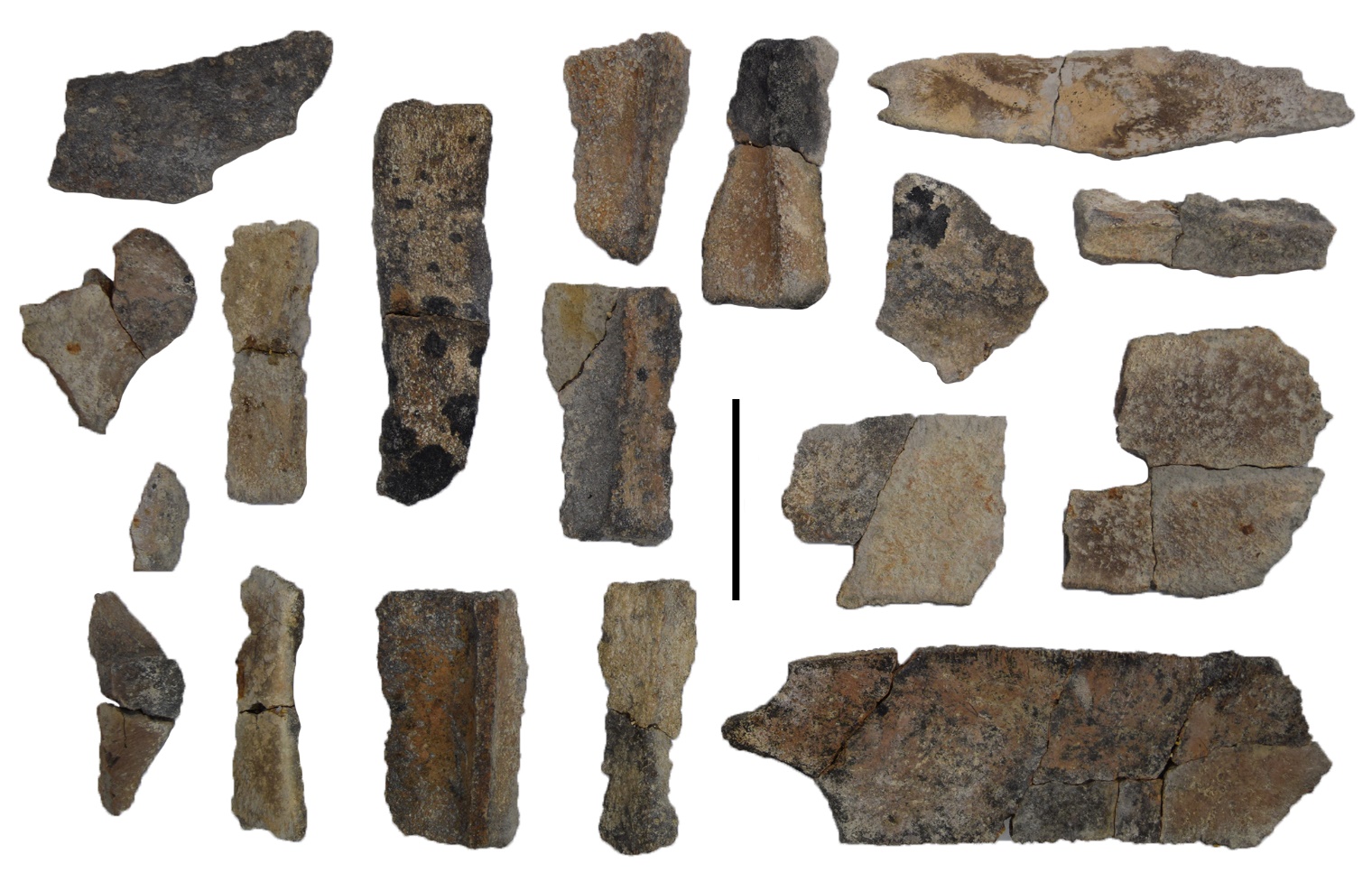
**

**Fig. S7.** *Asmodochelys parhami* (MSC 40935) – Carapacial and plastral fragments. Scale bar = 10 cm.

**
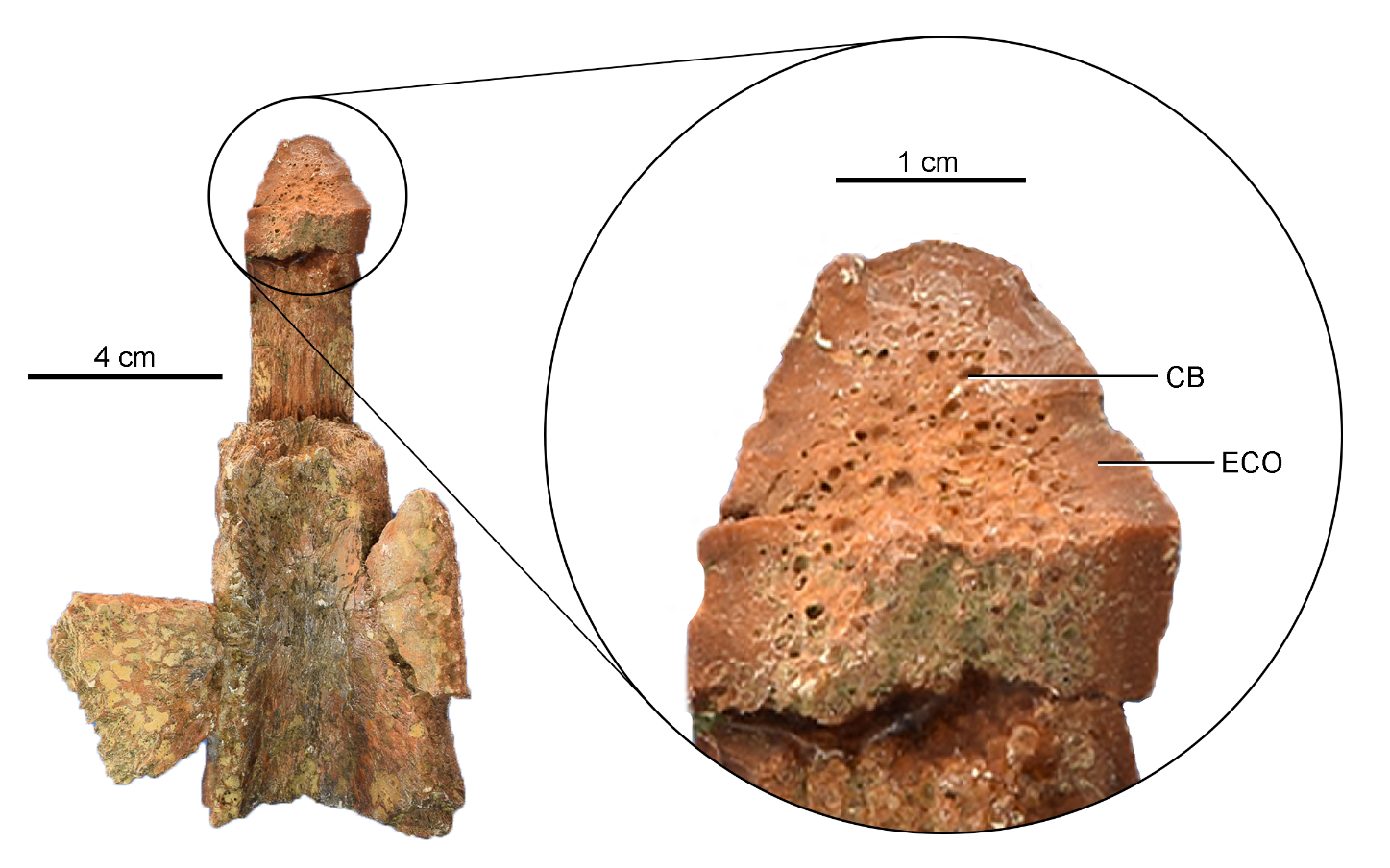
**

**Fig. S8.** *Asmodochelys parhami* (MSC 35984) sixth neural and third epineural in ventral view. Abbreviations: CB, cancellous bone; ECO, external cortex.

**2.2 Holotype measurements**

**Table S3.** *Asmodochelys parhami* (MSC 35984) holotype. All measurements in mm. * = estimates.

**3. Phylogenetic analysis**

**3.1 Character List**

Unless otherwise specified, the character list presented here is largely unchanged from that of Evers & Benson (2018). For detailed character explanations, histories, and previous definitions, see Evers & Benson (2018; Appendix S1). Scoring adjustments to the character-taxon matrix of Evers & Benson (2018) are listed under “*Remarks”* below the corresponding character.

**CRANIUM**

**Character 1.** Nasals: 0 = present; 1 = absent.

**Character 2.** Nasal, medial contact of nasals: 0 = nasals contact one another medially along their entire length; 1 = medial contact of nasals reduced or absent.

*Remarks*: Evers and Benson (2018) score *Toxochelys* sp. as having an extensive medial contact between the nasals (ch. 2.0). The most complete skull of *Toxochelys latiremis* currently known (AMNH 5118) exhibits highly reduced nasals along the dorsolateral margins of the apertura narium externa which do not possess any medial contact (Matzke, 2009; Text-fig. 2, p. 101). We score *Toxochelys latiremis* as state 1.

**Character 3.** Nasal, size of nasals: 0 = dorsal exposure of nasals large; 1 = dorsal exposure of nasals greatly reduced relative to that of the frontals.

**Character 4.** Prefrontals, medial contact of prefrontals on the dorsal skull surface: 0 = absent; 1 = present, absence of contact between the nasal or apertura narium externa and the frontal.

**Character 5.** Prefrontal, prefrontal-vomer contact: 0 = present; 1 = absent.

**Character 6.** Prefrontal, prefrontal-palatine contact: 0 = present; 1 = absent.

**Character 7.** Prefrontal, dorsal prefrontal exposure: 0 = present, large; 1 = reduced; 2 = absent or near absent.

**Character 8.** Prefrontal, cranial scutes on the prefrontal: 0 = one pair; 1= two pairs or more.

**Character 9.** Prefrontal, sculpturing: 0 = heavily sculptured; 1 = sculpturing absent.

**Character 10.** Lacrimal: 0 = present; 1 = absent.

**Character 11.** Frontal, frontal contribution to orbit: 0 = absent, contact between prefrontal and postorbital; 1 = present.

**Character 12**. Frontals, both frontals medially fused: 0 = absent; 1 = present.

**Character 13.** Frontal, direction of the orbits in dorsal view of the skull: 0 = laterally facing, with a very narrow to almost complete absent dorsal exposure of the maxilla and jugal; 1 = dorsolateral facing, with portions of the maxilla and jugal dorsally exposed.

**Character 14.** Frontals, development of crista cranii: 0 = crista cranii on ventral surface of frontals very shallow, sulcus olfactorius developed is a low trough; 1 = crista cranii developed as moderately deep parasagittal ridges on the ventral surface of each frontal, forming a ventrally open, median trough (the sulcus olfactorius) that extends from the anterior margin of the cavum cranii posteriorly to the fissure ethmoidalis anteriorly; 2 = crista cranii very deep anteriorly, forming extended processes that meet along the midline of the cranium and are sutured to one another, forming an ossified olfactory canal.

**Character 15.** Parietal, parietal-squamosal contact: 0 = present, upper temporal emargination absent or poorly developed; 1 = absent, upper temporal emargination well developed.

*Remarks*: Evers & Benson (2018) score *Toxochelys sp.* as lacking a contact between the parietal and squamosal (state 1). *Toxochelys latiremis* is known to possess a small contact between the parietal and squamosal (AMNH 5118 in Matzke, 2009; Text-fig. 2, p. 101) and the skull roof of *Toxochelys moorevillensis* is unknown. We score *Toxochelys latiremis* as state 0.

**Character 16.** Parietal, posterodorsal margin of the temporal fossa roofed by an overhanging process of the skull roof: 0 = absent; 1 = present.

**Character 17.** Parietal, contribution to the processus trochlearis oticum: 0 = absent; 1 = present.

**Character 18.** Parietals, foramen stapediotemporalis: 0 = absent or weak, foramen stapedio- temporale concealed in dorsal view; 1 = moderate foramen stapedio-temporale, partial exposition of the processes trochlearis in dorsal view; 2 = strong, entire exposition of the processus trochlearis in dorsal view.

*Remarks*: Evers and Benson (2018) score *Toxochelys sp.* as having a partial exposition of the processes trochlearis in dorsal view (ch. 18.1). *Toxochelys latiremis* possesses a poorly developed upper temporal emargination and lacks any dorsal exposure of the processes trochlearis or foramen stapedio temporale (AMNH 5118 in Matzke, 2009; Text-fig. 2, p. 101). We score *Toxochelys latiremis* as state 0.

**Character 19.** Parietal, pineal foramen located medially between parietals: 0 = absent; 1 = present.

**Character 20.** Parietal, processus inferior parietalis: 0 = weak or absent, parietal does not contact the pterygoid, epipterygoid, and/or palatine; 1 = present and well developed, the parietal contacts the pterygoid, epipterygoid, and/or palatine.

**Character 21.** Parietal, closure of foramen nervi trigemini and the length of the anterior extension of the lateral braincase wall: 0 = foramen nervi trigemini anteriorly open, anterior extension of lateral braincase wall absent; 1 = foramen nervi trigemini anteriorly closed, processus inferior parietalis only produces a narrow strut anterior to the foramen nervi trigemini, usually absence of contact with palatine; 2 = foramen nerivi trigemini anteriorly closed, processus inferior parietalis produces an extended process anterior to the foramen nervi trigemini, contact with palatine commonly present.

**Character 22.** Parietal, posterior ramus of processus inferior parietalis forming the posterior margin of the trigeminal foramen: 0 = absent; 1 = present.

*Remarks*: Evers & Benson (2018) score *Toxochelys sp.* as unknown for this character. In *Toxochelys latiremis*, the posterior margin of the foramen trigemini is formed by a contact between the prootic and epipterygoid (see Matzke, 2009; Text-fig. 9e, p. 111) and is scored here as state 0.

**Character 23.** Posterior ramus of processus inferior parietalis of the parietal: 0 = short; 1 = long, excludes the prootic from the trigeminal foramen. This character is scored inapplicable for taxa that lack the process altogether.

**Character 24.** Parietal, ridge on lateral surface of processus inferior parietalis: 0 = absent; 1 = present, a ridge between the ventral surface of the parietal and the lateral surface of the descending process marks the border between the temporal and orbital fossae.

**Character 25.** Jugal, jugal-squamosal contact: 0 = present; 1 = absent.

**Character 26.** Jugal, jugal participation in the margin of the upper temporal emargination: 0 = absent; 1 = present, upper temporal emargination extensive.

**Character 27.** Jugal, medial process of jugal ventral to orbit: 0 = weakly developed or absent, jugal contacts only the maxilla; 1 = present and well developed, jugal contacts the

maxilla as well as the palatine and/or pterygoid.

**Character 28.** Jugal, contact with the palatine: 0 = absent; 1 = present. This character is scored as inapplicable when the jugal lacks a medial process (ch 27.0).


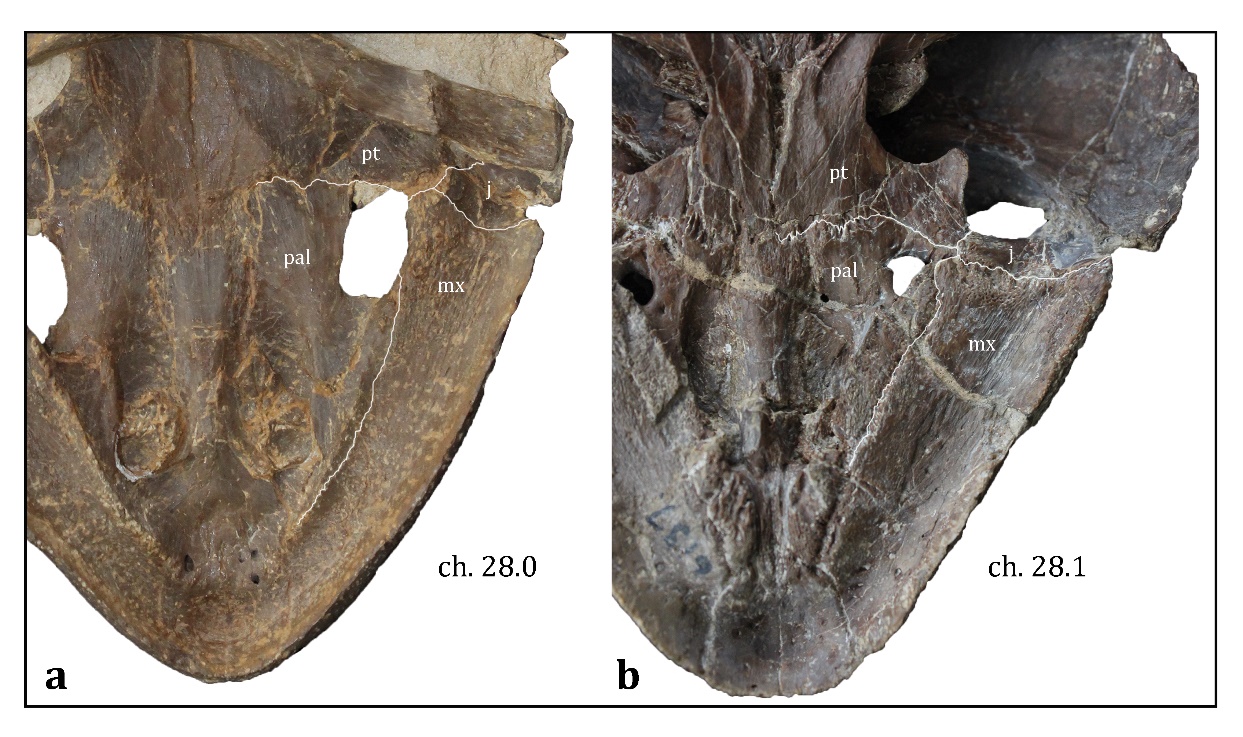


**Fig. S9.** *Toxochelys latiremis* (AMNH 5118) and *Ctenochelys stenoporus* (AMNH 6137) in ventral view illustrating the variation in medial process of the jugal present in Late Cretaceous non-protostegid stem chelonioids. Abbreviations: mx, maxilla; pal, palatine; pt, pterygoid.

**Character 29**. Jugal, contact with the pterygoid: 0 = absent; 1 = present. This character is scored as inapplicable when the jugal lacks a medial process (ch 27.0).

**Character 30.** Jugal, jugal-parietal contact: 0 = absent; 1 = present.

**Character 31.** Quadratojugal: 0 = present; 1 = absent.

**Character 32.** Quadratojugal, quadratojugal-maxilla contact: 0 = absent; 1 = present, jugal does not contribute to lower temporal emargination.

**Character 33.** Quadratojugal, quadratojugal-squamosal contact below the cavum tympani: 0 = absent; 1 = present.

**Character 34.** Quadratojugal, lower temporal emargination: 0 = weak to no emargination, the margin of the lower temporal emargination is formed by the quadratojugal or quadratojugal and jugal; 1 = moderate emargination, the margin of the lower temporal emargination is principally formed by the quadratojugal and jugal, but the maxilla is included in the anterior section of the margin and/or the quadrate is included in the posterior section of the margin; 2 = large emargination, the postorbital and/or squamosal and parietal are included in the margin of the lower temporal emargination.

*Remarks*: The posterolateral edge of the maxilla and the anterolateral edge of the quadrate both minimally contribute to the lower temporal emargination of *Toxochelys latiremis*: AMNH 5118 (Matzke, 2009, Text-fig. 3, p. 102). *Toxochelys latiremis* is scored here as state 1, contra Evers & Benson, 2018.

**Character 35.** Squamosal, squamosal/postorbital contact: 0 = present; 1 = absent.

**Character 36.** Squamosal, squamosal/supraoccipital contact: 0 = absent; 1 = present.

**Character 37.** Squamosal, posterolateral protuberances developing horns: 0 = absent; 1 = present.

**Character 38.** Squamosal, very long posterior process, formed exclusively by the squamosal and protruding beyond condyles occipitalis: 0 = absent; 1 = present.

**Character 39.** Squamosal, squamosal/quadrate contact: 0 = tightly sutured; 1 = wide open.

**Character 40.** Squamosal, posterodorsal margin of cavum tympanum: 0 = the squamosal forms the posterodorsal margin of the cavum tympanum; 1 = the squamosal is excluded from the posterodorsal margin of the cavum tympanum.

*Remarks*: Evers & Benson (2018) score *Toxochelys sp.* as unknown for this character. Although the posterodorsal portion of the quadrate is incompletely preserved in the most complete skull of *Toxochelys latiremis*: AMNH 5118 (Matzke, 2009; Text-fig. 2, p. 101), the entirety of the quadrate-squamosal contact appears to be well within the cavum tympanum and interior to the contribution of the squamosal to the posterodorsal margin of the cavum tympanum. *Toxochelys latiremis* is scored here as state 0 for this character.

**Character 41.** Postorbital, postorbital/palatine contact: 0 = absent; 1 = present, foramen palatinum posterius situated posterior to the orbital wall.

**Character 42.** Postorbital, contact with the quadratojugal: 0 = present; 1 = absent.

**Character 43.** Postorbital, postorbital-maxilla contact preventing the jugal from entering the orbital margin: 0 = absent; 1 = present.

**Character 44.** Postorbital, dorsal margin of orbit: 0 = continuously and concavely curved margin between frontals and jugal; 1 = frontal margin relatively narrow, with lateral bulge of postorbital.

*Remarks*: Evers & Benson (2018) note the presence of this feature in certain total-group cheloniids (e.g. *Allopleuron hofmanni*: NHMUK R 42931; *Argillochelys cuneiceps*: NHMUK 38955; *Lepidochelys olivacea*: SMNS 11070). We also observed this condition in *Ctenochelys acris*: MSC 6157 (Gentry, 2016; Fig. 4, p. 6) and *Ctenochelys stenoporus*: USNM 357166 (Matzke, 2007; Text-fig. 1, p. 672) and AMNH 6137 (Zangerl, 1953b; pl. 17).


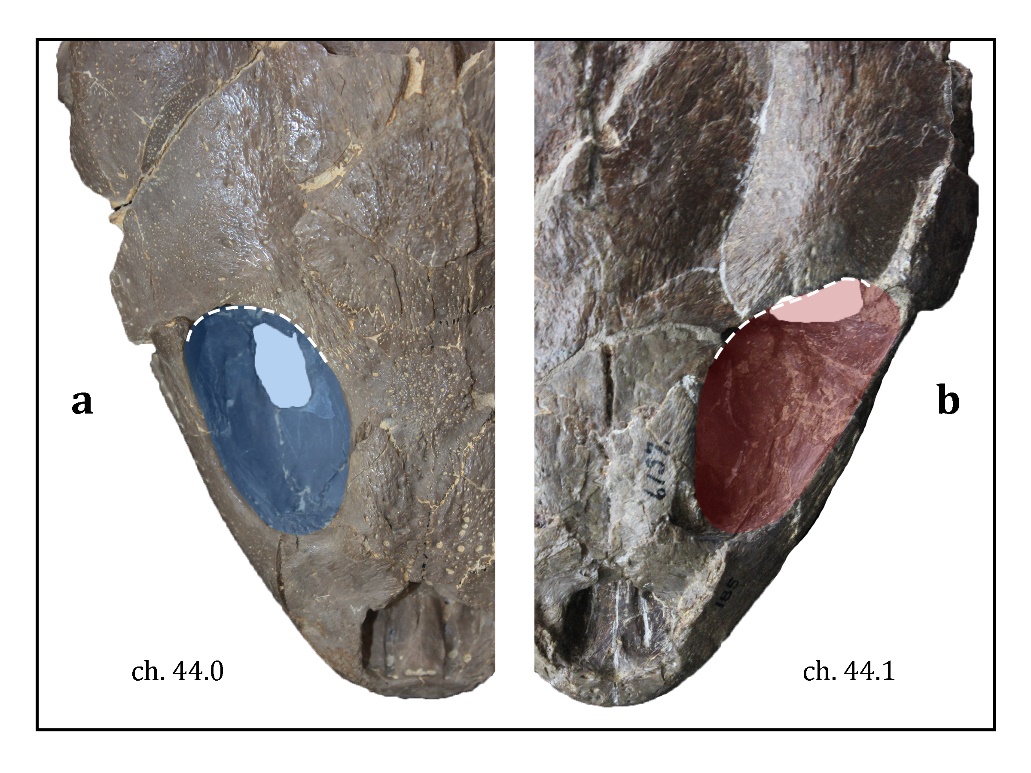


**Fig. S10.** Skulls of (a) *Toxochelys latiremis* (AMNH 5118) and (b) *Ctenochelys stenoporus* (AMNH 6137) in dorsal view showing the variation in the posterior margin of the orbit.

**Character 45**. Supratemporal: 0 = present; 1 = absent.

**Character 46.** Teeth in premaxilla, maxilla, and dentary: 0 = present; 1 = absent.

**Character 47.** Premaxilla, subdivision of the apertura narium externa by an internarial process of the premaxilla: 0 = present; 1 = absent.

**Character 48.** Premaxilla, fusion of premaxillae: 0 = absent; 1 = present.

**Character 49.** Premaxilla, foramen praepalatinum: 0 = absent; 1 = present.

**Character 50.** Premaxilla, foramen intermaxillaris: 0 = absent; 1 = present.

**Character 51.** Premaxilla, exclusion of the premaxillae from the apertura narium externa: 0 = absent; 1 = present.

**Character 52.** Premaxilla, distinct, median premaxillary hook along the labial margin of the premaxillae: 0 = absent; 1 = present.

**Character 53.** Premaxilla, cusps developed on the labial ridge in conjunction with maxilla: 0 = absent; 1 = present.

**Character 54.** Palatine, contribution to the anterior extension of the lateral braincase wall: 0 = absent; 1 = present.

**Character 55. (modified from Evers & Benson (2018))** Palatine, contribution to the upper triturating surface: 0 = absent or less than 15% of the total width of the triturating surface; 1 = moderate, between 15% and 30% of the total width of the triturating surface; 2 = extensive, more than 30% of the total width of the triturating surface.

**Character 56**. Palatine, secondary palate: 0 = absent; 1 = present, complete separation of the narial cavity from the oral cavity.

**Character 57.** Palatine, vomer-palatine contact anterior to internal naris (apertura narium interna): 0 = absent; 1 = present.

**Character 58.** Maxilla, triturating surface definition: 0 = triturating surface with labial ridge only; 1 = triturating surface with labial and lingual ridge; 2 = triturating surface with labial, lingual, and accessory ridge(s).

*Remarks*: Evers and Benson (2018) score *Toxochelys sp.* as having both a labial and lingual ridge along the maxillary triturating surface and recover this feature as an unambiguous synapomorphy of total-group Chelonioidea. *Toxochelys latiremis*: AMNH 5118 exhibits only a strong labial ridge and none of the cranial material for this species shows any indication of even a faint labial ridge. The scoring for *T. latiremis* is adjusted here to state “0”. The lingual ridge of the maxillary triturating surface is also absent in other fossil chelonioids such as *Ctenochelys stenoporus*: USNM 357166 (Matzke, 2007) and *Ctenochelys acris*: MSC 6157 (Gentry, 2016)*.*

**Character 59.** Maxilla, accessory ridge(s): 0 = accessory ridge(s) on maxilla present along the triturating surface; 1 = accessory ridge(s) only in some sectors of the triturating surface. Thic character is scored as inapplicable when no accessory ridges are present (ch 58.0 or ch 58.1).

**Character 60.** Maxilla, median contact between right and left maxilla on the palate: 0 = absent; 1 = present.

**Character 61.** Vomer, number of vomer(s): 0 = paired; 1 = single, but large; 2 = single and greatly reduced or absent.

**Character 62.** Vomer, vomer-pterygoid contact in palatal view: 0 = present; 1 = absent, medial contact of palatines present.

**Character 63.** Vomer, vomerine and palatine teeth: 0 = present; 1 = absent.

**Character 64.** Vomer, vomer-premaxilla contact in ventral view: 0 = present; 1 = absent.

**Character 65. (modified from Evers & Benson (2018))** Vomer, ventral median crest: 0 = absent, ventral surface of vomer is smooth; 1 = present, ridge extends along the ventral surface posterior to ventral process of the vomer, ridge becomes shallower posteriorly; 2 = narrow and tall ventral crest present along the full length of the vomer.

**Character 66.** Vomer, shape of the palate roof: 0 = flat; 1 = domed.

**Character 67.** Vomer, shape of anterior end contacting the maxillae and praemaxillae: 0 = flat, near horizontal contact with maxillae; 1 = the anterior end of the vomer is anteroventrally directed and laterally expanded; 2 = the anterior end of the vomer is ventrally expanded to form a horizontal footplate with a flat ventral surface.

**Character 68.** Vomer, contribution to the upper triturating surface: 0 = absent, triturating surface narrow to absent; 1 = present.

**Character 69.** Vomer, median trough on dorsal surface posterior to sulcus vomeri: 0 = absent, dorsal surface of vomer flat or transversely convex; 1 = present, dorsal surface bears a median trough that extends posteriorly from the sulcus vomeri.

*
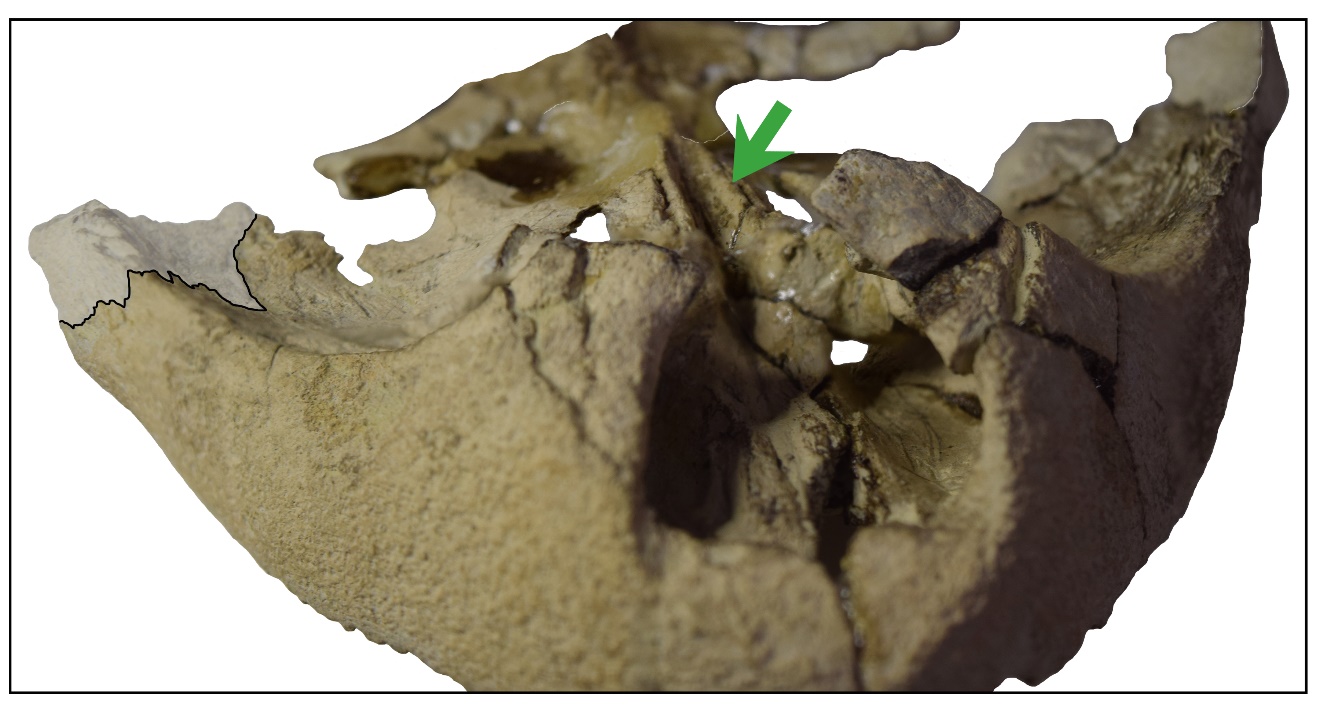
*

ch. 69.1

**Fig. S11.** Skull of *Ctenochelys acris* (MSC 6157) in anterodorsal view showing the median trough present on the dorsal surface of the pterygoid.

**Character 70***.* Foramen orbito-nasale: 0 = formed as true foramen that is surrounded by bone from all sides; 1 = foramen orbito-nasale is not completely surrounded by bone and coalescent with the passage between the fossa orbitalis and the fossa nasalis.

**Character 71.** Foramen orbito-nasale, contribution of vomer: 0 = absent; 1 = present.

*Remarks*: Evers & Benson (2018) score *Toxochelys sp.* as lacking a contribution from the vomer to the foramen orbito-nasale. Contra Evers and Benson, 2018, *Toxochelys latiremis* is scored here state “1” based on the contribution of the vomer to the foramen orbito-nasale observed by Matzke (2009). This contribution is also present in *Ctenochelys acris*: MSC 6157 (pers. obs.).

**Character 72***.* Foramen orbito-nasale, contribution of the maxilla: 0 = absent; 1 = present.

**Character 73.** Quadrate, precolumellar fossa: 0 = absent; 1 = present.

**Character 74.** Quadrate, development of the cavum tympani: 0 = shallow, but not developed anteroposteriorly; 1 = shallow, but anteroposteriorly developed; 2 = deep and anteroposteriorly developed.

**Character 75***.* Quadrate, anterior margin of the cavum tympanum: 0 =formed entirely by the quadrate; 1 = formed by the quadratojugal, which overlaps the lateral surface of the quadrate, reaching the anterior margin of the cavum tympanum.

**Character 76.** Quadrate, antrum postoticum: 0 = absent; 1 = incipient, the antrum postoticum is completely formed within quadrate (irrespective of the elements involved in forming the margin of the cavum tympanum); 2 = antrum postoticum fully developed and extending posterodorsally into the squamosal (i.e. there is a large posterodorsal fenestra in the quadrate that leads to a pocket within the squamosal). This character is scored as inapplicable for turtles without a cavum tympanum.

**Character 77.** Quadrate, incisura columellae auris: 0 = absent, stapes extends posteroventrall to quadrate body; 1 = present, but open posteroventrally; 2 = present and closed, but only enclosing the stapes; 3 = present and closed, enclosing stapes and the Eustachian tube.

**Character 78***.* Quadrate, formation of incisura columella auris: 0 = formed exclusively by quadrate; 1 = formed by quadrate and squamosal and/or quadratojugal.

**Character 79.** Quadrate, processus trochlearis oticum: 0 = absent; 1 = present, very reduced; 2 = present, large forming a well defined musculatory facet.

**Character 80.** Quadrate, contribution to the musculatory facet of the processus trochlearis oticum: 0 = extensive contribution; 1 = small contribution, facet formed principally by the protic and/or parietal. This character is scored as inapplicable when a processus trochlearis oticum is absent (ch 79.0).

**Character 81***.* Quadrate, width of processus trochlearis oticum: 0 = the otic process spans all the mediolateral space between the braincase wall and the lateral surface of the skull; 1 = the otic process is limited to the medial part of the otic chamber, and there is a deep recess laterally. This character is scored as inapplicable when a processus trochlearis oticum is absent (ch 79.0).

**Character 82.** Quadrate, quadrate/basisphenoid contact: 0 = absent; 1 = present.

**Character 83.** Quadrate, infolding ridge on the posterior surface of the quadrate ventral to the incisura columella auris: 0 = absent; 1 = present.

**Character 84.** Quadrate, direction of cranial articular process: 0 = ventrolaterally directed; 1 = with strong posterior inclination.

**Character 85***.* Posterior quadrate fossa: 0 = absent; 1 = present.

**Character 86.** Stapes, lateral articulation: 0 = stapes articulates with medial surface of the quadrate, quadrate has stapedial pit; 1 = stapes articulates with tympanic membrane, pit on medial surface of quadrate is absent.

**Character 87.** Epipterygoid: 0 = present; 1 = absent.

**Character 88.** Epipterygoid, shape: 0 = rod-like element; 1 = laminar element. This character is scored as inapplicable if an epipterygoid is absent (ch 87.1).

**Character 89.** Pterygoid, pterygoid teeth: 0 = present; 1 = absent.

**Character 90.** Pterygoid, basipterygoid process and basipterygoid articulation: 0 = basipterygoid process present with a movable basiptergoid articulation; 1 = basipterygoid process present with a sutured basipterygoid articulation; 2 = basipterygoid process absent and sutured basipterygoid articulation.

**Character 91.** Pterygoid, pterygoid/basioccipital contact: 0 = absent; 1 = present.

**Character 92.** Pterygoid, processus trochelaris pterygoideus: 0 = absent; 1 = present.

**Character 93. (modified from Evers & Benson (2018))** Pterygoid, foramen palatinum posterius: 0 = present, but open laterally; 1 = present, fully enclosed in bone; 2 = absent.

**Character 94.** Pterygoid, medial contact of pterygoid: 0 = present, pterygoids in a very long medial contact with one another, longer than the basisphenoid total length in midline; 1 = present, pterygoids in medial contact with one another, contact length equal or shorter than the basisphenoid total length in midline; 2 = absent, contact of the basisphenoid with the vomer and/or palatines present.

**Character 95.** Pterygoid, pterygoid contribution to foramen palatinum posterius: 0 = present; 1 = absent. This character is scored inapplicable when the foramen palatinum posterius is absent (ch. 66.0).

**Character 96.** Pterygoid, contact with the exoccipital: 0 = absent; 1 = present.

**Character 97.** Pterygoid, fossa podocnemidoidea or cavum pterygoidei: 0 = absent; 1 = present.

**Character 98.** Pterygoid, lateral margin: 0 = a processus pterygoideus externus is developed as a process that projects into the subtemporal fenestra; 1 = the lateral margin of the pterygoid is gently expanded laterally and/or expanded dorsoventrally; 2 = absent, i.e. the lateral margin of the pterygoid forms a straight or concave outline that forms the medial margin of the subtemporal fenestra. Scored inapplicable for pleurodires.

**Character 99.** Pterygoid, processus pterygoideus externus: 0 = forming an extensive process that contacts the maxilla anterolaterally at the posteromedial end of the triturating surface, is anteriorly sutured to the anterior palate, and has a posterior projection into the subtemporal fenestra; 1 = forming a large lateral wing that projects as a free process into the subtemporal fenestra; 2 = forming a pointed triangular process that projects laterally into the subtemporal fenestra. Scored inapplicable for taxa that lack a processus pterygoideus externus (i.e. ch 98.1 or 98.2)

**Character 100.** Pterygoid, vertical flange on anterolateral margin of the pterygoid: 0 = absent; 1 = present.

**Character 101.** Pterygoid, level of the position of the pterygoid respect to basisphenoid: 0 = both bones are at the same level on ventral surface; 1 = two different levels, creating a step between the two bones.

**Character 102.** Pterygoid, ventral median ridge: 0 = incipient to absent; 1 = present, ridge

spans nearly the full length of the pterygoids, sometimes reaching the most posterior

portion of the vomer. This character is scored as inapplicable for taxa in which the

pterygoids lack a midline contact.

**Character 103. (modified from Evers & Benson (2018))** Pterygoid, extending laterally reaching the mandibular condyle facet: 0 = absent; 1 = present, the pterygoid contacts the medial edge of the mandibular condyle in ventral view; 2 = present, the pterygoids extends not only laterally to reach the outline of the mandibular condyle facet, but also posteriorly far from the level of the condyles.

**Character 104.** Pterygoid, ventral ridge on the palatal surface lateral to skull midline. 0 = absent; 1 = present, each pterygoid has a parasagittal ridge on its ventral surface.

**Character 105.** Pterygoid, extent of ventral ridge on the palatal surface lateral to skull midline: 0 = each ridge extends along most of the ventral surface of the pterygoid, from the anteromedial margin of the pterygoid fossa to the processus pterygoideus externus; 1 = each ridge extends only along the posterior part of the pterygoid, along the level of the parabasisphenoid. This character is scored inapplicable in turtles in which ventral pterygoid ridges are absent (ch 104.0).

*Remarks*: Parasagittal ridges along the ventral surface of the pterygoid can be found in numerous Late Cretaceous stem-chelonioids (*Toxochelys latiremis* AMNH 5118, *Ctenochelys acris* MSC 6157, and *Prionochelys matutina* MSC 39030). Evers and Benson (2018) score *Toxochelys sp.* as having these ridges (ch. 104.1) but interpret their extent as being limited to the posterior section of the pterygoid along the level of the basisphenoid (ch. 105.1). Based on the explanation given by Evers and Benson (2018), their score appears to be based on a partial skull of *Toxochelys moorevillensis*: FMNH PR 219 but the pterygoids are not completely preserved with this specimen. A complete skull of *Toxochelys latiremis*: AMNH 5118 (Matzke, 2009, Text-fig. 2, p. 101) shows that these ridges extend anteriorly to the level of the processus pterygoideus externus. The score for *Toxochelys latiremis* is scored here as state 0.


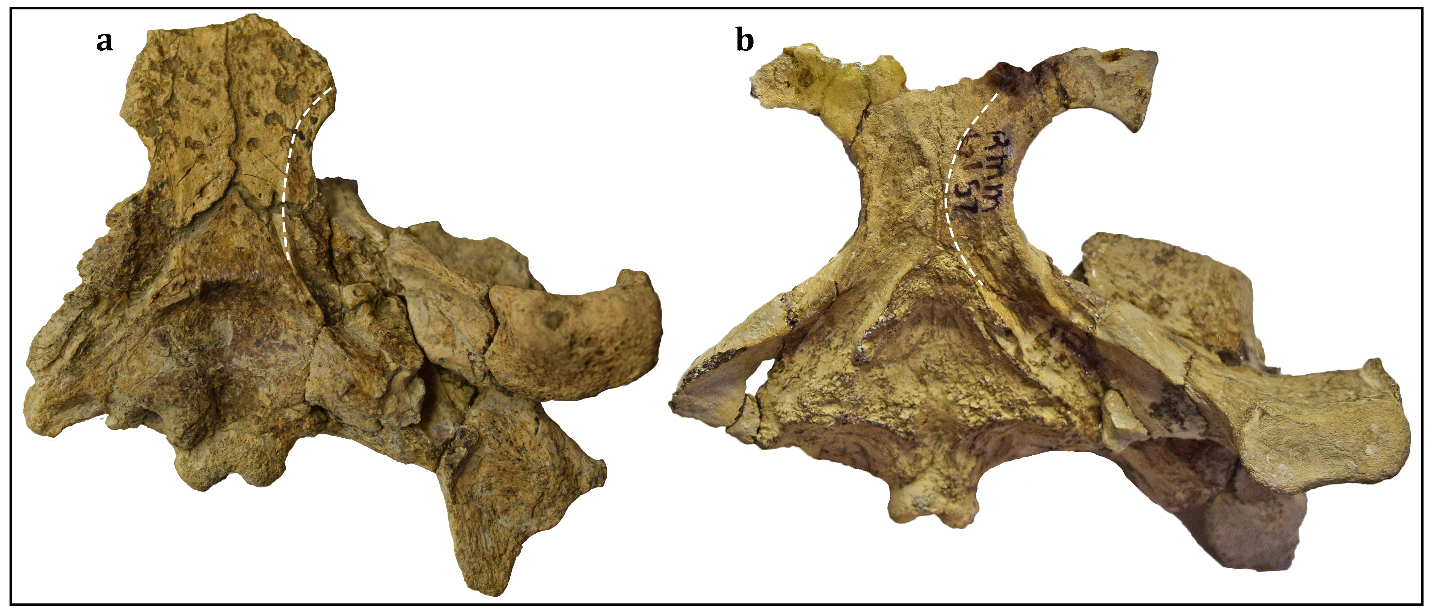


**Fig. S12.** Basicranium of (a) *Prionochelys matutina* (MSC 39030) and (b) *Ctenochelys acris* (MSC 6157) illustrating the extent of the parasagittal ridges on the ventral surface of the pterygoid.

**Character 106.** Pterygoid/Quadrate, flooring of cavum acustico-jugulare and recessus scalae typmani: 0 = absent; 1 = present, formed primarily by the posterior part of the pterygoid; 2 = present, produced by the ventral process of the quadrate or prootic or a posterolateral expansion of the parabasisphenoid.

**Character 107.** Pterygoid, posterior process: 0 = posterior process of pterygoid is absent, the cranioquadrate space or posterior foramen for the canalis cavernosus is not covered by the pterygoid; 1 = posterior process of pterygoid present but very short, process extends posteriorly to cover the posterior foramen for the canalis cavernosus (i.e. the modified cranioquadrate space), but the cavum-acustico jugulare remains largely exposed ventrally; 2 = posterior process of the pterygoid present and developed as an extensive sheet that projects posteriorly and covers large parts of the cavum acustico-jugulare.

**Character 108.** Pterygoid, development of a posteromedial wing covering partially to completely the basisphenoid and sometime the basioccipital too, seen in ventral view of the skull: 0 = absent; 1 = present.

**Character 109.** Pterygoid, pterygoid fossa: 0 = weakly developed; 1 = developed as a deep concavity between the articular process of the quadrate and the basicranium.

*Remarks*: Evers & Benson (2018) score *Toxochelys sp.* as having a weakly developed pterygoid fossa. However, AMNH 5118, AMNH 1042, and YPM 3602 (*Toxochelys latiremis*) have well-developed pterygoid fossae formed by the parasagittal and posterolateral ridges on the ventral surface of the pterygoid which closely resemble those found in *Lepidochelys olivacea* illustrated by Evers and Benson (2018) as representing the derived state for the character (Evers and Benson, 2018, Appendix 1, Fig. S1.40). The scoring for *Toxochelys latiremis* is reported here as state 1.

**Character 110.** Supraoccipital, crista supraoccipitalis: 0 = poorly developed; 1 = protruding significantly posterior to the foramen magnum.

**Character 111.** Supraoccipital, large supraoccipital exposure on dorsal skull roof: 0 = absent; 1 = present.

**Character 112.** Supraoccipital, horizontal crest in the crista supraoccipitalis: 0 = absent or poorly developed anteriorly; 1 = present, along the entire crista supraoccipitalis.

**Character 113.** Supraoccipital, fossa on the posterodorsal surface of the floor of the supratemporal fossa: 0 = absent; 1 = present, fossa is formed on the lateral surface of the supraoccipital, dorsal to the contact area between exoccipital, opisthotic, and supraoccipital.

**Character 114.** Exoccipital, medial contact of exoccipitals dorsal to foramen magnum: 0 = absent; 1 = present.

**Character 115.** Exoccipital, median contact of exoccipitals in the floor of the foramen magnum, excluding the basioccipital from the latter: 0 = absent; 1 = present.

**Character 116.** Foramen nervi hypoglossi (XII), ventral covering: 0 = exposed in ventral view; 1 = covered in ventral view by an extension of the pterygoid and the basioccipital; 2 = covered in ventral view an extension of the basioccipital; 3 = covered in ventral view by an expansion of the exoccipital and basioccipital.

**Character 117.** Exoccipital, foramina nervi hypoglossi: 0 = the foramina nervi hypoglossi exit the exoccipital on the occipital surface posterior to the margin of the fenestra postotica; 1 = at least one foramen nervi hypoglossi opens within the recessus scalae tympani anterior to margin of the fenestra postotica, and the others exit the exoccipital on the occipital surface; 2 = all foramina nervi hypoglossi open within the recessus scalae tympani anterior to the margin of the fenestra postotica.

**Character 118.** Basioccipital, morphology of the anteriormost part of the basioccipital: 0 = with two or one ventral tubercle; 1 = tubercle absent.

**Character 119.** **(modified from Evers & Benson (2018))** Basioccipital, deep C-shaped concavity between basioccipital basal tubera: 0 = absent; 1 = present.

**Character 120.** Basioccipital, basal tubera: 0 = the basal tubera are completely formed by the basioccipital; 1 = the exoccipitals and the basioccipital form the basal tubera; 2 = the pterygoids and the basioccipital form the basal tubera; 3 = the basal tubera are formed by the exoccipitals, basioccipital and pterygoids.

**Character 121.** Prootic, dorsal exposure: 0 = large; 1 = very reduced or absent.

**Character 122.** Prootic, lateral semicircular canal enclosure by bone: 0 = canal only formed by bone of the opisthotic, the prootic portion of the canal is not ossified and is instead medially confluent with the recessus labyrinthicus prooticus; 1 = prootic and opisthotic both contribute to the formation of the lateral semicircular canal.

**Character 123.** Prootic, ventral process: 0 = ventral process is short and without extensive posterior contact with the pterygoid; 1 = ventral process is large, with a broad contact with the pterygoid along a posteriorly expanded footplate, forming parts of the floor of the inner ear cavity; 2 = ventral process is extensive, and forms parts of the floor of the basicranium so that it is visible on the ventral surface of the skull. This character is scored as inapplicable for taxa that lack an anatomically modern middle ear chamber.

**Character 124.** Prootic, unnamed foramen exiting into the subtemporal fossa from the canalis cavernosus: 0 = absent; 1 = present.

**Character 125.** Prootic/pterygoid, posteroventral elongation of the trigeminal foramen: 0 = absent, the trigeminal foramen is approximately circular or oval, but the ventral margin of the foramen is positioned above the level of the canalis/sulcus cavernosus; 1 = the trigeminal foramen is oval, anterodorsally-posteroventrally elongate and anteroventrally-posterodorsaly narrow, and the ventral margin is level with the canalis/sulcus cavernosus. This character is scored inapplicable for turtles without a trigeminal foramen (i.e. without a neomorphic secondary lateral wall of the braincase formed by the parietal and pterygoid).

*Remarks*: Evers & Benson (2018) record the presence of an elongate trigeminal foramen in certain cheloniids, xinjianchelyids, and chelydroids. Despite some degree of taphonomic distortion, several Late Cretaceous chelonioids appear to have also possessed posteroventrally elongate trigeminal foramina whose ventral margin lies at the level of the floor of the canalis cavernosus (*Toxochelys latiremis*: AMNH 1042 (Matzke, 2009, Text-fig. 9, p. 111) and *Ctenochelys acris*: MSC 6157 (pers. obs.). *Toxochelys latiremis* is scored here as state 1.

**Character 126.** Prootic, recess on posterior surface of the element anterodorsolaterally to the fenestra ovalis: 0 = absent; 1 = present.

**Character 127.** Prootic, position of the geniculate ganglion and the split of the facial nerve into the hyomandibular and palatine branches: 0 = the geniculate ganglion is positioned with the canalis cavernosus; 1 = the geniculate ganglion is positioned within the facial nerve canal; 2 = the geniculate ganglion is positioned in the canalis carotici interni.

**Character 128.** Prootic/opisthotic, enclosure of fenestra ovalis: 0 = the fenestra ovalis is ventrally enclosed by the prootic and opisthotic; 1 = the prootic and opisthotic do not have a contact ventrally to the fenestra ovalis.

**Character 129.** Opisthotic, wide transverse occipital plane with depression for the nuchal musculature: 0 = absent; 1 = present.

**Character 130.** Opisthotic, processus interfenestralis: 0 = developed as a robust ridge that does not form a ventrally projecting process and does not reach closely to the floor of the basicranium; 1 = incipient, developed as a ventrally low and mediolaterally broad, robust structure that separates the cavum labyrinthicum anteriorly from an incipient recessus scalae tympani posteriorly; 2 = present, developed as a ventrally directed process that separates the cavum labyrinthicum anteriorly from the recessus scalae tympani posteriorly.

**Character 131.** Opisthotic, development of the processus interfenestralis: 0 = developed as a ventrally directed process that separates the cavum labyrinthicum anteriorly from the recessus scalae tympani posteriorly, the process is not expanded at its ventral end, and almost or just about reaches the floor of the basicranium, but a small gap (hiatus postlagenum) usually remains; 1 = developed as a ventrally directed process that separates the cavum labyrinthicum anteriorly from the recessus scalae tympani posteriorly, but the process has a horizontally expanded footplate at its ventral end that is sutured to elements of the basicranium; 2 = developed as a ventrally directed process that separates the cavum labyrinthicum anteriorly from the recessus scalae tympani posteriorly, and the ventral surface of the process is integrated into the basicranium to form parts of the ventral surface of the cranium. This character is scored as inapplicable if an anatomically modern processus interfenestralis is absent (i.e. ch 131.0 or 131.1).

**Character 132.** Opisthotic, posterior surface of paroccipital process: 0 = the occipital side of the paroccipital process is developed as a posteriorly exposed, broad, planar or gently concave surface; 1 = the occipital side of the paroccipital process is dorsoventrally flattened and forms a posterior ridge that traverses the paroccipital process mediolaterally; 2 = the paroccipital process has a dorsoventrally convex surface.

**Character 133.** Fenestra perilymphatica: 0 = large; 1 = reduced in size to that of a small foramen.

**Character 134.** Parabasisphenoid, paired pits on ventral surface of basisphenoid: 0 = absent; 1 = present.

**Character 135.** Parabasiphenoid, ventral surface: 0= flat to slightly convex, with posterior margin straight or slightly concave; 1= V-shaped crest, with posterior margin forming the basipterygoid process projected posterolaterally.

**Character 136.** Parabasiphenoid, rough surface between basisphenoid and basioccipital: 0 = absent; 1 = present.

**Character 137.** Parabasisphenoid, posterolateral processes lapping onto the ventral surface of the basioccipital: 0 = absent; 1 = present.

**Character 138.** Parabasisphenoid, rostrum basisphenoidale: 0 = flat; 1 = flat base, but with trabeculae contact one another medially forming a short rod at the anterior end of the parabasisphenoid; 2 = singular median, rod-like, thick and rounded process.

*Remarks*: The rostrum basisphenoidale of *Toxochelys latiremis* is posteriorly comprised of two cylindrical trabeculae which at their posterior end are widely separated but contact medially slightly anterior to the anterior exits of the palatine arteries (AMNH 1042: Maztke, 2009, Text-fig. 10, p. 113). *Toxochelys latiremis* is scored as having the intermediate condition (state 1) for this feature.

**Character 139.** Parabasisphenoid, dorsum sellae: 0 = deep, i.e. the dorsal surface of parabasisphenoid between the clinoid processes is a transversely concave floor forming a trough between the posterior part of the dorsal surface of the parabasisphenoid, which is usually cup-like, and the anterior portion of the parabasisphenoid forming the rostrum basisphenoidale and sella turcica; 1 = low, i.e. the dorsum sellae is formed as a transverse ridge between the clinoid processes that projects anteriorly at a low angle from posterodorsal surface of the parabasisphenoid; 2 = high, i.e. a transverse ridge or wall of bone between the clinoid processes is present that projects dorsally at a high angle from the posteriorly positioned cup, separating the cup very clearly from of the anteriorly positioned rostrum basisphenoidale and sella turcica.

*Remarks*: The dorsum sellae of *Toxochelys latiremis* is a transversely concave ridge between the clinoid processes which projects slightly anterodorsally from the anterior margin of the dorsal ‘cup-like’ portion of the parabasisphenoid to overlap the posterior margin of the sella turcica (AMNH 1042; Matzke, 2009, Text-figs. 8-10). We assign the intermediate condition (state 1) to this species. The derived condition (state 2) as defined by Evers & Benson (2018), can be seen in *Ctenochelys acris*: MSC 6157 (Gentry, 2016, Fig. 7, p. 9) and *Ctenochelys stenoporus*: USNM 357166 (Matzke, 2007, Text-fig. 9, p. 680).

**Character 140.** Parabasisphenoid, anterior surface of dorsum sellae: 0 = anterior surface of the dorsum sellae is flat and smooth; 1 = vertical median ridge on anterior surface of dorsum sellae between the clinoid processes is present, ridge may have a small anterodorsal projection.


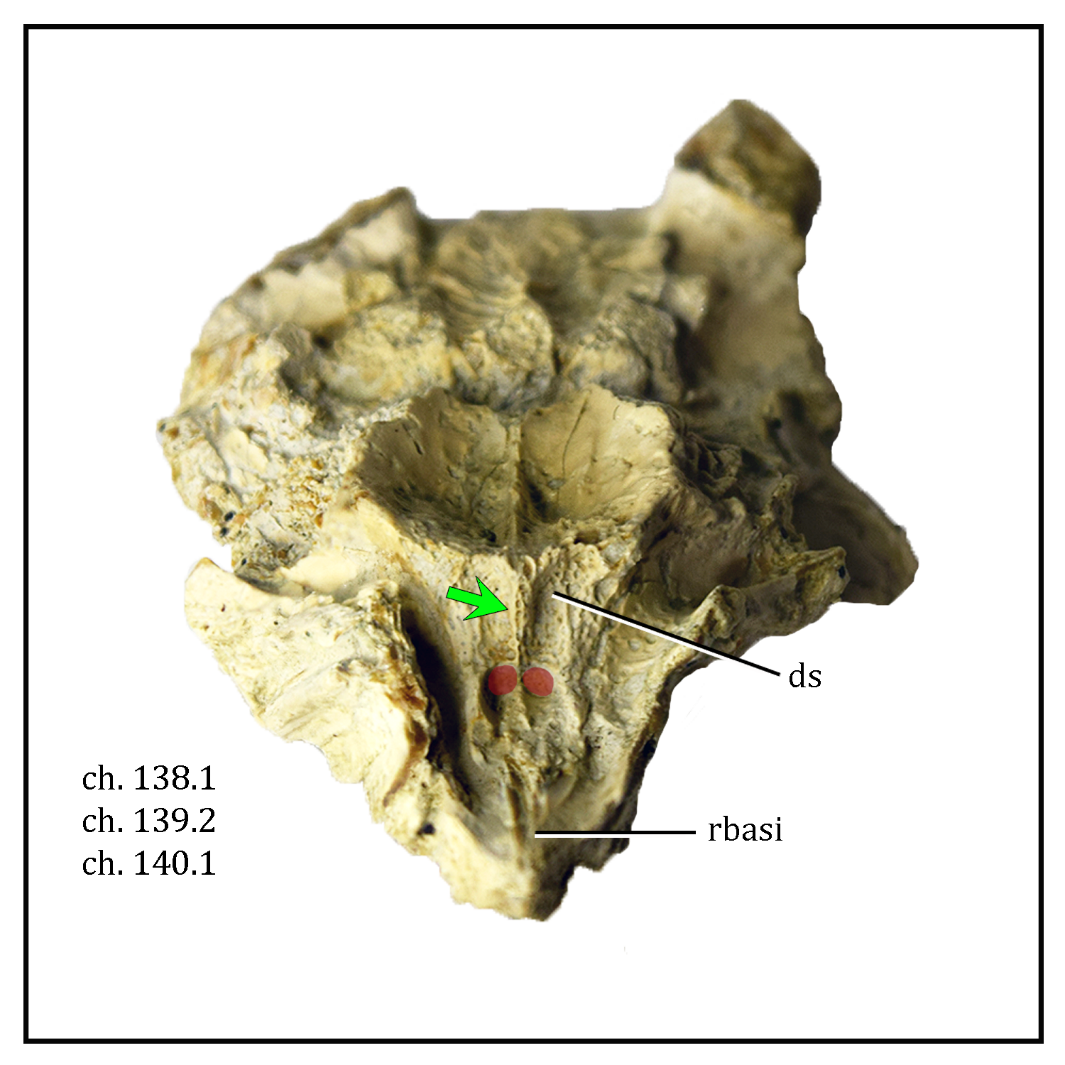


**Fig. S13.** Basicranium of *Ctenochelys acris* (MSC 3050) illustrating the derived chelonioid features present in certain Late Cretaceous non-protostegid stem chelonioids including the trabeculae of the rostrum basisphenoidale (ch. 138.1), the high dorsum sellae (ch. 139.2), and the vertical median ridge (green arrow) on the anterior surface of the dorsum sellae (ch. 140.1). Abbreviations: ds, dorsum sellae; rbasi, rostrum basisphenoidale.

**Character 141.** Parabasisphenoid, retractor bulbi pits on anterolateral surface: 0 = absent; 1 = present.

**Character 142.** Parabasisphenoid, foramina anterius canalis carotici cerebralis: 0 = widely separated; 1 = close together; 2 = right and left cerebral arteries converge within the parabasisphenoid and run within a short joint canal and exit anteriorly into the cavum cranii via a single foramen within the sella turcica.

**Character 143.** Parabasisphenoid, prootic foramen in primary lateral wall of the braincase: 0 = present, the clinoid process of the parabasisphenoid and parts of the prootic, an ossified pila antotica, the laterosphenoid, and/or parts of the parietal form a foramen medially to the cavum epiptericum; 1 = the prootic foramen is reduced, and the clinoid processes are free structures.

**Character 144.** Parabasisphenoid, crista tuberculi basalis developed on posterior part of dorsal surface: 0 = absent, parabasisphenoid dorsal surface is relatively flat or gently concavely excavated; 1 = present as median, dorsally high projecting tubercle or ridge.

**Character 145.** Carotid artery/Pterygoid, interpterygoid vacuity: 0 = large opening, triangular in shape; 1 = reduced to an interpterygoid slit; 2 = entirely closed, no foramina posterius canalis carotici palatinum present. Scored inapplicable for taxa with a bony canal for the palatine artery (ch 146.1)

**Character 146.** Embedding of the palatine division of carotid artery: 0 = the palatine artery is not encased in a bony canal (artery enters the skull through the interpterygoid cavuity, interpterygoid slit, or possibly other skull openings); 1 = the palatine artery is encased in a bony canal (irrespective of the exposure or embedding of the internal carotid artery split).

**Character 147.** Embedding of internal carotid artery and its bifurcation: 0 = Internal carotid arterial system is not embedded by bone, a foramen posterius canalis carotici is absent, and the split into palatine and cerebral artery occurs extracranially; 1 = internal carotid arterial system is partially embedded, a foramen posterius canalis carotici interni is present, and the split into palatine and cerebral artery happens at the fenestra caroticus and is thus ventrally exposed; 2 = internal carotid arterial system is ventrally fully embedded by bone, a foramen posterius canalis carotici interni is present and the split into palatine and cerebral artery is ventrally covered by bone.

**Character 148.** Position of the foramen posterius canalis carotici interni (fpcci): 0 = the fpcci is located at the ventral surface of the skull in a position far anterior to the margin of the fenestra postotica; 1 = the fpcci is located at the posterior end of the skull, either on the ventral surface of the skull close to the margin of the fenestra postotica, or on the posterior surface of the skull at the ventral margin of the fenestra postotica. This character is scored inapplicable for taxa that lack a foramen posterius canalis carotici interni.

**Character 149.** Entry of the internal carotid artery into skull relative to cavum acustico-jugulare: 0 = internal carotid enters the skull ventrally to the level of the cavum acustico-jugulare; 1 = internal carotid artery enters the skull within the cavum acustico-jugulare, artery lies in dorsally open trough on dorsal surface of pterygoid and only becomes dorsally covered within the cavum acustico-jugulare.

**Character 150.** Dorsal exposure of the palatine artery and/or anterior parts of the internal carotid artery: 0 = absent, the split of the internal carotid artery is dorsally covered by bone, and the palatine artery exits the basicranium into the cavum cranii via the foramen anterius canalis carotici palatinum; 1 = the internal carotid artery exits the braincase anterodorsally into the sulcus cavernosus where it continues on the floor of the sulcus cavernosus (= sulcus caroticus of Gaffney 1979), and the split into cerebral and palatine arteries occurs within the sulcus cavernosus. The cerebral artery enters the parabasisphenoid medially, whereas the palatine artery continues in the sulcus cavernosus anteriorly without ever being embedded in a canal. This character is scored as inapplicable when the carotid split is ventrally exposed (i.e. ch 148.0 or 148.1).

*
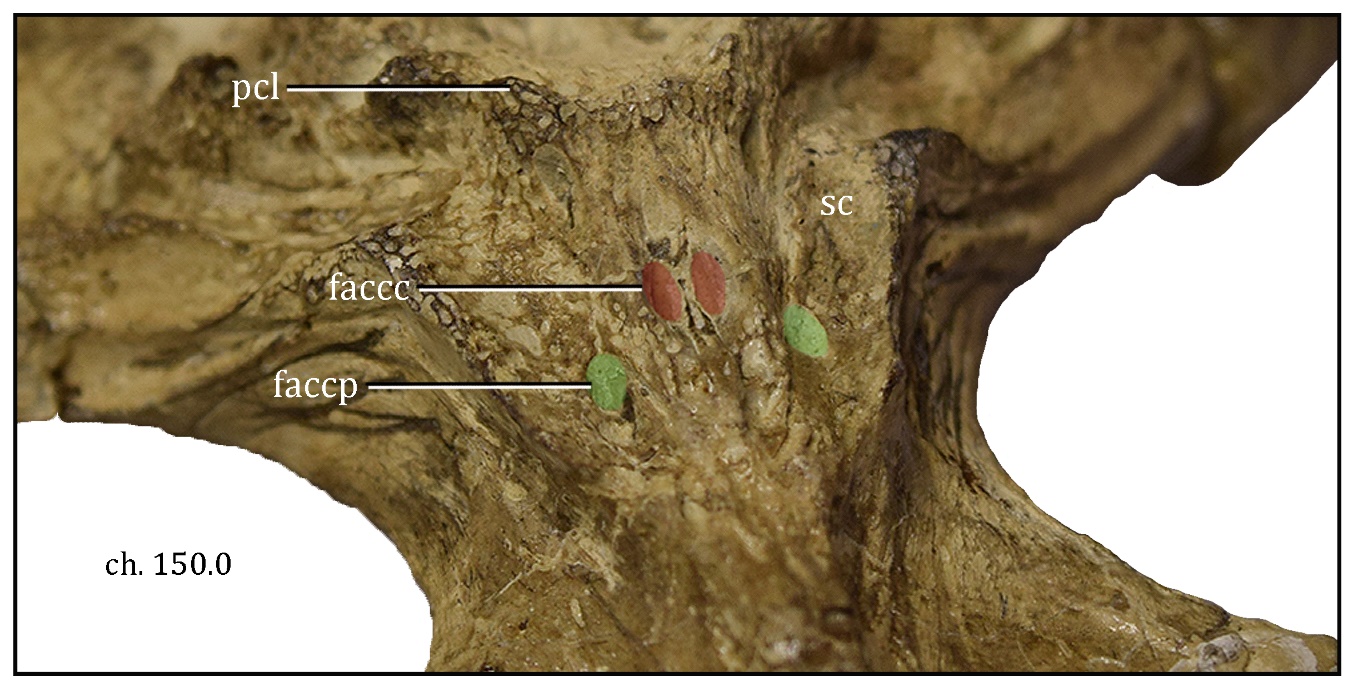
*

**Fig. S14.** Basicranium of *Ctenochelys acris* (MSC 6157) showing the exits of the palatine (green) and cerebral (red) branches of the internal carotid artery. Abbreviations: faccc, foramen anterius canalis carotici cerebralis; faccp, foramen anterius canalis carotici palatinum; pcl, processus clinoideus; sc, sulcus cavernosus.

**Character 151.** Formation of the foramen posterius canalis carotici interni, pterygoid involvement: 0 = present; 1 = absent.

**Character 152.** Formation of the foramen posterius canalis carotici interni, parabasisphenoid involvement: 0 = present; 1 = absent.

**Character 153.** Formation of the foramen posterius canalis carotici interni, quadrate involvement: 0 = present; 1 = absent.

**Character 154.** Formation of the foramen posterius canalis carotici interni, prootic involvement: 0 = present; 1 = absent.

**Character 155.** Hyomandibular branch of the facial nerve: 0 = contained within the canalis cavernosus; 1 = contained in a sulcus or separate canal paralleling the canalis cavernosus.

**Character 156.** Stapedial artery, foramen stapedio-temporale: 0 = present; 1 = absent.

**Character 157.** Stapedial artery, size of foramen stapedio-temporale: 0 = relatively large (the size of a large blood foramina, ≥5 mm diameter); 1 = significantly reduced in size (the size of a nerve foramina, ≤3 mm diameter. This character is scored as inapplicable for taxa without a foramen stepediotemporale (i.e. ch 156.1).

**Character 158.** Stapedial artery, foramen stapedio-temporale location in the otic chamber: 0 = on dorsal part and pointing dorsally; 1 = on the anterior wall of the otic region, pointing anteriorly. This character is scored as inapplicable for taxa without a foramen stepedio-temporale (i.e. ch 156.1).

**Character 159.** Stapedial artery, formation of the foramen stapediotemporale, contribution of the quadrate: 0 = absent; 1 = present. Scored inapplicable for taxa without a foramen stapedio-temporale. This character is scored as inapplicable for taxa without a foramen stepedio-temporale (i.e. ch 156.1).

**Character 160.** Stapedial artery, formation of the foramen stapediotemporale, contribution of the prootic: 0 = absent; 1 = present. Scored inapplicable for taxa without a foramen stapedio-temporale. This character is scored as inapplicable for taxa without a foramen stepedio-temporale (i.e. ch 156.1).

**Character 161.** Stapedial artery, formation of the foramen stapediotemporale,

contribution of the opisthotic: 0 = absent; 1 = present. Scored inapplicable for taxa without a foramen stapedio-temporale. This character is scored as inapplicable for taxa without a foramen stepedio-temporale (i.e. ch 156.1).

**Character 162.** Stapedial artery, formation of the foramen stapediotemporale, contribution of the supraoccipital: 0 = absent; 1 = present. This character is scored as inapplicable for taxa without a foramen stepedio-temporale (i.e. ch 156.1).

**Character 163.** Foramen jugulare posterius, relationship with the fenestra postotica: 0 = separate from fenestra postotica; 1 = coalescent with fenestra postotica.

**Character 164.** Foramen jugulare posterius, formation of lateral bar separating foramen from fenestra postotica: 0 = formed by pterygoid; 1 = formed by opisthotic and/or exoccipital. The character is scored inapplicable for taxa in which the foramen jugulare posterius is coalescent with the fenestra postotica (ch. 163.1).

**Character 165.** Recessus scalae tympani: 0 = almost nonexistent, not surrounded by bone; 1 = well developed.

**Character 166.** Cranial scutes, scute D meeting in midline: 0 = absent; 1 = present.

**Character 167.** Cranial scutes, scute X much smaller than scute D: 0 = absent; 1 = present.

**Character 168.** Cranial scutes, scute X partially separates scutes G: 0 = absent; 1 = present.

**Character 169.** Cranial scutes, scutes A, B, and C forming a continuous posterolateral shelf: 0 = absent; 1 = present.

**Character 170.** Cranial scutes, scute F: 0 = formed by several scutes; 1 = formed by a single scute.

**Character 171.** Cranial scutes, scute J: 0 = formed by several scutes; 1 = formed by a single scute.

**Character 172.** Dentary, medial contact of dentaries: 0 = fused; 1 = open suture.

**Character 173.** Dentary, width triturating surface vs. jaw length: 0 = narrow triturating surface, symphysis less than 1/3 of jaw length; 1 = broad triturating surface, symphysis ≥1/3 jaw length.

**Character 174.** Dentary, symphyseal ridge: 0 = absent; 1 = present.

*Remarks*: *Toxochelys latiremis* possesses a shallow symphyseal ridge visible in dorsal view along the suture between the left and right dentary (Matzke, 2009, Text-figs. 11-13). Contra Evers & Benson (2018), *Toxochelys latiremis* is scored here as having the derived state of the character (state 1).

**Character 175.** Dentary, lingual (tomial) ridge: 0 = prominent; 1 = weak or absent.

**Character 176.** Dentary, size of foramen dentofaciale majus: 0 = small, size of a small vessel; 1 = enlarged, foramen is several mm in diameter.

*Remarks*: Evers & Benson (2018) score *Toxochelys sp.* as polymorphic for this character based on the presence of a small foramen dentofaciale majus in AMNH 5118 and a large foramen in a different specimen, AMNH 14221. However, AMNH 14221 consists of a single humerus from the Pierre Shale of South Dakota, originally identified as *Toxochelys browni*, a junior synonym of *Toxochelys latiremis* (*sensu* Nicholls, 1988). AMNH 14221 is not listed by Evers and Benson (2018) as a specimen used for scoring *Toxochelys latiremis* (Evers & Benson (2018), Table 1, p. 9) and since no lower jaw is preserved with this specimen, the scoring for *T. latiremis* is herein adjusted to state 0 to reflect the condition observed in AMNH 5118 (Matzke, 2009, Text-fig. 12, p. 119).

**Character 177.** Dentary-Surangular arrangement: 0 = lack of a posterior expansion of dentary and anterior projection of surangular; 1 = posterior expansion of dentary present almost reaching the articular surface, covering the dorsal half of the surangular in lateral view, surangular with anterior projection.

**Character 178.** Surangular, with anteromedial process forming a vertical lamina that projects anteriorly into the fossa meckelii: 0 = absent; 1 = present.

**Character 179.** Coronoid, anteromedial process: 0 = absent; 1 = present.

**Character 180.** Coronoid, contribution to triturating surface: 0 = absent; 1 = present.

**Character 181.** Coronoid, notch on posterior margin of coronoid: 0 = absent; 1 = present.

**Character 182.** Coronoid, foramen at anterior end, leading from fossa meckelii into space between mandibular rami: 0 = absent; 1 = present.

*Remarks*: Evers & Benson (2018) score *Toxochelys sp.* as possessing an unnamed foramen on the anteromedial portion of the coronoid (state 1). However, in AMNH 5118 (*Toxochelys latiremis*) no such foramen is present, scored here as state 0. Evers & Benson (2018) also note the presence of this foramen in *Lepidochelys olivacea*: SMNS 11070 but then figure SMNS 11070 as lacking this foramen (Appendix 1, Fig. S1.72). We change the scoring here to reflect the condition figured by Evers and Benson (2018) and not the character state presented in the supplemental text.

**Character 183.** Coronoid process, principally formed by: 0 = coronoid; 1 = dentary; 2 = surangular.

**Character 184.** Coronoid process: 0 = relatively low, dorsally well rounded; 1 = relatively high, process is dorsally or posterodorsally pointed.

**Character 185.** Splenial: 0 = present; 1 = absent.

**POSTCRANIUM**

**Character 186.** Carapace, carapacial scutes: 0 = present; 1 = absent.

**Character 187.** Carapace, carapacial scutes: 0 = present, fully covering the carapace; 1 = reduced not fully covering the carapace.

**Character 188.** Carapace, continuous keel on costals: 0 = absent; 1 = present.

**Character 189.** Carapace, continuous keel on neurals: 0 = absent; 1 = present.

**Character 190.** Shell, sculpturing of dorsal surface (carapace) and ventral surface (plastron): 0 = absent, smooth to slightly rugose; 1 = present, development of striations, vermiculations, striations, or pitting.

**Character 191.** Shell, pattern of sculpturing of the dorsal surface (carapace) and ventral surface (plastron): 0 = parallel to radial striations; 1 = vermiculation; 2 = highly dense pattern of pitting combined with striations; 3 = dichotomic striations; 4 = spread pitting without marked striation pattern; 5 = granules (positive relief).

**Character 192.** Carapacial Sutures: 0 = carapacial elements finely sutured or the contact is smooth; 1 = carapacial sutures strongly serrated in adult stage.

**Character 193.** Nuchal, articulation of nuchal with neural spine of eighth cervical vertebra: 0 = articulation via a blunt facet is present; 1 = articulation along a blunt facet absent.

**Character 194.** Raised pedestal on the visceral surface of the nuchal for the articulation with the neural spine of the eighth cervical vertebra: 0 = absent; 1 = present.

**Character 195.** Nuchal, elongate costiform process: 0 = absent; 1 = present.

**Character 196.** Nuchal, length of costiform process: 0 = crosses peripheral 1; 1 = reaches peripherals 2 or 3. This character is scored inapplicable for taxa without a costiform process on the nuchal (ch. 195.0).

**Character 197.** Nuchal, length versus width: 0 = wider than long; 1 = longer than wide or as long as wide.

**Character 198.** Nuchal, posteriomedial fontanelles: 0 = absent; 1 = present.

**Character 199.** Neurals, neural formula 6>4<6<6<6<6: 0 = absent; 1 = present.

**Character 200.** Neurals, shape of neurals: 0 = very irregular in shape, wider than long or squared; 1 = regular, often perfectly hexagonal or pentagonal, longer than wide.

**Character 201.** Neurals: 0 = present; 1 = absent.

*Remarks*: Evers & Benson (2018) score several species of crown cheloniid as lacking neurals. However, crown cheloniids all possess neurals, scored here as state 0.

**Character 202.** Neurals, number of neurals: 0 = ten or more; 1 = nine or less.

*Remarks*: Evers & Benson (2018) score every crown cheloniid except *Natator depressus* and *Caretta caretta* as lacking neurals then subsequently score these same taxa as ‘inapplicable’ for character 202. Contra Evers and Benson (2018), *Chelonia mydas* is scored here as having nine neurals (state 1), *Lepidochelys kempii* as having ten or more neurals (state 0; Wyneken, 2001), *Lepidochelys olivacea* as having nine neurals (state 1; John et al., 2011), and *Eretmochelys imbricata* as having nine neurals (state 1; Wyneken, 2001).

**Character 203 (new character).** Epineurals: 0 = absent; 1 = present.

*Remarks*: Although many previous turtle matrices have incorporated characters pertaining to the presence or absence of keeled neurals (Evers & Benson (2018): character 189, Cadena & Parham (2015): character 116, Joyce (2007): character 61, Sterli & de la Fuente (2013): character 122, (Carapace B) and Hirayama (1998), Kear & Lee (2006), and Bardet et al. (2013) (character 84)), the variation regarding the elements which comprise this keel has yet to be considered. Several fossil chelonioids (*Ctenochelys stenoporus*: USNM 357166, *Ctenochelys acris*: MSC 35085, *Prionochelys matutina*: MSC 3036, *Peritresius ornatus*: NJSM 11051) possess epineurals dorsal to the neural series that increase the overall height of the median neural keel at various intervals (Gentry, 2018). This character attempts to capture the phylogenetic information contained in the shared presence of these elements.

**Character 204 (Evers & Benson (2018) character: 203).** Peripheral Gutter: 0 =

peripheral gutter absent of only anteriorly developed; 1 = peripheral gutter extensively

developed along anterior and bridge peripherals.

**Character 205 (Evers & Benson (2018) character: 204).** Peripherals: 0 = present;

1 = absent.

**Character 206 (Evers & Benson (2018) character: 205).** Peripherals, number of

peripherals: 0 = more than 11 pairs of peripherals present; 1 = 11 pairs of peripherals

present; 2 = 10 pairs of peripherals present. This character is scored as inapplicable

when peripherals are absent (ch. 204.1).

**Character 207 (Evers & Benson (2018) character: 206).** Peripherals, anterior

peripherals incised by musk ducts: 0 = absent; 1 = present.

**Character 208 (Evers & Benson (2018) character: 207).** Costals, medial contact of

the first pair of costals: 0 = absent; 1 = present.

**Character 209 (Evers & Benson (2018) character: 208).** Costals, medial contact of

posterior costals: 0 = absent; 1 = present.

**Character 210 (Evers & Benson (2018) character: 209).** Costals, number of costals

involved in medial contact: 0 = medial contact of up to three posterior costals; 1 =

medial contact of all costals. This character is scored as inapplicable for turtles without a

medial contact of posterior costals.

**Character 211 (Evers & Benson (2018) character: 210).** Costals, distal rib end and

lateral ossification of the costal: 0 = costals fully ossified laterally with strong sutural

contact with peripherals, lack of dorsal exposure of distal end of costal ribs; 1 = costals

fully ossified laterally with strong sutural contact with peripherals, distal end of costal

ribs exposed on dorsal surface and surrounded by the peripheral; 2 = costals lack lateral

ossification, allowing the dorsal exposure of the distal end of ribs and the development

of fontanelles only at the most anterior and posterior costals; 3 = costals with extreme

lost of lateral ossification, allowing the dorsal exposure of the distal end of ribs, in

almost all series of costals.

*Remarks*: Contra Evers and Benson (2018), the following taxa are scored here as exhibiting an extreme loss of lateral costal ossification with the development of extensive fontanelles in all series of costals: *Allopleuron hofmanni* (Mulder, 2003), *Protostega gigas* (= *Protostega dixiei*, Zangerl, 1953a), and *Toxochelys latiremis* (see Zangerl, 1953b, YPM 3602, Fig. 74, p. 187).

**Character 212 (modified from Evers & Benson (2018)).** Rib-free peripherals between the first and last rib: 0 = absent; 1 = present.

*Previous definition* (Evers & Benson (2018): character 211): Rib-free peripherals: 0 =

absent; 1 = present.

*Remarks*: We agree with Evers and Benson (2018) in the coding of separate characters for the presence vs. absence of rib-free peripherals between the first and last rib and the variation regarding their relative position however, we include a modified definition in order to clarify the nature of the phylogenetic information being captured. This character was created by Parham & Fastovsky (1997: character 21) to capture the phylogenetic signal in the shared presence of rib free peripherals within the rib series observed in certain crown-cheloniids (e.g. *Chelonia*, *Eretmochelys*, and *Caretta*). Originally proposed as a single character, Parham & Fastovsky (1997) included a primitive character state “0” to reflect the condition found in most turtles where rib-free peripherals are present only anterior and posterior to the rib series. The modified character definition now clearly pertains only to those taxa which possess rib-free peripherals within the rib series and so the state “only present anterior and posterior to the ribs” is no longer needed in the subsequent character (ch. 213) as all taxa with this condition can simply be scored as “absent” (ch. 212.0). This arrangement more adequately describes the potential homology the original character intended to capture without sacrificing the additional phylogenetic information gained from the division of the original character.

**Character 213 (modified from Evers & Benson (2018)).** Position of rib-free peripherals: 0 = present between sixth and seventh ribs; 1 = present between the seventh and eighth ribs.

*Previous definition* (Evers & Benson (2018): character 212): Position of rib-free peripherals: 0 = only present anterior and posterior to ribs; 1 = present between sixth and seventh ribs. This character is scored as inapplicable for taxa that lack rib free peripherals.

*Remarks*: The state “present between the seventh and eighth ribs” was removed by Evers & Benson (2018) but is added back to the character definition here due to this condition being observed in *Ctenochelys stenoporus*: USNM 357166 (Matzke, 2007) and *Chelonia mydas* (Parham & Fastovsky, 1997).

**Character 214 (Evers & Benson (2018): character 213).** Costals, alternative short

and long ends in the lateral part of costals: 0 = absent; 1 = present.

**Character 215 (Evers & Benson (2018): character 214).** Costals, costal 9: 0 =

present; 1 = absent.

**Character 216 (Evers & Benson (2018): character 215).** Costals, shape of Costal 3:

0 = tapering towards the lateral side of the shell or with parallel anterior and posterior

borders; 1 = broadens towards the lateral side of the shell.

**Character 217 (Evers & Benson (2018): character 216).** Suprapygals: 0 = present;

1 = absent.

**Character 218 (Evers & Benson (2018): character 217).** Suprapygals, number of

suprapygals: 0 = one; 1 = two; 2 = more than two.

*Remarks*: Contra Evers & Benson (2018), *Allopleuron hofmanni* is scored here as having more than 2 suprapygals (see Mulder, 2003, IRScNB EFR 8, Pl. 24, p. 64).

**Character 219 (Evers & Benson (2018): character 218).** Suprapygals, size between

suprapygal 1 and 2: 0 = suprapygal 1 smaller than suprapygal 2; 1 = suprapygal 1 larger. Turtles with only one suprapygal or suprapygals absent are coded as inapplicable.

**Character 220 (Evers & Benson (2018): character 219).** Cervical scutes: 0 =

present; 1 = cervical scutes absent.

**Character 221 (Evers & Benson (2018): character 220).** Number of cervical scutes:

0 = more than one cervical scute present; 1 = one cervical scute present. This character

is scored as inapplicable when cervical scutes are absent (ch. 219.1)

**Character 222 (Evers & Benson (2018): character 221).** Pygal, posterior notch: 0 =

present; 1 = absent.

*Remarks*: Evers & Benson (2018) score *Toxochelys sp.* as lacking a notch on the posterior margin of the pygal. In her description of *Toxochelys latiremis* (ROM 25683), Nicholls (1988) describes the pygal as having a smooth, convex posterior margin. However, the photograph provided by Nicholls (1988, Fig. 1A, p. 182) shows that a posterior notch is present. *Toxochelys latiremis* is scored here as state 0.

**Character 223 (Evers & Benson (2018): character 222).** Supramarginals: 0 =

present; 1 = absent.

**Character 224 (Evers & Benson (2018): character 223).** Supramarginals,

separating marginal and pleurals: 0 = complete row present, fully separating marginals

from pleurals; 1 = partial row present, incompletely separating marginals from pleurals.

This character is scored as inapplicable for turtles in which supramarginals are absent.

**Character 225 (Evers & Benson (2018): character 224).** Vertebrals, shape of the

verterbrals: 0 = vertebrals 2 to 4 signicantly broader than pleurals; 1 = vertebrals 2 to 4

as narrow as, or narrower than, pleurals.

**Character 226 (Evers & Benson (2018): character 225).** Vertebrals, position of

vertebral 3-4 sulcus in taxa with five vertebrals: 0 = sulcus positioned on neural 6; 1 =

sulcus positioned on neural 5.

**Character 227 (Evers & Benson (2018): character 226).** Vertebrals, vertebral 3-4

sulcus with a wide posteriorly oriented medial embayment: 0 = absent; 1 = present.

**Character 228 (Evers & Benson (2018): character 227).** Vertebrals, verebral 1: 0 =

vertebral 1 does not enter anterior margin of carapace; 1 = enters anterior margin.

**Character 229 (Evers & Benson (2018): character 228).** Marginals, marginal

scutes overlap onto costals: 0 = absent, marginals restricted to peripherals; 1 = present.

**Character 230 (Evers & Benson (2018): character 229).** Pleurals, at least one pair

of additional pleural scutes located laterally of vertebral scute 1, with anterior contact

with cervical scute: 0 = absent; 1 = present.

**Character 231 (Evers & Benson (2018): character 230).** Plastron, connection

between carapace and plastron: 0 = osseous; 1 = ligamentous.

**Character 232 (Evers & Benson (2018): character 231).** Plastron, central plastral

fontanelle: 0 = absent; 1 = present.

**Character 233 (Evers & Benson (2018): character 232).** Plastron, posterior

plastral fontanelle, posterior plastral fontanelle between the xiphiplastra and/or the

hypoplastra: 0 = absent in adult stage; 1 = retained in adult stage.

**Character 234 (Evers & Benson (2018): character 233).** Plastron, plastral kinesis:

0 = absent, scutes sulci and bony sutures do not overlap; 1 = present, scutes sulci

coincide with epiplastral-hyoplastral contact.

**Character 235 (Evers & Benson (2018): character 234).** Plastron, plastral kinesis:

0 = between hyoplastron and hypoplastron; 1 = between hyoplastron and epiplastronentoplastron.

**Character 236 (Evers & Benson (2018): character 235).** Plastron, hyo-hypoplastra

contact: 0 = contact between hyo-hyoplastra absent or reduced; 1 = extensive contact

between hyo-hyoplastra (even for those taxa with plastral kinesis).

**Character 237 (Evers & Benson (2018): character 236).** Plastron, hyo-hypoplastra

serrations: 0 = serrations on the lateral and medial margins absent or weakly developed;

1 = strong serrations along medial and lateral margins present.

**Character 238 (Evers & Benson (2018): character 237).** Axillar and inguinal

notches: 0 = deep U or V-shaped axillar and inguinal notches; 1 = very shallow axillar

and inguinal notches, and long lateral edges.

**Character 239 (Evers & Benson (2018): character 238).** Entoplastron: 0 = present;

1 = absent.

**Character 240 (Evers & Benson (2018): character 239).** Entoplastron, anterior

entoplastral process: 0= present, medial contact of epiplastra absent; 1 = absent, medial

contact of epiplastra present.

**Character 241 (Evers & Benson (2018): character 240).** Entoplastron, size of the

posterior entoplastral process: 0 = posterior process long, reaching as far posteriorly as

the mesoplastra; 1 = posterior process reduced in length.

**Character 242 (Evers & Benson (2018): character 241).** Entoplastron, distinct

posterolateral process: 0 = present; 1 = absent.

**Character 243 (Evers & Benson (2018): character 242).** Entoplastron, shape of the

entoplastron in ventral view: 0 = dagger-shaped; 1 = massive diamond-shaped; 2 = Tshaped, longer than wide; 3 = T-shaped, wider than long, forming broad lateral wings; 4

= strap like and V-shaped.

**Character 244 (Evers & Benson (2018): character 243).** Entoplastron, suture with

hyoplastra: 0 = tightly sutured; 1 = lightly sutured to almost absent contact between

both.

**Character 245 (Evers & Benson (2018): character 244).** Epiplastra, shape and

contact of epiplastra: 0 = epiplastra squarish in shape, lack a contact between each other

due to the narrow participation of the entoplastron in the anterior plastral lobe edge; 1

= epiplastra elongate in shape, with medial contact located anterior to the entoplastron;

2 = epiplastra squarish in shape lack of medial contact due to the extensive anterior and

lateral projections of the entoplastron.

**Character 246 (Evers & Benson (2018): character 245).** Epiplastra, very thick

anterior lip in dorsal view: 0 = present; 1 = absent.

**Character 247 (Evers & Benson (2018): character 246).** Hyoplastra, contacts of

axillary buttresses: 0 = absent to slightly contacting peripherals only; 1 = peripherals

and costal 1.

**Character 248 (Evers & Benson (2018): character 247).** Hyoplastra, axillary

buttresses: 0 = present; 1 = ossified axillary buttresses absent.

**Character 249 (Evers & Benson (2018): character 248).** Hyoplastra, termination of

axillary buttresses: 0 = terminates on peripheral 1 or 2; 1 = terminates on peripheral 3;

2 = terminates on peripheral 4 or 5 level. This character is scored as inapplicable for

turtles without axillary buttresses (ch. 247.1).

**Character 250 (Evers & Benson (2018): character 249).** Mesoplastron: 0 = present; 1 = absent.

**Character 251 (Evers & Benson (2018): character 250).** Number of mesoplastra: 0 = two; 1 = one. This character is scored as inapplicable when mesoplastra are absent (ch.

249.1).

**Character 252 (Evers & Benson (2018): character 251).** Mesoplastron, medial

contact of mesoplastra: 0=present, or virtually present when a central plastral fontanelle

is present, absence of contact between hyoplastron and hypoplastron; 1 = absent,

partial contact between hyoplastron and hypoplastron present.

**Character 253 (Evers & Benson (2018): character 252).** Hypoplastra, contacts of

inguinal buttresses: 0 = absent to slightly contacting peripherals; 1 = peripheral and

costal 5; 2 = peripheral, costals 5 and 6; 3 = peripherals and costal 4.

**Character 254 (Evers & Benson (2018): character 253).** Hypoplastra, termination

of inguinal buttresses: 0 = peripheral 8; 1 = peripheral 7; 2 = peripheral 6.

**Character 255 (Evers & Benson (2018): character 254).** Xiphiplastra, distinct anal

notch: 0 = absent; 1 = present.

**Character 256 (Evers & Benson (2018): character 255).** Xiphiplastra, shape of

xiphiplastra: 0 = almost triangular to trapezoidal, with lateral straight to convex margin;

1 = rectangular elongated in shape, coupled forming together with the hypoplastron a

very narrow posterior plastral lobe; 2 = narrow struts, separated by the posterior

fontanelle.

**Character 257 (Evers & Benson (2018): character 256).** Plastral scutes: 0 =

present; 1 = absent.

**Character 258 (Evers & Benson (2018): character 257).** Plastral scutes, midline

sulcus: 0 = straight; 1 = distinctly sinuous, at least for part of its length.

**Character 259 (Evers & Benson (2018): character 258).** Gular, number of gulars: 0

= one pair of scutes; 1 = only one scute.

**Character 260 (Evers & Benson (2018): character 259).** Extragulars: 0 = present;

1 = absent.

**Character 261 (Evers & Benson (2018): character 260).** Extagulars, medial contact: 0 = absent; 1 = present, contacting one another anterior to gular(s); 2 = present, contacting one another posterior to gular(s).

**Character 262 (Evers & Benson (2018): character 261).** Extragulars, anterior plastral tuberosities: 0 = present; 1 = absent.

**Character 263 (Evers & Benson (2018): character 262).** Extragulars, restricted to

epiplastra: 0 = present; 1 = absent, extragulars reach the entoplastron.

**Character 264 (Evers & Benson (2018): character 263).** Intergulars: 0 = absent; 1 = present.

**Character 265 (Evers & Benson (2018): character 264).** Humerals, number of pairs: 0 = one pair present; 1 = two pairs present, subdivided by a plastral hinge.

**Character 266 (Evers & Benson (2018): character 265).** Humerals, humeropectoral sulcus: 0 = restricted to hyoplastra; 1 = crossing the posterior portion of entoplastron.

**Character 267 (Evers & Benson (2018): character 266).** Pectorals: 0 = present; 1 = absent.

**Character 268 (Evers & Benson (2018): character 267).** Pectorals, anteroposteriorly

developed: 0 = present; 1 = absent, very short antero-posterior development.

**Character 269 (Evers & Benson (2018): character 268).** Abdominals: 0 = present;

1 = absent.

**Character 270 (Evers & Benson (2018): character 269).** Abdominals, medial contact to one another: 0 = present; 1 = absent. This character is scored as inapplicable for turtles that lack abdominals.

**Character 271 (Evers & Benson (2018): character 270).** Anals: 0 = only cover parts of the xiphiplastra; 1 = overlap anteromedially onto the hypoplastra.

**Character 272 (Evers & Benson (2018): character 271).** Inframarginals: 0 = present; 1 = absent.

*Remarks*: Evers & Benson (2018) score *Toxochelys* as lacking inframarginal scutes (state 1). Inframarginal scutes can be found in *Toxochelys latiremis*: ROM 28563 and this score is adjusted here to reflect that (state 0).

**Character 273 (Evers & Benson (2018): character 272).** Number of

inframarginals: 0 = more than two pair present, plastral scales do not contact marginals;

1 = two pair present (axillaries and inguinals), limited contact between plastral scales

and marginals present. This character is scored as inapplicable when inframarginals are

absent (ch. 271.1).

**Character 274 (Evers & Benson (2018): character 273).** Cervical ribs: 0 = large cervical ribs present; 1 = cervical ribs reduced or absent.

**Character 275 (Evers & Benson (2018): character 274).** Cervicals, position of the transverse processes: 0 = middle of the centrum; 1 = anterior end of the centrum.

**Character 276 (Evers & Benson (2018): character 275).** Cervicals, posterior

cervicals with strongly developed ventral keels: 0 = absent or slightly developed in all

vertebrae; 1 = present, more developed on posterior vertebrae.

**Character 277 (Evers & Benson (2018): character 276).** Cervicals, cervical 8 centrum significantly shorter than cervical 7: 0 = absent; 1 = present.

**Character 278 (Evers & Benson (2018): character 277).** Cervicals, triangular diapophyses: 0 = absent; 1 = present.

**Character 279 (Evers & Benson (2018): character 278).** Cervicals, central articulations of cervical vertebrae: 0 = articulations not formed, cervical vertebrae amphicoelous or platycoelous; 1 = articulations formed, cervical vertebrae procoelous or opisthocoelous.

**Character 280 (Evers & Benson (2018): character 279).** Cervicals, articulation between cervical 8 and dorsal vertebrae 1: 0 = 8 (dorsal 1; 1 = 8) dorsal 1; 2 = vertebrae articulate along zygapophyses only.

**Character 281 (Evers & Benson (2018): character 280).** Cervicals, biconvex

cervical vertebrae in the middle of the neck: 0 = absent; 1 = present.

**Character 282 (Evers & Benson (2018): character 281).** Cervicals, biconvex

cervical vertebra in the middle of the neck: 0 = cervical 2; 1 = cervical 3; 2 = cervical 4; 3

= cervical 5.

**Character 283 (Evers & Benson (2018): character 282).** Cervicals, biconcave cervical vertebrae: 0 = absent; 1 = present.

**Character 284 (Evers & Benson (2018): character 283).** Cervicals, double articulation between cervical 5 and 6: 0 = absent; 1 = present.

**Character 285 (Evers & Benson (2018): character 284).** Cervicals, double articulation between cervical 6 and 7: 0 = absent; 1 = present.

**Character 286 (Evers & Benson (2018): character 285).** Cervicals, central articulation between cervical 6 and 7: 0 = cervical 6 concave ( cervical 7 convex; 1 = platycoelous, cervical 6 II cervical 7.

**Character 287 (Evers & Benson (2018): character 286).** Cervicals, double articulation between cervical 7 and 8: 0 = absent; 1 = present.

*Remarks*: Zangerl (1953b) described a double articulation between the 7^th^ and 8^th^ cervical vertebrae of *Toxochelys latiremis* but due to the limited number of specimens with preserved cervical columns, states that the character may be due to individual variation. However, given that this double articulation is present in the most complete cervical series known for the species, *Toxochelys latiremis* is scored state 1, contra Evers and Benson (2018). A double articulation between the 7^th^ and 8^th^ cervical vertebrae is also known from the most complete cervical series of *Ctenochelys stenoporus*: USNM 357166 (Matzke, 2007).

**Character 288 (Evers & Benson (2018): character 287).** Cervicals, height versus

length of centra and neural arch: 0 = total height of centra and neural arch longer than

the anteroposterior length of the cervical centra; 1 = total height of centra and neural

arch much shorter than the anteroposterior length of the cervical centra.

**Character 289 (Evers & Benson (2018): character 288).** Cervicals, modification of neural arch on cervical 8: 0 = neural arch without modificiation of postzygapophyses; 1

= neural arch with postzygapophyses pointing anteroventrally.

**Character 290 (Evers & Benson (2018): character 289).** Cervicals, postzygapophyses united in midline: 0 = absent; 1 = present.

**Character 291 (Evers & Benson (2018): character 290).** Cervicals, ventral process

on cervical 8: 0 = absent; 1 = present, well developed (as tall or taller than the height of

the centrum).

**Character 292 (Evers & Benson (2018): character 291).** Cervicals, shape of central

articulation of cervicals 7 and 8: 0 = as high as wide; 1 = much wider than high.

**Character 293 (Evers & Benson (2018): character 292).** Ribs, length of first dorsal

rib: 0 = long, extends full length of first costal and may even contact peripherals distally;

1 = intermediate, in contact with well-developed anterior bridge buttresses; 2 =

intermediate to short, extends less than halfway across first costal.

**Character 294 (Evers & Benson (2018): character 293).** Ribs, contact of dorsal

ribs 9 and 10 with costals: 0 = present; 1 = absent.

**Character 295 (Evers & Benson (2018): character 294).** Dorsal rib 10: 0 = long,

spanning full length of costals and contacting peripherals distally; 1 = short, not spanning

father distally than pelvis.

**Character 296 (Evers & Benson (2018): character 295).** Dorsals, anterior articulation of the first dorsal centrum: 0 = faces at most slightly anteroventrally; 1 = faces strongly anteroventrally.

**Character 297 (Evers & Benson (2018): character 296).** Caudals, tail club: 0 = present; 1 = absent.

**Character 298 (Evers & Benson (2018): character 297).** Caudals, anterior caudal centra: 0 = amphicoelous; 1 = procoelous or platycoelous; 2 = opisthocoelous.

**Character 299 (Evers & Benson (2018): character 298).** Caudals, posterior caudal

centra: 0 = amphicoelous; 1 = procoelous or platycoelous: 2 = opisthocoelous.

**Character 300 (Evers & Benson (2018): character 299).** Caudals, chevrons: 0 = present on nearly all caudal vertebrae: 1 = absent, or only poorly developed, along the posterior caudal vertebrae.

**Character 301 (Evers & Benson (2018): character 300).** Caudals, tail ring: 0 = absent; 1 = present.

**Character 302 (Evers & Benson (2018): character 301).** Scapula, anterodorsal ridge of acromion: 0 = present; 1 = absent.

**Character 303 (Evers & Benson (2018): character 302).** Scapula, ventral ridge of acromion: 0 = present; 1 = absent developed proximally near glenoid.

**Character 304 (Evers & Benson (2018): character 303).** Scapula, horizontal ridge

of acromion: 0 = well-developed, coracoid foramen present; 1 = reduced, only developed along distal portion of acromion.

**Character 305 (Evers & Benson (2018): character 304).** Scapula, glenoid neck on scapula: 0 = absent; 1 = present.

**Character 306 (Evers & Benson (2018): character 305).** Scapula, lamina between the dorsal process of the scapula and the acromion: 0 = well developed; 1 = reduced; 2 = absent.

**Character 307 (Evers & Benson (2018): character 306).** Scapula, internal angle between acromion process and scapular process ≥110°: 0 = absent; 1 = present.

**Character 308 (Evers & Benson (2018): character 307).** Coracoid, coracoid vs humerus length: 0 = shorter than humerus; 1 = at least as long as humerus.

*Remarks*: Evers & Benson (2018) score *Toxochelys sp.* as state 0 for this character. A relatively complete juvenile individual of *Toxochelys latiremis*: FMNH PR123 has a coracoid that is 138 mm long and a humerus that is 129 mm (Zangerl, 1953; pers. obs.). *Toxochelys latiremis* is scored here as state 1.

**Character 309 (New character).** Coracoid, maximum width ≥ 3 times the minimum width: 0 = present; 1 = absent.

*Remarks*: This character encodes the variation present in the width of the posterior process of the coracoid. In most turtles, the posterior process of the coracoid is greatly expanded mediolaterally and forms a broad, posteriorly convex blade (state 0). However, in certain crown-group cheloniids and protostegids, the posterior process is only slightly wider than the diaphysis resulting in an almost rod-like coracoid (state 1).


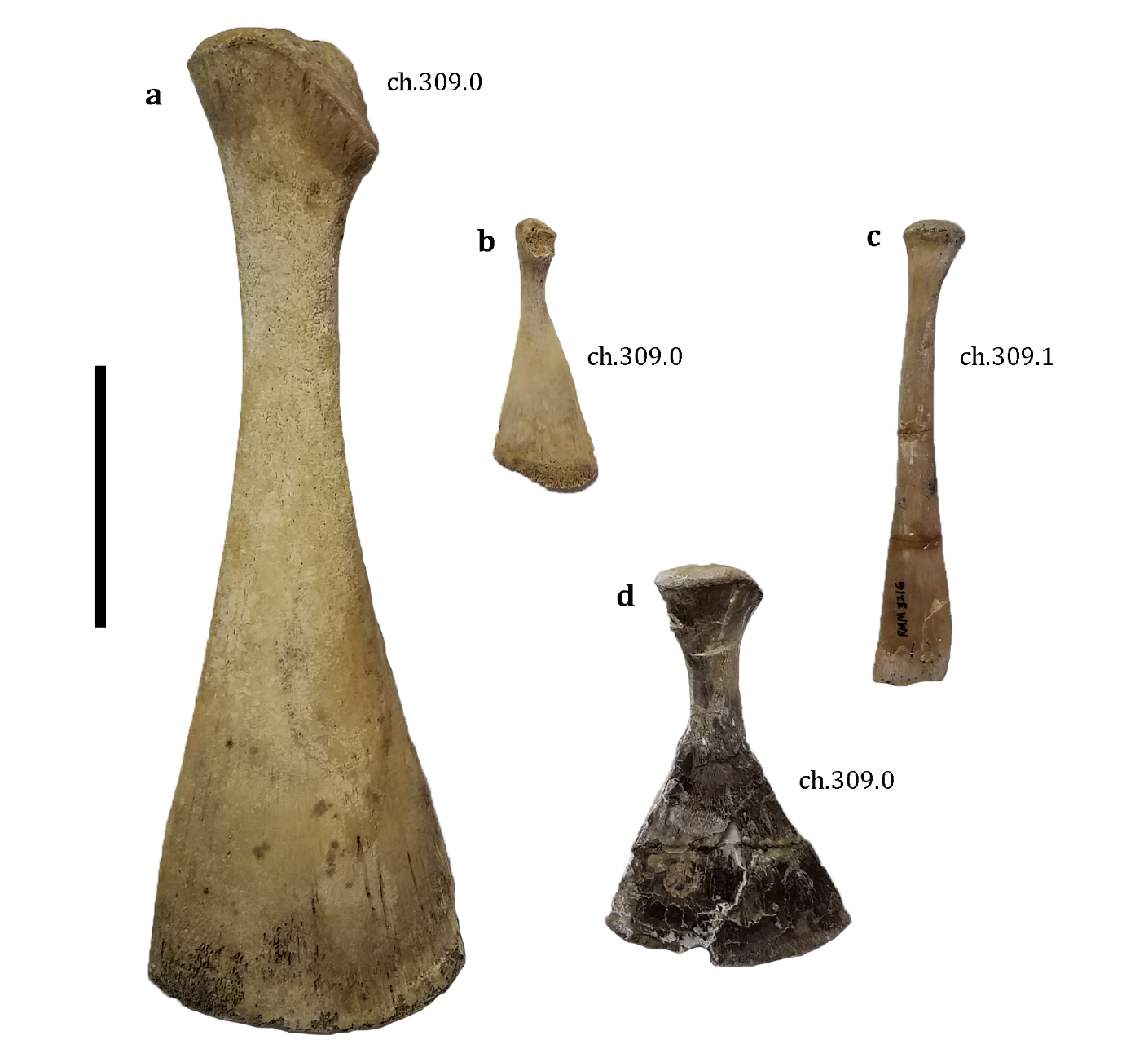


**Fig. S15.** Coracoids of various living and fossil turtles in ventral view illustrating the variation in the width of the posterior process. (**a)** *Chelonia mydas* (MSC 20455), (**b)** *Chelydra serpentina* (MSC 20968), (**c**) *Calcarichelys gemma* (MSC 3216), (**d**) *Prionochelys matutina* (MSC 1540). Scale bar = 5 cm.

**Character 310 (Evers & Benson (2018): character 308).** Coracoid, foramen: 0 = present; 1 = absent.

**Character 311 (Evers & Benson (2018): character 309).** Cleithrum: 0 = present; 1 = absent.

**Character 312 (Evers & Benson (2018): character 310).** Cleithrum, contact with

carapace: 0 = present; 1 = osseous contact with carapace absent.

**Character 313 (Evers & Benson (2018): character 311).** Pelvis, pelvis-shell attachment: 0 = pelvis-shell attachment by ligaments; 1 = pelvis attached by strong sutural contact of the ischium and pubis with the plastron, and illium with the carapace.

**Character 314 (modified from Evers & Benson (2018)).** Pelvis, thyroid fenestra: 0

= coalescent; 1 = two separated fenestrae.

*Remarks*: Mulder (2003) described several nearly complete specimens of the Maastrichtian chelonioid *Allopleuron hofmanni* including one which possesses a nearly complete pelvis (IRScNB 3668, pl. 43, p. 83) which clearly exhibits thyroid fenestrae separated by a contact between the posteromedial process of the pubis and an anteromedial process of the ischium. Contra Ever and Benson (2018), *Allopleuron hofmanni* is scored here as state 1. Evers & Benson (2018) also *Caretta caretta* as having a single thyroid fenestra, however, this is not the case (Wyneken, 2001). We score *Caretta caretta* as having two thyroid fenestrae (state 1).

**Character 315 (Evers & Benson (2018): character 313).** Ilium, elongated iliac neck: 0 = absent; 1 = present.

*Remarks*: Evers & Benson (2018) score *Toxochelys sp.* as unknown (?) for this character. An elongate iliac neck is present in *Toxochelys latiremis* (ROM 25683, Nicholls, 1988, Fig. 1, p.182) and in YPM 3602 (see discussion of YPM 3602 pelvic elements in Zangerl, 1953b, p. 184). The scoring for *Toxochelys latiremis* is recorded here as state 1.

**Character 316 (Evers & Benson (2018): character 314).** Ilium, iliac scar: 0 =

extends from costals onto the peripherals and pygal; 1 = positioned on costals only.

**Character 317 (Evers & Benson (2018): character 315).** Ilium, shape of the ilium

articular site on the visceral surface of the carapace: 0 = narrow and pointed posteriorly;

1 = oval.

**Character 318 (Evers & Benson (2018): character 316).** Ilium, posterior notch in acetabulum: 0 = absent; 1 = present.

**Character 319 (Evers & Benson (2018): character 317).** Ilium, thelial process: 0 =

absent; 1 = present.

**Character 320 (Evers & Benson (2018): character 318).** Pubis, lateral process: 0 =

small, poorly developed, columnar; 1 = well developed and flat.

**Character 321 (Evers & Benson (2018): character 319).** Pubis, epipubis process: 0

= osseus or calcified; 1 = cartilaginous or absent.

**Character 322 (Evers & Benson (2018): character 320).** Ischium, ischial contacts

with plastron: 0 = contact via a large central tubercle; 1 = contact via two seperate

ischial processes.

**Character 323 (Evers & Benson (2018): character 321).** Ischium, lateral process of

ischium or metischial process: 0 = absent; 1 = present.

**Character 324 (Evers & Benson (2018): character 322).** Hypoischium: 0 = present;

1 = absent.

**Character 325 (modified from Evers & Benson (2018)).** Humerus, ectepicondylar

foramen: 0 = visible in dorsal view within in a well-defined channel or canal; 1 = only a groove visible in dorsal view.

**Character 326 (Evers & Benson (2018): character 324).** Humerus, proximal

articular surface of humerus: 0 = with shoulder on preaxial side, upturned; 1 = without shoulder, not upturned.

**Character 327 (Evers & Benson (2018): character 325).** Humerus, lateral process

of humerus: 0 = abuts caput humeri; 1 = slightly separated from caput humeri; 2 =

located distal to caput humeri but along proximal end of shaft; 3 = located at middle of

humeral shaft.

**Character 328 (Evers & Benson (2018): character 326).** Humerus, lateral process

of humerus: 0 = visible in dorsal view: 1 = not visible in dorsal view.

**Character 329 (Evers & Benson (2018): character 327).** Humerus, lateral process

shape: 0 = rounded to slightly squared; 1 = V-shaped or triangular.

**Character 330 (Evers & Benson (2018): character 328).** Humerus, expansion of

lateral process: 0 = limited to anterior surface of shaft; 1 = expands onto ventral surface.

**Character 331 (Evers & Benson (2018): character 329).** Humerus, medial concavity of lateral process: 0 = absent; 1 = present.

**Character 332 (Evers & Benson (2018): character 330).** Humerus, prominent anterior projection of lateral process: 0 = absent; 1 = present.

**Character 333 (Evers & Benson (2018): character 331).** Humerus, length of the

humerus versus the width of the proximal end: 0 = two times or less the width of the

proximal end: 1 = more than two times the width of the proximal end.

*Remarks*: Evers & Benson (2018) score *Toxochelys sp.* as unknown (?) for this character. There are several humeri known for both species of *Toxochelys* and in the most complete specimen of *Toxochelys latiremis* (ROM 28563) the length of the humerus is well over two times the width of the proximal end. *Toxochelys latiremis* is scored here as state 1.

**Character 334 (Evers & Benson (2018): character 332).** Humerus, scar for Muscle

latissimus dorsi and Muscle teres major: 0 = located anterior to humeral shaft; 1 =

located at middle of shaft.

**Character 335 (Evers & Benson (2018): character 333).** Humerus, humerus length

vs femur length: 0 = shorter than femur; 1 = longer than femur.

**Character 336 (Evers & Benson (2018): character 334).** Ulna, contact with radius

through rugosity and ridge: 0 = absent; 1 = present.

**Character 337 (Evers & Benson (2018): character 335).** Radius, curves towards

anterior: 0 = absent; 1 = present.

**Character 338 (Evers & Benson (2018): character 336).** Manus, phalangeal formula of the manus: 0 = most digits with two shortenened phalanges: 1 = most digits with three elongated phalanges.

**Character 339 (Evers & Benson (2018): character 337).** Manus, rigid articulations

in 1st and 2nd digit: 0 = absent; 1 = present.

**Character 340 (Evers & Benson (2018): character 338).** Manus, rigid articulations

in 3rd to 5th digit: 0 = absent; 1 = present.

**Character 341 (Evers & Benson (2018): character 339).** Manus, flippers: 0 =

absent; 1 = short flippers present; 2 = elongate flippers present.

**Character 342 (Evers & Benson (2018): character 340).** Ulnare, size of the ulnare

vs the intermedium: 0 = smaller than intermedium: 1 = nearly as large as intermedium;

2 = much larger than intermedium.

**Character 343 (Evers & Benson (2018): character 341).** Pes, number of digits: 0 =

five; 1 = four.

**Character 344 (Evers & Benson (2018): character 342).** Manus and Pes, flattening

of carpals and tarsal elements: 0 = absent; 1 = present.

**Character 345 (Evers & Benson (2018): character 343).** Manus and Pes, hyperphalangy manus digits 4 and 5, pes digit 4: 0 = absent; 1 = present.

**Character 346 (Evers & Benson (2018): character 344).** Femur, femoral trochanters: 0 = distinct, and separated from one another; 1 = fossa obliterated, space between trochanters not concave, but notch present; 2 = fossa obliterated, trochanters connected by bony ridge without a notch.

**Character 347 (Evers & Benson (2018): character 345).** Tibia, tibial pit for pubotibialis and flexor tibialis internus muscles: 0 = absent; 1 = present.

**3.2 Molecular constraint tree**

**
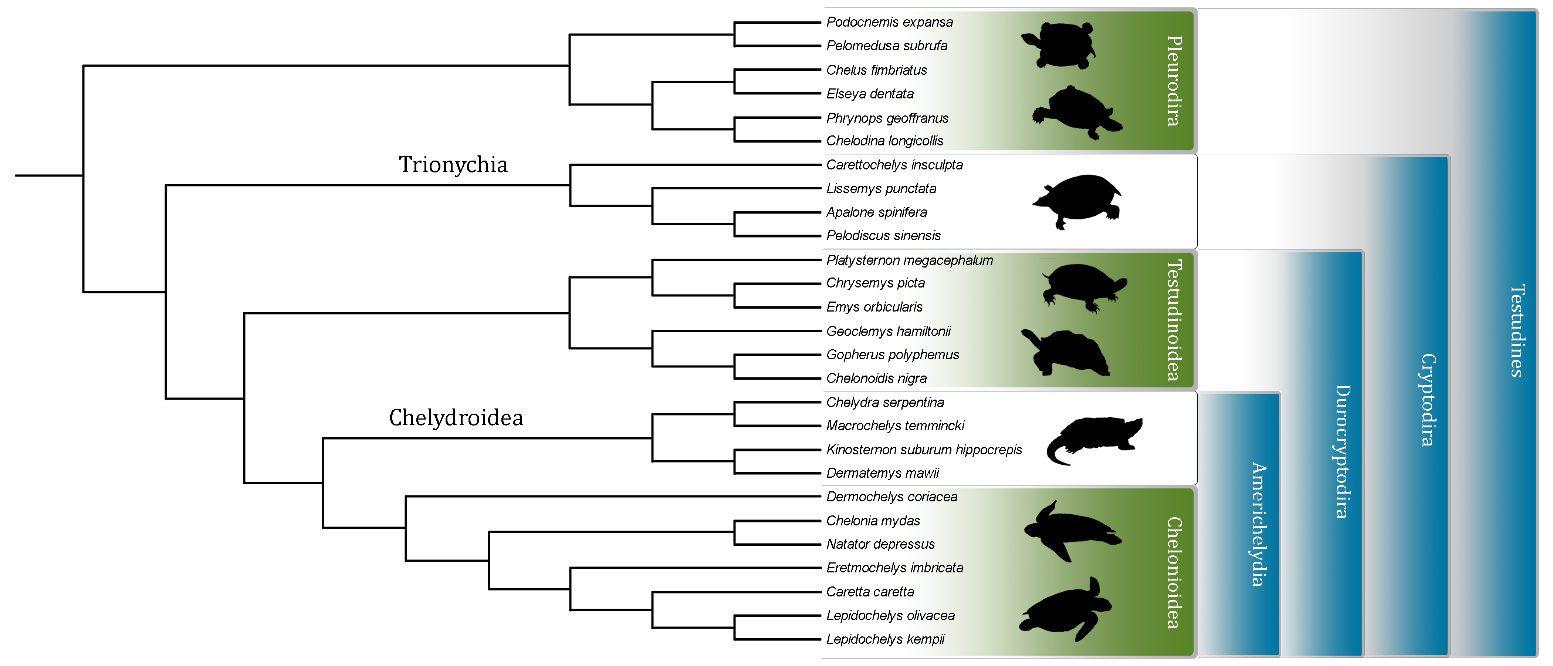
**

**Fig. S16.** Molecular constraint tree of extant taxa used in all phylogenetic analyses. Topology based on Pereira et al., 2017.

**3.3 Ordered characters**

7, 18, 58, 67, 74, 76, 79, 93, 94, 98, 103, 130, 145, 147, 157, 206, 218, 254, 293, 306, 327, 341, 342, 346

**3.4 Unweighted parsimony strict consensus**


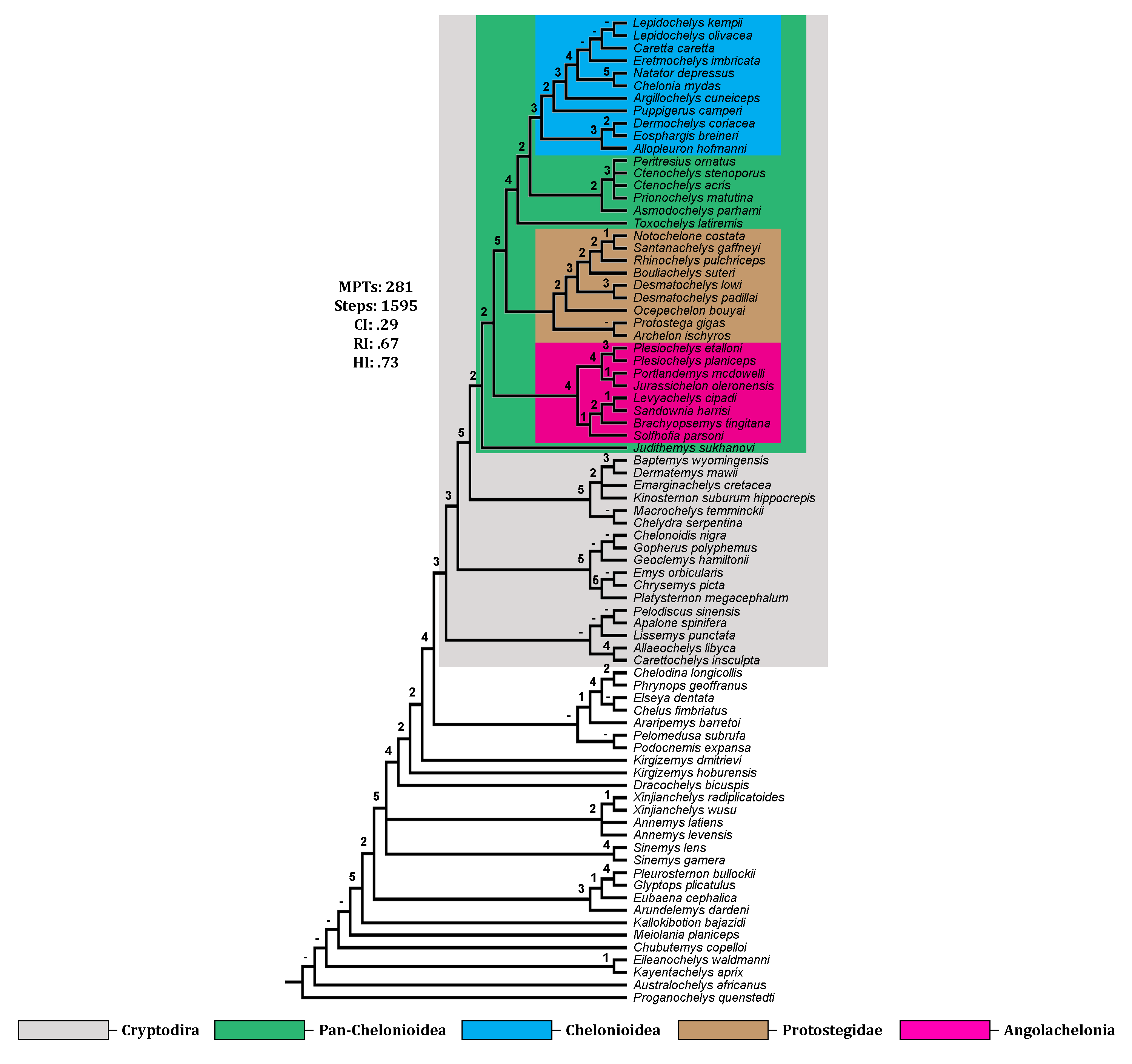


**Fig. S17.** Pruned strict consensus of 281 MPTs resulting from the unweighted parsimony analysis including Bremer decay values. Dashes indicate Bremer scores ≥ 6.

**3.5 Bayesian 50% Majority rule consensus**

**
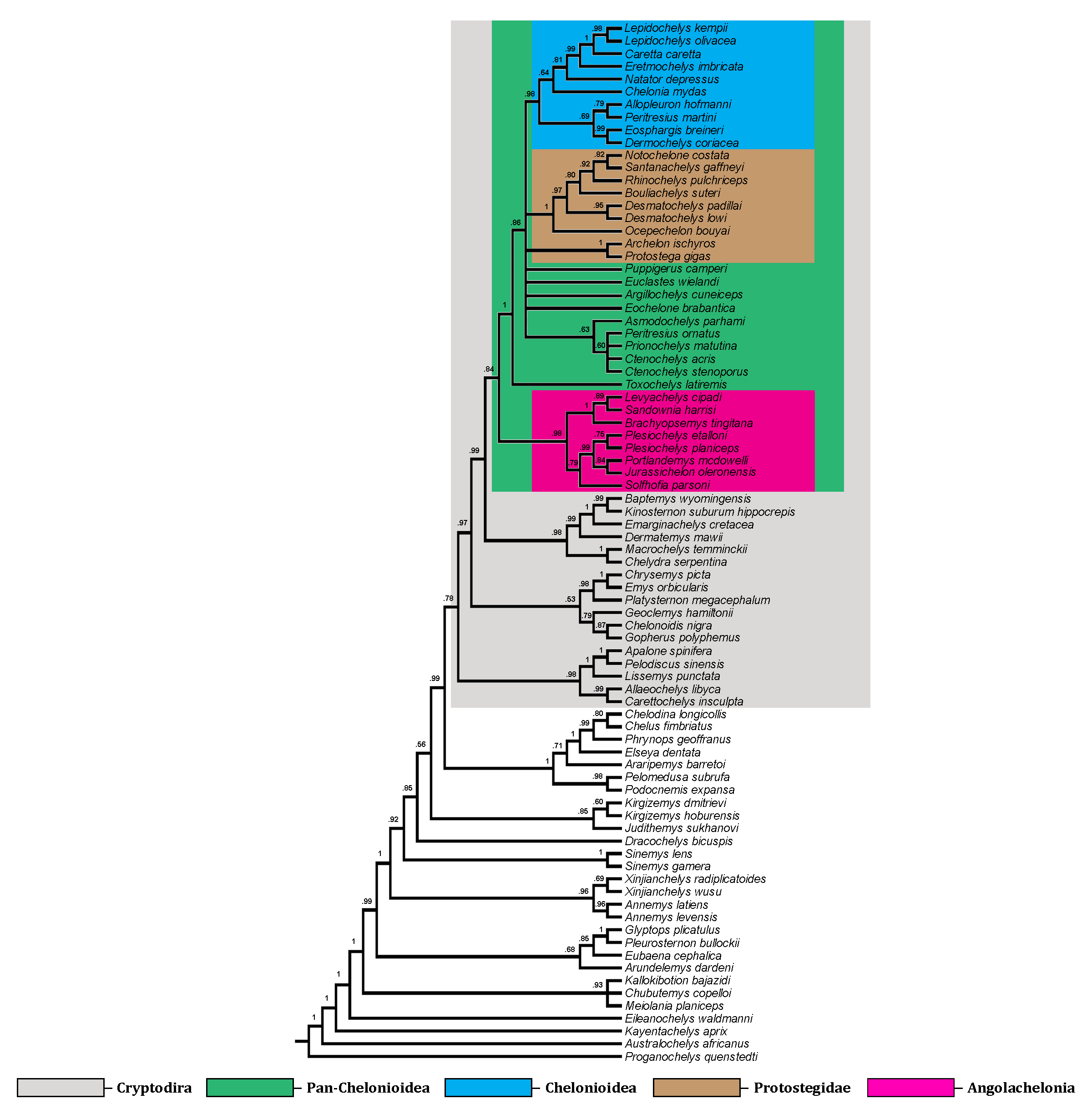
**

**Fig. S18.** Bayesian 50% majority-rule consensus tree with posterior probabilities shown for each resolved node.

**4. Probability calculations of ancestral areas for nodes within Pan-Chelonioidea**

**4.1 Probability list**

**Abbreviations:** a, Asia; e, Europe; f, Africa; n, North America; P, probabilities; s, South America; u, Australia

**Node 1**

P (*Plesiochelys etalloni*) × P (*Plesiochelys planiceps*)

Step 1: 1/1 e × 1/1 e

Step 2: 1 e^2^

Step 3: 1 e = **e, 100%**

**Node 2**

P (*Jurassichelon olenensis*) × P (node 1)

Step 1: 1/1 e × 1/1 e

Step 2: 1 e^2^

Step 3: 1 e = **e, 100%**

**Node 3**

P (*Portlandemys mcdowelli*) × P (node 2)

Step 1: 1/1 e × 1/1 e

Step 2: 1 e^2^

Step 3: 1 e = **e, 100%**

**Node 4**

P (*Solnhofia parsoni*) × P (node 3)

Step 1: 1/1 e × 1/1 e

Step 2: 1 e^2^

Step 3: 1 e = **e, 100%**

**Node 5**

P (node 4) × P (node 7)

Step 1: (1/1 e) × (1/2 f, 1/4 s, 1/4 e)

Step 2: 1/2 ef, 1/4 es, 1/4 e^2^

Step 3: 1/4 e + 1/8 e + 1/4 e, 1/4 f, 1/8 s

Step 4: 5/8 e, 1/4 f, 1/8 s = **e, 62.5%; f, 25%; s, 12.5%**

**Node 6**

P (*Levyachelys cipadi*) × P (*Sandownia harrisi*)

Step 1: 1/1 s × 1/1 e

Step 2: 1 se

Step 3: 1/2 s, 1/2 e = **s, 50%; e, 50%**

**Node 7**

P (*Brachyopsemys tingitana*) × P (node 6)

Step 1: 1/1 f × (1/2 s, 1/2 e)

Step 2: 1/2 fs, 1/2 fe

Step 3: 1/4 f + 1/4 f, 1/4 s, 1/4 e

Step 4: 1/2 f, 1/4 s, 1/4 e = **f, 50%; s, 25%: e, 25%**

**Node 8**

P (node 5) × P (node 17)

Step 1: (5/8 e, 1/4 f, 1/8 s) × (11/16 n, 7/64 e, 1/8 f, 5/128 u, 5/128 s)

Step 2: 55/128 en, 35/512 e^2^, 5/64 ef, 25/1024 eu, 25/1024 es, 11/64 fn, 7/256 fe, 1/32 f^2^, 5/512 fu, 5/512 fs, 11/128 sn, 7/512 se, 1/64 sf, 5/1024 su, 5/1024 s^2^

Step 3: 55/256 e + 35/512 e + 5/128 e + 25/2048 e + 25/2048 e + 7/512 e + 7/1024 e, 5/128 f + 11/128 f + 7/512 f + 1/32 f + 5/1024 f + 5/1024 f + 1/128 f, 25/2048 s + 5/1024 s + 11/256 s + 7/1024 s + 1/128 s + 5/2048 s + 5/1024 s, 25/2048 u + 5/1024 u + 5/2048 u, 55/256 n + 11/128 n + 11/256 n

Step 4: 47/128 e, 187/1024 f, 21/256 s, 5/256 u, 11/32 n ≈ **e, 37%; f, 18%; s, 8%; u, 2%; n, 35%**

**Node 9**

P (*Notochelone costata*) × P (*Santanchelys gaffneyi*)

Step 1: 1/1 u × 1/1 s

Step 2: 1 us

Step 3: 1/2 u, 1/2 s = **u, 50%; s, 50%**

**Node 10**

P (*Rhinochelys pulchriceps*) × P (node 9)

Step 1: 1/1 e × (1/2 u, 1/2 s)

Step 2: 1/2 eu, 1/2 es

Step 3: 1/4 e + 1/4 e, 1/4 u, 1/4 s

Step 4: 1/2 e, 1/4 u, 1/4 s = **e, 50%; u, 25%; s, 25%**

**Node 11**

P (*Bouliachelys suteri*) × P (node 10)

Step 1: 1/1 u × (1/2 e, 1/4 u, 1/4 s)

Step 2: 1/2 ue, 1/4 u^2^, 1/4 us

Step 3: 1/4 u + 1/4 u + 1/8 u, 1/4 e, 1/8 s

Step 4: 5/8 u, 1/4 e, 1/8 s = **u, 62.5%; e, 25%; s, 12.5%**

**Node 12**

P (node 11) × P (node 13)

Step 1: (5/8 u, 1/4 e, 1/8 s) × (1/2 s, 1/2 n)

Step 2: 5/16 us, 5/16 un, 1/8 es, 1/8 en, 1/16 s^2^, 1/16 sn

Step 3: 5/32 u + 5/32 u, 5/32 s + 1/16 s + 1/16 s + 1/32 s, 1/16 e + 1/16 e, 5/32 n + 1/16 n + 1/32 n

Step 4: 5/16 u, 5/16 s, 1/8 e, 1/4 n = **u, 31.25%; s, 31.25%; e, 12.5%; n, 25%**

**Node 13**

P (*Desmatochelys padillai*) × P (*Desmatochelys lowi*)

Step 1: 1/1 s × 1/1 n

Step 2: 1 sn

Step 3: 1/2 s, 1/2 n = **s, 50%; n, 50%**

**Node 14**

P (*Ocepechelon bouyai*) × P (node 12)

Step 1: 1/1 f × (5/16 u, 5/16 s, 1/8 e, 1/4 n)

Step 2: 5/16 fu, 5/16 fs, 1/8 fe, 1/4 fn

Step 3: 5/32 f + 5/32 f + 1/16 f + 1/8 f, 5/32 u, 5/32 s, 1/16 e, 1/8 n

Step 4: 1/2 f, 5/32 u, 5/32 s, 1/16 e, 1/8 n = **f, 50%; u, 15.625%; s, 15.625%; e, 6.25%; n, 12.5%**

**Node 15 (Protostegidae)**

P (node 16) × P (node 14)

Step 1: 1/1 n × (1/2 f, 5/32 u, 5/32 s, 1/16 e, 1/8 n)

Step 2: 1/2 nf, 5/32 nu, 5/32 ns, 1/16 en, 1/8 n^2^

Step 3: 1/4 n + 5/64 n + 5/64 n + 1/32 n + 1/8 n, 1/4 f, 5/64 u, 5/64 s, 1/32 e

Step 4: 9/16 n, 1/4 f, 5/64 u, 5/64 s, 1/32 e = **n, 56.25%; f, 25%; u, 7.8125%; s, 7.8125%; e, 3.125%**

**Node 16**

P (*Protostega gigas*) × **(***Archelon ischyros***)**

Step 1: 1/1 n × 1/1 n

Step 2: 1 n^2^

Step 3: 1 n = **n, 100%**

**Node 17**

P (node 15) × P (node 18)

Step 1: (9/16 n, 1/4 f, 5/64 u, 5/64 s, 1/32 e) × (13/16 n, 3/16 e)

Step 2: 117/256 n^2^, 27/256 ne, 13/64 fn, 3/64 fe, 65/1024 un, 15/1024 ue, 65/1024 ns, 15/1024 se, 13/512 en, 3/512 e^2^

Step 3: 117/256 n + 27/512 n + 13/128 n + 65/2048 n + 65/2048 n + 13/1024 n, 27/512 e + 3/128 e + 15/2048 e + 15/2048 e + 13/1024 e + 3/512 e, 13/128 f + 3/128 f, 65/2048 u + 15/2048 u, 65/2048 s + 15/2048 s

Step 4: 11/16 n, 7/64 e, 1/8 f, 5/128 u, 5/128 s = **n, 68.75%; e, 10.9375%; f, 12.5%; u, 3.90625%; s, 3.90625%**

**Node 18**

P (*Toxochelys latiremis*) × P (node 21)

Step 1: 1/1 n × (5/8 n, 3/8 e)

Step 2: 5/8 n^2^, 3/8 ne

Step 3: 5/8 n + 3/16 n, 3/16 e

Step 4: 13/16 n, 3/16 e = **n, 81.25%; e, 18.75%**

**Node 19**

P (*Asmodochelys parhami*) × P (node 20)

Step 1: 1/1 n × 1/1 n

Step 2: 1 n^2^

Step 3: 1 n = **n, 100%**

**Node 20**

P (*Ctenochelys acris*) × P (*Ctenochelys stenoporus*) × P (*Prionochelys matutina*) × P (*Peritresius ornatus*) × P (*Peritresius martini*)

Step 1: 1/1 n × 1/1 n × 1/1 n × 1/1 n × 1/1 n

Step 2: 1 n^5^

Step 3: 1 n = **n, 100%**

**Node 21**

P (node 19) × P (node 23)

Step 1: 1/1 n × (3/4 e, 1/4 n)

Step 2: 3/4 ne, 1/4 n^2^

Step 3: 3/8 n + 1/4 n, 3/8 e

Step 4: 5/8 n, 3/8 e = **n, 62.5%; e, 37.5%**

**Node 22**

P (*Allopleuron hofmanni*) × P (*Eosphargis breineri*)

Step 1: 1/1 e × 1/1 e

Step 2: 1 e^2^

Step 3: 1 e = **e, 100%**

**Node 23**

P (node 22) × P (node 24)

Step 1: 1/1 e × (1/2 n, 1/2 e)

Step 2: 1/2 en, 1/2 e^2^

Step 3: 1/4 e + 1/2 e, 1/4 n

Step 4: 3/4 e, 1/4 n = **e, 75%; n, 25%**

**Node 24**

P (*Euclastes wielandi*) × P (node 25)

Step 1: 1/1 n × 1/1 e

Step 2: 1 ne

Step 3: 1/2 n, 1/2 e = **n, 50%; e, 50%**

**Node 25**

P (*Argillochelys cuneiceps*) × P (*Puppigerus camperi*)

Step 1: 1/1 e × 1/1 e

Step 2: 1 e^2^

Step 3: 1 e = **e, 100%**

**4.2 Resolved node numbering**

**
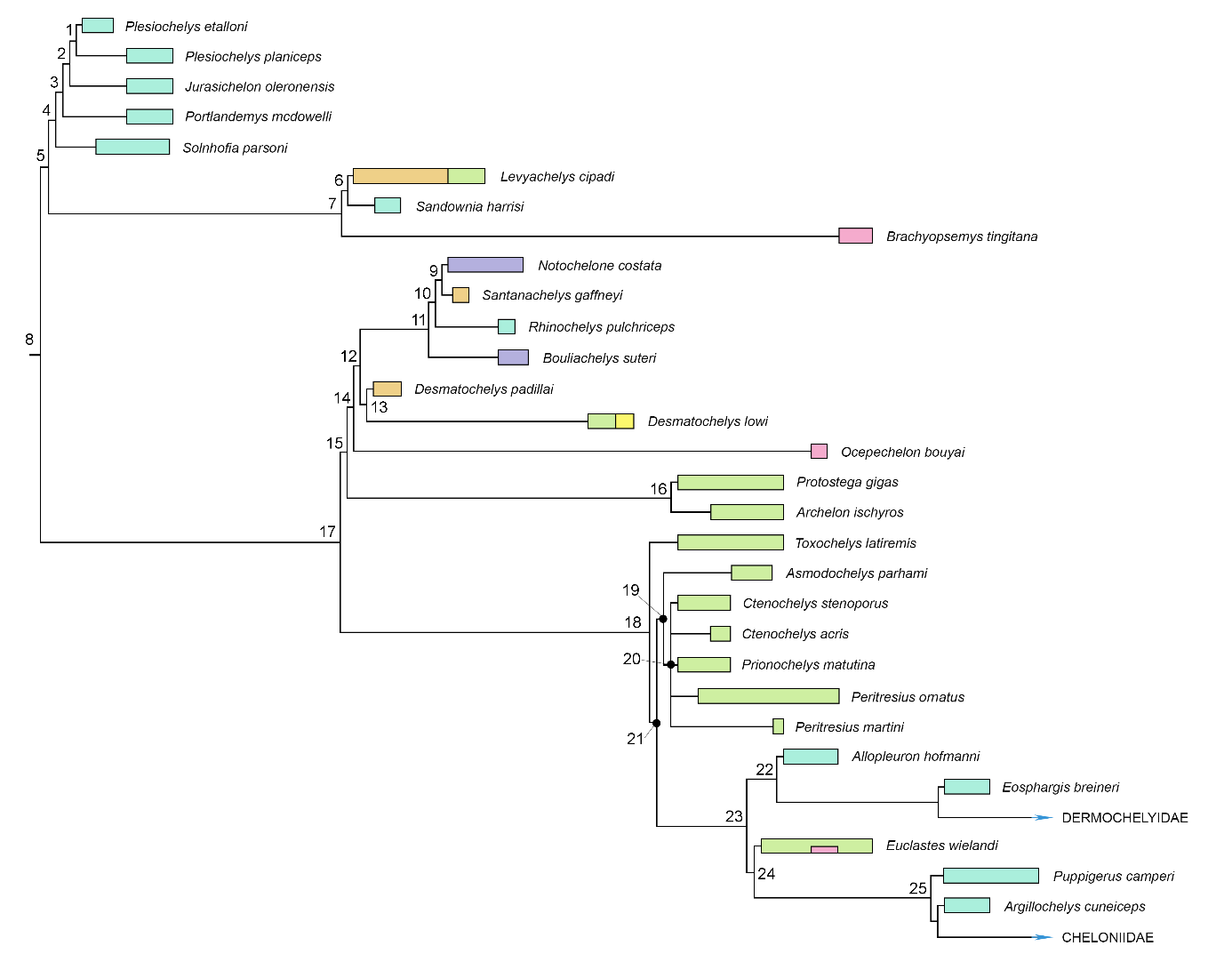
**

**Fig. S19.** Numbering of nodes for which ancestral range probabilities were calculated.

**5. References**

Anquetin, J., Puntener, C., Joyce, W. (2017). A review of the fossil record of turtles of the clade Thalassochelydia. *Bulletin of the Peabody Museum of Natural History* 58(2):317-369.

Baird, D. (1964). A fossil sea-turtle from New Jersey. *New Jersey State Museum Investigations* 1:3-26.

Bardet, N., Jalil, N-E., Lapparent de Broin, F., Germain, D., Lambert, O., Amaghzaz, M. (2013). A giant chelonioid turtle from the Late Cretaceous of Morocco with a suction feeding apparatus unique among tetrapods. *PLoS One* 8(7):e63586

Cadena, E. (2015). The first South American sandownid turtle from the Lower Cretaceous of Colombia. *PeerJ* 3:e1431

Cadena, E. and Parham, J. (2015). Oldest known marine turtle? A new protostegid from the Lower Cretaceous of Colombia. *PaleoBios* 32:1-42.

Collins, J. (1970). The chelonian *Rhinochelys* Seeley from the upper Cretaceous of England and France. *Palaeontology* 13:355-378.

Dockery, D. and Thompson, D. (2016)*.* The Geology of Mississippi*.* *University Press of Mississippi* p. 215.

Evers, S. and Benson, R. (2018). A new phylogenetic hypothesis of turtles with implications for the timing and number of evolutionary transitions to marine lifestyles in the group. *Palaeontology* 62(1):93-134.

Gaffney, E. (1981). A review of the fossil turtles of Australia. *American Museum Novitates* 2720:1-38.

Gentry, A. (2016). New material of the Late Cretaceous marine turtle *Ctenochelys acris* Zangerl, 1953 and a phylogenetic reassessment of the ‘toxochelyid’-grade taxa. *Journal of Systematic Palaeontology* 15(8):675-696.

Gentry, A. (2018). *Prionochelys matutina* Zangerl, 1953 (Testudines: Pan-Cheloniidae) from the Late Cretaceous of the United States and the evolution of epithecal ossifications in marine turtles. *PeerJ* 6:e5876.

Gentry, A., Parham, F., Ehret, D., Ebersole, J. (2018). A new species of *Peritresius* Leidy, 1856 (Testudines: Pan-Cheloniidae) from the Late Cretaceous (Campanian) of Alabama, USA and the occurrence of the genus within the Mississippi Embayment of North America. *PLoS One* 13(4):e0195651.

Hay, O. (1908). The fossil turtles of North America. *Carnegie Institute of Washington* 75: 568pp.

Hirayama, R. (1997). Distribution and diversity of Cretaceous chelonioids. p225-241. In CALLAWAY, J. and NICHOLLS, E. (eds.). Ancient Marine Reptiles. *Elsevier*.

Hirayama, R. (1998). Oldest known sea turtle. *Nature* 392:705-708.

Hirayama, R. and Chitoku, T. (1996). Family Dermochelyidae (superfamily Chelonioidea) from the Upper Cretaceous of north Japan. *Transactions and Proceedings of the Palaeontological Society of Japan* 184:597-622.

Jansen, R., Van Baal, R., and Schulp, A. (2011). On the taphonomy of the late Maastrichtian (Late Cretaceous) marine turtle *Allopleuron hofmanni*. *Netherlands Journal of Geosciences* 90:187-196.

Joyce, W. (2007). Phylogenetic relationships of Mesozoic turtles. *Bulletin of the Peabody Museum of Natural History*. 48:3-102.

Kauffman, E., Sageman, B., Kirkland, J., Elder, W., Harries, P., Villamil, T. (1993). Molluscan biostratigraphy of the Cretaceous Western Interior Basin, North America. p393-434. In CALDWELL, W. and KAUFFMAN, E. (eds.). Evolution of the Western Interior Basin. *Geological Association of Canada, Special Paper 39*.

Kear, B. and Lee, M. (2006). A primitive protostegid from Australia and early sea turtle evolution. *Biology Letters* 2:116-119.

Mancini, E. and Puckett, T. (2005). Jurassic and Cretaceous transgressive-regressive cycles, northern Gulf of Mexico, USA. *Stratigraphy* 2:31-48.

Matzke, A. (2007). An almost complete juvenile specimen of the cheloniid turtle *Ctenochelys stenoporus* (Hay, 1905) from the Upper Cretaceous Niobrara Formation of Kansas, USA. *Palaeontology* 50(3):669-691.

Matzke, A. (2009). Osteology of the skull of *Toxochelys* (Testudines, Chelonioidea). *Palaeontographica, Abteilung A* 288:93-150.

Meylan, P., Moody, R., Walker, C., Chapman, S. (2000). *Sandownia harrisi*, a highly derived trionychid turtle (Testudines: Cryptodira) from the Early Cretaceous of the Isle of Wright, England. *Journal of Vertebrate Paleontology* 20(3):522-532.

Mulder, E. (2003). Comparative osteology, palaeoecology, and systematics of the Late Cretaceous turtle *Allopleuron hofmanni* (Gray, 1831) from the Maastrichtian type area. p23-92. In MULDER, E. On the Late Cretaceous tetrapods from the Maastrichtian type area. *Publicaties van het Natuurhistorich Genootschap in Limburg* 44.

Nicholls, E. (1988). New material of *Toxochelys latiremis* Cope, and a revision of the genus *Toxochelys* (Testudines, Chelonioidea). *Journal of Vertebrate Paleontology* 8:181-187.

Nielsen, E. (1959). Eocene turtles from Denmark. *Dansk Geologisk Forening* 14:96-114.

Parham, J. and Fastovsky, D. (1997). The phylogeny of cheloniid sea turtles revisited. *Chelonian Conservation and Biology* 4:548-554.

Pereira, A., Sterli, J., Moreira, F., and Schrago, C. (2017). Multilocus phylogeny and statistical biogeography clarify the evolutionary history of major lineages of turtles. *Molecular Phylogenetics and Evolution* 113:59-66.

Puckett, T. (1992). Distribution of ostracodes in the Upper Cretaceous (Late Santonian through Middle Maastrichtian) of Alabama and Mississippi. *Gulf Coast Association of Geological Societies Transactions* 42:613-631.

Puckett, T. (2005). Santonian-Maastrichtian planktonic foraminiferal and ostracode biostratigraphy of the northern Gulf Coastal Plain, USA. *Stratigraphy* 2:117-146.

Russell, E., Keady, D., Mancini, E., and Smith, C. (1983). Upper Cretaceous lithostratigraphy and biostratigraphy in northeast Mississippi, southwest Tennessee and northwest Alabama, shelf chalks and coastal clastics. p1-72. In RUSSELL, E., KEADY, D., MANCINI, E., and SMITH, C. (eds.). Spring Field Trip of the Gulf Coast Section of the Society of Economic Paleontologists and Mineralogists and the Alabama Geological Survey, 1983, Guidebook.

Sato, T., Konishi, T., Hirayama, R., and Caldwell, M. (2012). A review of the Upper Cretaceous marine reptiles from Japan. *Cretaceous Research* 37:319-340.

Schwimmer, D., Sanders, A., Erickson, B., and Weems, R. (2015). A Late Cretaceous dinosaur and reptile assemblage from South Carolina, USA. *Part 4, Systematic Paleontology: Transactions of the American Philosophical Society*, 105:43-108.

Sterli, J. and Fuente, M. (2013). New evidence from the Palaeocene of Patagonia (Argentina) on the evolution and palaeo-biogeography of Meiolaniformes (Testudinata, new taxon name). *Journal of Systematic Palaeontology* 11:835-852.

Tong, H. and Meylan, P. (2013). Morphology and relationships of *Brachyopsemys tingitana* gen. et sp. nov. from the Early Paleocene of Morocco and recognition of the new eurocryptodiran turtle family: Sandownidae. p187-212. In BRINKMAN, D., HOLROYD, P., and GARDNER, J. (eds.). Morphology and evolution of turtles. *Springer* 577p.

Weems, R. and Brown, K. (2017). More-complete remains of *Procolpochelys charlestonensis* (Oligocene, South Carolina), and occurrence of *Euclastes* (upper Eocene, South Carolina), and their bearing on Cenozoic pancheloniid sea turtle distribution and phylogeny. *Journal of Paleontology* 91:1228-1243.

Wyneken, J. (2001). The anatomy of sea turtles. *U.S. Department of Commerce NOAA Technical Memorandum NMFS-SEFSC-470* 172p.

Zangerl, R. (1953a). The vertebrate fauna of the Selma Formation of Alabama. Part III. The turtles of the family Protostegidae. *Fieldiana, Geology Memoirs* 3:1-136.

Zangerl, R. (1953b). The vertebrate fauna of the Selma Formation of Alabama. Part IV. The turtles of the family Toxochelyidae. *Fieldiana, Geology Memoirs* 3:137-277.
