# Supplementary material for: Asmodochelys parhami, a new fossil marine turtle from the Campanian Demopolis Chalk and the stratigraphic congruence of competing marine turtle phylogenies
Source: R Soc Open Sci. 2019 Dec 18;6(12):191950. doi: 10.1098/rsos.191950 (PMC6936288; doi:10.1098/rsos.191950)
Supplement: Reviewer comments [file rsos191950_review_history.pdf]

THE ROYAL SOCIETY
PUBLISHING

ROYAL SOCIETY
OPEN SCIENCE

Asmodochelys parhami, a new fossil marine turtle from the Campanian Demopolis Chalk and the stratigraphic congruence of competing marine turtle phylogenies

Andrew D. Gentry, Jun A. Ebersole and Caitlin R. Kiernan

Article citation details
R. Soc. open sci. **6**: 191950.
http://dx.doi.org/10.1098/rsos.191950

Review timeline

Original submission:	6 November 2019
Final acceptance:	26 November 2019

Note: Reports are unedited and appear as submitted by the referee. The review history appears in chronological order.

Note: This manuscript was transferred from another Royal Society journal with peer review.

Review History

Decision letter (RSOS-191950.R0)

26-Nov-2019

Dear Mr Gentry:

It is a pleasure to accept your manuscript entitled "Asmodochelys parhami, a fossil sea turtle from the Campanian Demopolis Chalk and the stratigraphic congruence of competing marine turtle phylogenies" in its current form for publication in Royal Society Open Science. The comments of the reviewer(s) who reviewed your manuscript are included at the foot of this letter.

Please ensure that you send to the editorial office an editable version of your accepted manuscript, and individual files for each figure and table included in your manuscript. You can send these in a zip folder if more convenient. Failure to provide these files may delay the processing of your proof. You may disregard this request if you have already provided these files to the editorial office.

You can expect to receive a proof of your article in the near future. Please contact the editorial

Reports © 2019 The Reviewers; Decision Letters © 2019 The Reviewers and Editors; Responses © 2019 The Reviewers, Editors and Authors. Published by the Royal Society under the terms of the Creative Commons Attribution License http://creativecommons.org/licenses/by/4.0/, which permits unrestricted use, provided the original author and source are credited

office (openscience_proofs@royalsociety.org) and the production office (openscience@royalsociety.org) to let us know if you are likely to be away from e-mail contact -- if you are going to be away, please nominate a co-author (if available) to manage the proofing process, and ensure they are copied into your email to the journal.

Due to rapid publication and an extremely tight schedule, if comments are not received, your paper may experience a delay in publication.

Please see the Royal Society Publishing guidance on how you may share your accepted author manuscript at https://royalsociety.org/journals/ethics-policies/media-embargo/.

Thank you for your fine contribution. On behalf of the Editors of Royal Society Open Science, we look forward to your continued contributions to the Journal.

Kind regards,

Lianne Parkhouse
Editorial Coordinator
Royal Society Open Science
openscience@royalsociety.org

on behalf of Professor Marcelo Sanchez (Associate Editor) and Professor Kevin Padian (Subject Editor).

Follow Royal Society Publishing on Twitter: @RSocPublishing
Follow Royal Society Publishing on Facebook:
https://www.facebook.com/RoyalSocietyPublishing.FanPage/
Read Royal Society Publishing's blog: https://blogs.royalsociety.org/publishing/
